# Genotype inference from aggregated chromatin accessibility data reveals genetic regulatory mechanisms

**DOI:** 10.1186/s13059-025-03538-1

**Published:** 2025-03-30

**Authors:** Brandon M. Wenz, Yuan He, Nae-Chyun Chen, Joseph K. Pickrell, Jeremiah H. Li, Max F. Dudek, Taibo Li, Rebecca Keener, Benjamin F. Voight, Christopher D. Brown, Alexis Battle

**Affiliations:** 1 Genetics and Epigenetics Program, Cell and Molecular Biology Graduate Group, Biomedical Graduate Studies, University of Pennsylvania—Perelman School of Medicine, Philadelphia, PA 19104 USA; 2 Department of Biomedical Engineering, Johns Hopkins University, Baltimore, MD 21218 USA; 3 Department of Computer Science, Johns Hopkins University, Baltimore, MD 21218 USA; 4 Gencove, Inc., New York, NY 11101 USA; 5 Graduate Group in Genomics and Computational Biology, University of Pennsylvania, Philadelphia, PA 19104 USA; 6 Department of Genetics, University of Pennsylvania—Perelman School of Medicine, Philadelphia, PA 19104 USA; 7 Department of Systems Pharmacology and Translational Therapeutics, University of Pennsylvania—Perelman School of Medicine, Philadelphia, PA 19104 USA; 8 Institute for Translational Medicine and Therapeutics, University of Pennsylvania—Perelman School of Medicine, Philadelphia, PA 19104 USA; 9 Department of Genetic Medicine, Johns Hopkins University, Baltimore, MD 21218 USA; 10 Malone Center for Engineering in Healthcare, Johns Hopkins University, Baltimore, MD 21218 USA; 11 Data Science and AI Institute, Johns Hopkins University, Baltimore, MD 21218 USA

## Abstract

**Background:**

Understanding the genetic causes underlying variability in chromatin accessibility can shed light on the molecular mechanisms through which genetic variants may affect complex traits. Thousands of ATAC-seq samples have been collected that hold information about chromatin accessibility across diverse cell types and contexts, but most of these are not paired with genetic information and come from distinct projects and laboratories.

**Results:**

We report here joint genotyping, chromatin accessibility peak calling, and discovery of quantitative trait loci which influence chromatin accessibility (caQTLs), demonstrating the capability of performing caQTL analysis on a large scale in a diverse sample set without pre-existing genotype information. Using 10,293 profiling samples representing 1454 unique donor individuals across 653 studies from public databases, we catalog 24,159 caQTLs in total. After joint discovery analysis, we cluster samples based on accessible chromatin profiles to identify context-specific caQTLs. We find that caQTLs are strongly enriched for annotations of gene regulatory elements across diverse cell types and tissues and are often linked with genetic variation associated with changes in expression (eQTLs), indicating that caQTLs can mediate genetic effects on gene expression. We demonstrate sharing of causal variants for chromatin accessibility across human traits, enabling a more complete picture of the genetic mechanisms underlying complex human phenotypes.

**Conclusions:**

Our work provides a proof of principle for caQTL calling from previously ungenotyped samples and represents one of the largest, most diverse caQTL resources currently available, informing mechanisms of genetic regulation of gene expression and contribution to disease.

**Supplementary Information:**

The online version contains supplementary material available at 10.1186/s13059-025-03538-1.

## Background

Genome wide association studies (GWAS) have identified thousands of loci and common human genetic variants that are associated with a wide range of complex human traits, diseases, and risk factors [1]. GWAS variants are often found in noncoding regions, where they are likely to be involved in gene regulation [2, 3]. However, a full picture of the causal regulatory elements that underlie these associations remains incomplete for most loci [4]. Characterizing the effects of genetic variants on gene expression as revealed by expression quantitative trait locus (eQTL) mapping has provided insights into the molecular basis of phenotypes [3, 5–7]. Although some eQTL variants directly affect open-reading frames, the vast majority are in non-coding regions, as has been described for GWAS variants. Connecting causal variants to the regulatory elements and the genes of action that they perturb remains a central goal of the post-GWAS era.

Accessibility of chromatin regions to transcriptional machinery is a key factor in gene regulation [8, 9]. Genetic variants can affect complex traits through changes in gene expression levels that are mediated by the effect of variants on transcription factor (TF) binding at gene regulatory elements, leading to differences in chromatin accessibility [10, 11]. Improved understanding of the mechanisms involved in chromatin accessibility, revealed by genetic variants that modulate chromatin accessibility (i.e., caQTLs), has the potential to illuminate the molecular mechanisms and genetic regulatory architecture of complex traits. caQTLs have been measured in a variety of tissue and cell types, at both bulk [12–16] and single-cell resolutions [17]. caQTLs have been used in a variety of studies to characterize gene expression regulation [18] and to propose mechanisms for risk loci identified through GWAS [19]. In comparison to eQTLs, caQTLs can identify the direct effect of genetic variants on transcription factor binding at high resolution through techniques such as transcription factor footprinting [20]. eQTLs, however, identify the target gene associated with genetic variants whereas caQTLs inherently do not. In some instances, caQTLs may overlap with eQTLs, providing a more comprehensive understanding of the genetic mechanisms driving GWAS-associated signals. Importantly, caQTLs may be discovered even in the absence of any established eQTL, as eQTL studies may not include the relevant cell type or environmental context to reveal the change to gene expression. Analysis of the contribution of caQTLs to complex human traits can help us better understand the molecular impact of these variants and the mechanism(s) driving GWAS signals. To date, caQTL studies have mostly been performed in analyses restricted to single tissue/cell types, a majority of which have assayed a limited number of samples.

The Assay for Transposase-Accessible Chromatin using sequencing (ATAC-seq) technology has been widely used to capture chromatin accessibility in various cell types and experimental conditions [21–23]. There is a rapidly accumulating trove of ATAC-seq data generated from various experiments, labs, and conditions. This wealth of information has the potential to boost power for caQTL analysis. Unfortunately, many of these samples do not have matched genotype information, a necessary component for QTL analyses. ATAC-seq reads, however, naturally carry the sequence information at nucleotide resolution, providing the possibility of inferring sample genotypes from these data directly.

Here, we have selected and evaluated pipelines to uniformly process ATAC-seq samples, including peak calling and genetic variant calling directly from ATAC-seq reads. We called genotypes using a pipeline incorporating Gencove’s low-pass sequencing methods applied to ATAC-seq reads in accessible chromatin, which utilizes imputation to infer genotype for variants that are located outside of regions covered by observed reads in accessible regions [24, 25]. We benchmarked this pipeline, using gold standard genotype information available for a subset of samples, and compared it against existing methods. Because large-scale public data often contains multiple samples from the same donor or even the same cell line, we also developed a method to automatically infer donor assignment based on genotype from the called variants. Peak calling from thousands of diverse samples presents challenges of identifying true, distinct regions of chromatin accessibility rather than low-signal false positives, or large regions merged from what should be distinct peaks [26, 27]. Based on comparisons across various peak-calling approaches, we finalized a pipeline based on Genrich, using an ATAC-seq specific method [28] for collectively calling peaks across large, diverse data sets and quantifying accessibility in each peak.

Using our ATAC-seq derived genotypes and accessibility estimates across peaks and samples, we then called caQTLs from this collection of publicly available ATAC-seq data. We identified thousands of caQTLs that share a causal signal with GWAS signals, many of which are not explained by known eQTLs. Additionally, we identified many GWAS signals that appear to share a causal signal with both eQTLs and caQTLs, enabling a more comprehensive analysis predicting target gene, gene regulatory element and even potential transcription factors that are driving GWAS signals for a variety of complex human traits. Furthermore, to capture context-specific caQTLs, we inferred clusters of samples with similar accessibility profiles, mostly reflecting cell or tissue type, and identified cluster-specific caQTLs. With the captured global and cluster-specific caQTLs, we investigated potential mechanisms involving transcription factors and their role in target gene regulation.

## Results

### Accurate genotyping and imputation based on ATAC-seq reads from public repositories

**Fig. 1:**
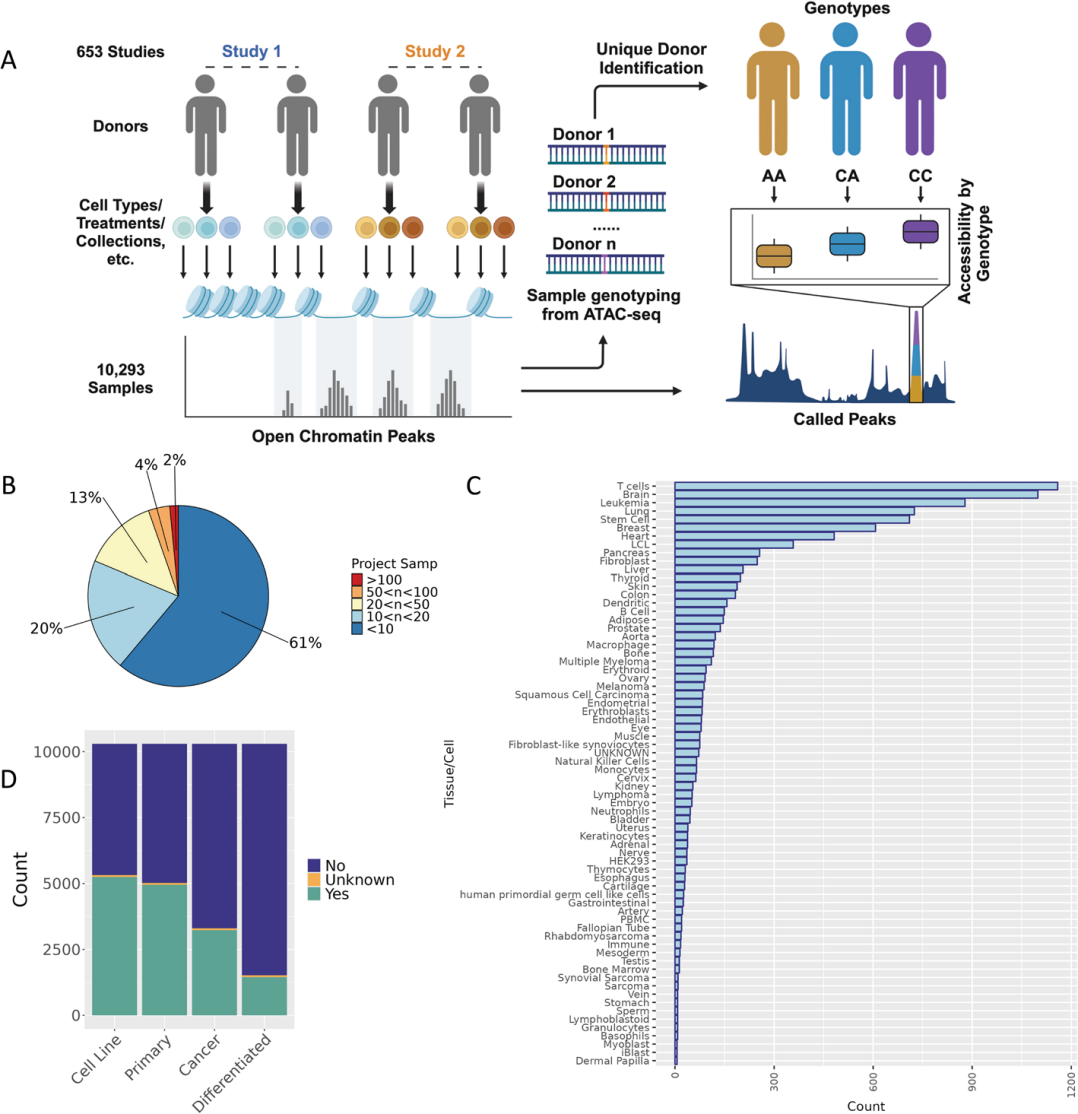
Study overview and characteristics of specimens utilized in this study. **A** Overview of study design to jointly call genotype and caQTLs across studies. Human ATAC-seq datasets were obtained from GEO. After variant-calling (Methods), we identified the unique donors in the dataset (Methods) for use in caQTL mapping. Created with BioRender.com. **B** The distribution of the number of samples collected across all *n* = 653 studies. **C** Frequency of the cell/tissue types present in samples collected across studies based on manual metadata curation. **D** Frequencies of cancer, non-cancer, primary tissues, and cell-line samples included in our study based on our metadata review. For each category, samples were assigned a “Yes” if they belonged to that category (e.g., cell line samples for “Cell Line” category), a “No” if they did not belong (e.g., primary tissue samples for “Cell Line” category), or an “Unknown” if it was not clear from the metadata

We established a workflow to collect a diverse set of publicly available ATAC-seq datasets and ascertain donor genotype from ATAC-seq reads, with the overall objective of mapping genetic variants that are associated with differences in chromatin accessibility for diverse tissues, cell lines, and contexts on a large scale (Fig. 1A). We collected 10,293 human samples from 653 projects from the Gene Expression Omnibus (GEO) data repository, where most projects were comprised of 10 or fewer samples (Fig. 1B, Additional file 1: Table S1). The aggregated data includes samples from a wide variety of tissues or cell types (Fig. 1C). The publicly available data that we collected did not always contain explicit cell/tissue type information readily available, and reporting of cell/tissue type is not performed in a standardized manner across projects. We performed a thorough manual curation of project abstracts, sample labels, and project methods, to annotate each sample with presumed cell/tissue type identity and found that the most common cell/tissue types represented in our study include T cells and brain, among others. Additionally, based on our metadata review, both cancer and normal primary tissue are well represented, along with cell lines and experimentally differentiated cell types (Fig. 1D). The diversity of samples highlights the value of a workflow that can aggregate data and genotype samples from ATAC-seq reads, providing an overall large sample size, but also tissue-specific sample sizes larger than any existing genotyped chromatin accessibility study for several individual tissues including lung, breast, heart, and pancreas [12, 29–31].

**Fig. 2:**
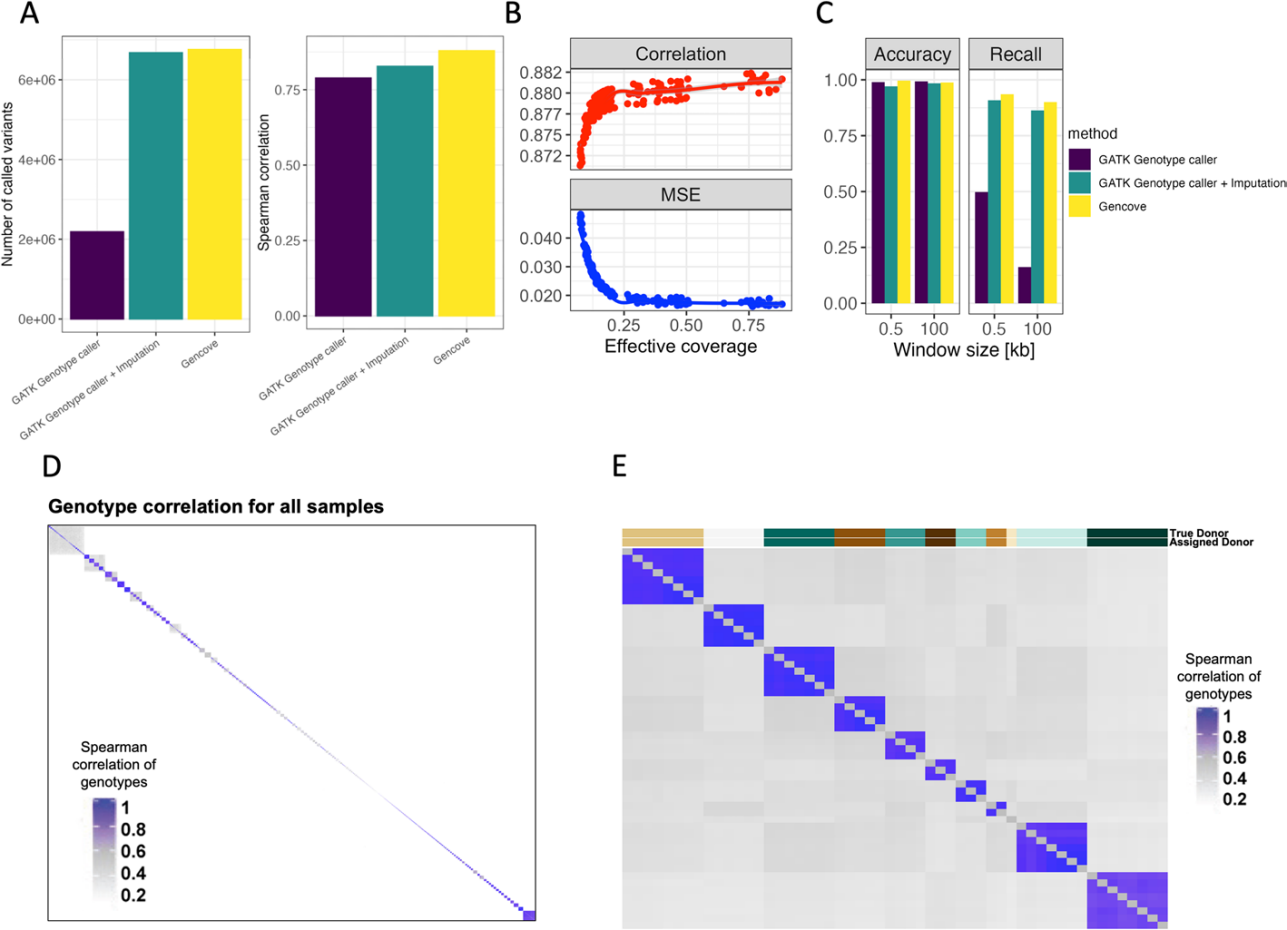
High quality genotyping with unique donor information is inferable directly from reads obtained by ATAC-seq. **A** Variants called for the HapMap samples using multiple pipelines—Gencove, GATK, and GATK with imputation. **B** Accuracy of variant genotype called by Gencove pipeline using a random subset of sample reads. Spearman correlation and mean squared error (MSE) are computed between the called genotype and genotype from WGS. **C** caQTLs called using ATAC-seq derived genotypes across the HapMap samples. **D** Spearman correlation of called genotypes between all samples. **E** Spearman correlation of called genotypes between samples in study PRJNA388006. On the top the “True donor” indicates the donor assignment obtained from metadata information for this study, and “Assigned donor” indicates the donor assignment derived from called genotypes (Methods)

QTL mapping requires paired genotype and molecular phenotype information for each sample. In standard QTL studies, genotyping arrays or whole genome sequencing (WGS) are used to ascertain sample genotype information [32]. Unfortunately, for most of the ATAC-seq data in public repositories that has already been collected, genotype data is not readily available. However, ATAC-seq directly captures genomic DNA fragments from accessible chromatin regions; thus, we surmised that it might instead be possible extract genotype information for these samples directly from the ATAC-seq reads. To obtain genotyping from ATAC-sequencing and evaluate the performance of variant calling using ATAC-seq reads, we applied several approaches: a pipeline incorporating genotyping from Gencove, which optimizes genotyping and imputation for low-pass sequencing data by calculating genotype likelihoods at all positions in the reference panel with at least one read and imputing all genotypes from those likelihoods [24, 25, 33, 34], a standard GATK variant calling pipeline [33, 34], a standard GATK variant calling pipeline followed by imputation, and custom machine learning methods for combining GATK with imputation flexibly based on read depth (Methods, Additional file 2: Fig. S1). To benchmark the performance of our workflow, we used a published dataset of 71 HapMap lymphoblastoid cell lines (LCL) samples with paired ATAC-seq and WGS data [35]. We observed that, compared to the standard GATK variant calling pipeline, the Gencove pipeline with imputation greatly increased the number of variants called and resulted in a median correlation of over 0.88 between true and called donor genotype. Imputation also increased the performance of the GATK pipeline as well (Fig. 2A). To quantify the effects of read coverage on the performance of variant calling, we randomly subselected ATAC-seq reads at varying total read counts for use with the Gencove pipeline. We observed a marginal increase in accuracy with deeper coverage, however, variant-calling accuracy remained high at effective coverage, which is a function of the fraction of polymorphic sites in a reference panel covered by at least one sequencing read [25], as low as 0.04 (Fig. 2B). In our full dataset, the distribution of effective coverage was within the range previously tested with the gold standard HapMap LCL samples, verifying the accuracy of genotype calling in this larger data set. These analyses demonstrate the capabilities of accurate inference of genome-wide genotypes directly from ATAC-seq data.

As a proof of concept, we next performed caQTL mapping using genotypes called from ATAC-seq reads, comparing the results to the caQTLs identified using the full set of gold standard genotypes in these 71 HapMap LCL samples. We observed that caQTL calling using ATAC-seq reads and the Gencove pipeline performed better than the standard GATK pipeline, providing 99% accuracy and over 90% recall compared to caQTL calling using WGS data, while the GATK pipeline followed by imputation performed only slightly worse than Gencove (Fig. 2C). The performance of the Gencove pipeline had substantially greater benefit when testing variants in larger caQTL mapping window sizes where recall remained above 90% for the Gencove pipeline but dropped to 16% for the GATK pipeline at 100 kb, although imputation also somewhat improved the performance of the GATK pipeline (Fig. 2C). Overall, we conclude that genotype calling from ATAC-seq reads leads to highly accurate caQTL calling with relatively high recall and a low rate of false positives. Given the diverse samples collected and varying study designs, an individual donor will likely have multiple ATAC-seq samples represented. As such, we next developed a pipeline to infer unique donors based on the correlation between inferred sample genotypes across different samples and projects (Fig. 2D–E, Methods). Applying this pipeline to all samples, we identified 1454 unique donors across our entire dataset (Additional file 1: Table S2). The majority of donors (~82%) are found within a single project only. As expected, the occurrence of multiple samples per donor was especially common among cell lines, which is reflected in the reduced proportion of cell line samples in the final unique donor sample set (Additional file 2: Fig. S2).

### Peak calling across all samples identifies a plethora of open chromatin regions with regulatory potential

**Fig. 3:**
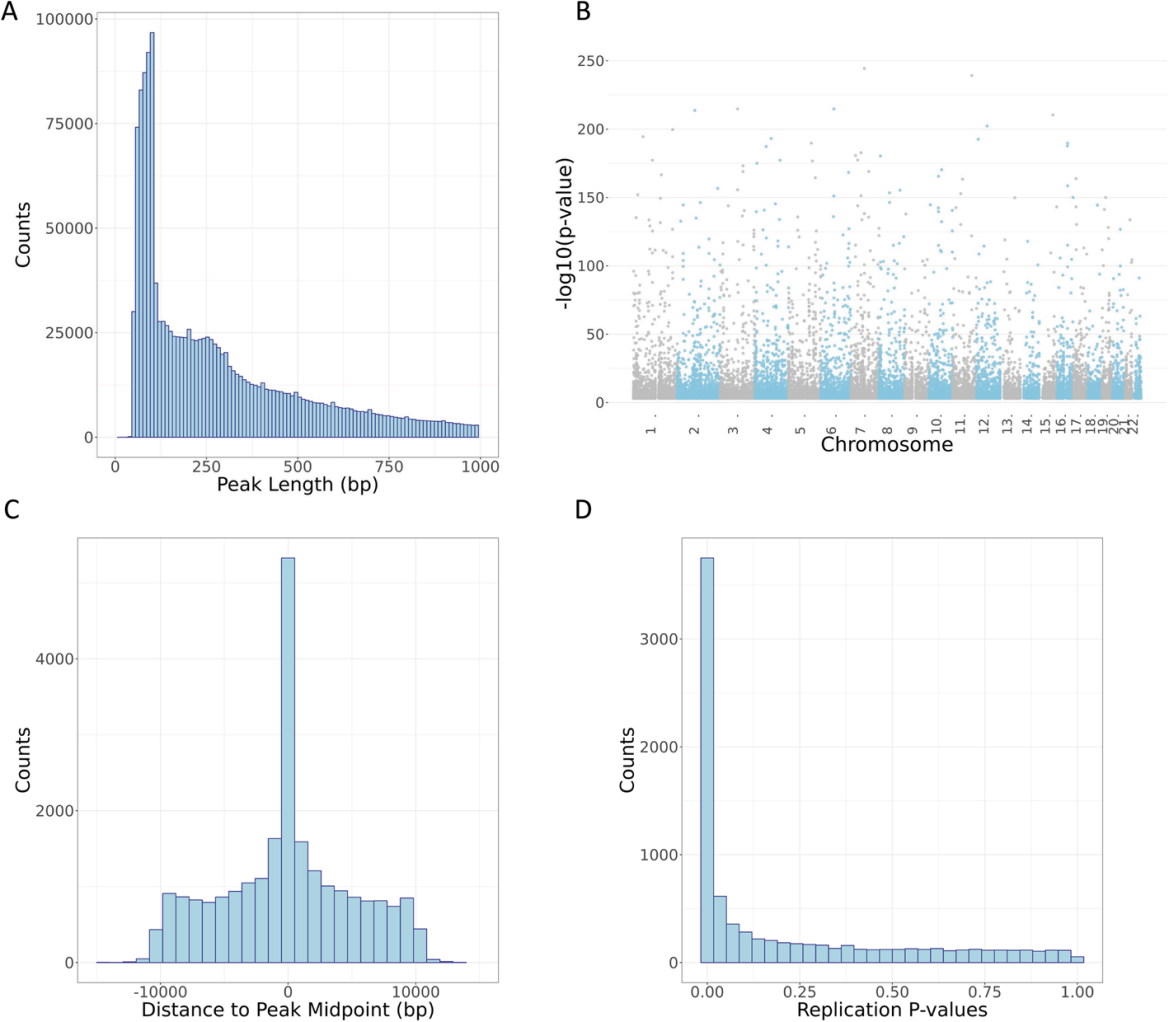
Characteristics of chromatin accessibility peaks and caQTL variants identified in this study. **A** Distribution of peak length across 1,659,379 called peaks (peaks under 1000 bp shown). **B** Manhattan plot of lead variant for 24,159 caQTL peaks. **C** Distance from lead caQTL variant to midpoint of caQTL peak showing elevation of caQTL variant within the identified chromatin accessibility peak. **D** Lead variants for 24,159 caQTL peaks were matched in external caQTL mapping dataset of African LCLs [37]; *p* values from the replication study are plotted here

The next step in our pipeline was to identify open chromatin regions. Multiple strategies have been utilized to call peaks across samples, including calling peaks in each individual sample followed by combining peaks across all samples [36]. To identify a set of consensus peaks in our diverse sample set, we called chromatin accessibility peaks based on evidence across all samples using Genrich, a peak caller optimized for ATAC-seq reads [28]. Genrich assigns *p* values to genomic positions within each sample, then combines *p* values across samples using Fisher’s method to call peaks. We compared this Genrich pipeline to strategies which called peaks in individual samples followed by merging (Additional file 2: Fig. S3). The Genrich strategy produced peaks that are likely derived from nucleosome-free and mono-nucleosome fragments, as seen by enrichment around 100 bp and 200 bp in the observed peak length distribution (Fig. 3A).

Across 10,293 samples, we identified 1,659,379 autosomal peaks with a median peak length of 250 base pairs, covering approximately 27% of the genome (Fig. 3A). Chromatin accessibility is influenced by a variety of regulatory processes [38–40], such as active binding of transcription factors, and we would expect to see chromatin accessibility peaks in regions associated with gene regulation. To verify the quality of our ATAC-seq peaks, we annotated our peaks, along with length-matched, randomly selected controls, with various genomic features that included transcript annotations and enhancer annotations as defined by the FANTOM5 enhancer atlas [41, 42] (Methods). We found that relative to controls, our ATAC-seq peaks were enriched for genomic regions annotated as enhancers and all transcript annotations but depleted for gene intergenic regions (Additional file 2: Fig. S4, Additional file 1: Table S3). Similarly, we would expect our ATAC-seq peaks to be enriched for histone modifications associated with gene regulatory regions [43–45]. The NIH Roadmap Epigenomics Mapping Consortium [46] provides chromatin immunoprecipitation with sequencing (ChIP-seq) data representing eight different histone marks from 556 cell line, tissue, and primary cell samples derived from a variety of biological origins. Using these data, the highest enrichment of our ATAC-seq peaks and chromatin histone marks was for H3K4me1, a histone mark that has been linked to enhancers (Additional file 1: Table S4) [43]. In contrast, our ATAC-seq peaks were depleted for overlap with the histone mark H3K9me3, which is associated with gene repression and heterochromatin [47]. Together, these data suggest that our ATAC-seq peaks are enriched for cis-regulatory regions, as expected for genomic sequences implicated in regulatory activity and indicating high quality peak calls.

### Inferred genotypes support high-powered caQTL mapping across samples

Next, we sought to identify genetic variants that are associated with differences in measured chromatin accessibility in ATAC-seq peaks, i.e., caQTLs. We tested a 10 kilobase (kb) window in *cis* flanking each chromatin accessibility peak, as we anticipate that genetically altered active transcription factor binding sites are likely to be found within or very nearby regions of chromatin accessibility due to the causal effect of TF binding on chromatin accessibility [48, 49]. Utilizing our peak calling and genotyping pipelines, we identified 24,159 chromatin accessibility peaks with a significant caQTL at FDR 5% across 1454 unique donor samples (Fig. 3B, Methods, Additional file 1: Tables S5–S6). To mitigate potential confounding from population stratification, we estimated variation in similarity across donors generated by our genotyping via principal components analysis (PCA), including 3 PCs as covariates in discovery analysis. In addition, we also included 200 PCs generated from the donor chromatin accessibility peak read count matrix to mitigate potential latent confounders in QTL mapping [50] (Methods).

To ensure that caQTL mapping results were not being significantly impacted by certain sample characteristics, such as cell/tissue type or whether samples were cancer-derived, we separately performed caQTL mapping in various subsets of samples to address these concerns. We performed caQTL mapping separately in cancer-derived samples (*n* = 312) and non-cancer samples (*n* = 1132) and found that ~93% of the caQTL peaks found in the cancer samples analysis and ~67% of the caQTL peaks found in the non-cancer samples analysis were found in the global analysis (π_1_ values 0.63–0.99) (Additional file 2: Figs. S5–S6). We assessed the impact of sample cell/tissue type in two different ways. First, we chose two groups of samples that were well represented in our dataset based on our annotations and groupings, T cells (*n* = 210) and brain (*n* = 178). We found that ~39% of the caQTL peaks found in the brain samples and ~65% of the caQTL peaks found in the T cell samples were found in the global analysis (π_1_ values 0.51–0.85) (Additional file 2: Figs. S7–S8). Additionally, we performed caQTL mapping with all samples by including a cell type covariate based on annotated cell/tissue type identity and found that ~87% (π_1_ value = 0.99) of caQTL peaks were rediscovered by including cell type as a covariate (Additional file 2: Fig. S9). These caQTL mapping results suggest that our global analysis had the greatest caQTL discovery power and was not significantly affected by various sample characteristics.

We examined the quality of our caQTL variants by determining whether they were enriched for expected functional characteristics. First, we confirmed that the distribution of positions for lead caQTL variants was centered within the open chromatin peak tested, as expected (Fig. 3C). In addition, we observed that peaks with a mapped caQTL were the most strongly enriched for gene 5′ UTRs and enhancer regions while depleted in gene intergenic regions (Additional file 2: Fig. S10, Additional file 1: Table S7). Interestingly, caQTL peaks were further enriched in enhancer regions compared to all chromatin accessibility peaks, suggesting that caQTLs we mapped may be found at genomic elements involved in distal gene regulation. This could potentially arise due to selective pressure reducing functional variation in promoters and other proximal elements.

Additionally, we examined whether our caQTL peaks were enriched for transcription factor binding sites in the ENCODE transcription factor ChIP-seq data from 129 cell types and 340 transcription factors [51]. As expected, caQTL peaks, compared to matched random controls (Methods), were significantly enriched for binding sites for many transcription factors (Additional file 1: Table S8). Enrichment of these functional characteristics supports the conclusion that our caQTLs are high quality, reflect enrichment in expected regulatory elements, and can help identify genetic mechanisms relevant to regulation of gene expression. We sought further evidence that caQTL variants were enriched for functional roles in gene expression regulation by intersecting them with eQTLs. Across all 49 Genotype-Tissue Expression (GTEx v8) tissues, we observed caQTL/eQTL enrichments ranging from 1.96 to 4.75-fold per tissue and a total of 2972 (~13% of unique caQTLs) unique overlapping lead caQTL/lead eQTL variants found across all tissues, for an enrichment of approximately 1.73-fold (Additional file 1: Table S9).

Finally, to further demonstrate that our catalog represents reproducible peaks and caQTLs, we compared our findings here to a recent caQTL study that identified variants associated with chromatin accessibility in African LCL samples [37] not included in our discovery effort. Lead caQTLs and peaks identified in our study resulted in a replication rate (π_1_ value [52, 53]) of 0.62 with this orthogonal study (Fig. 3D). Additional replication analyses were performed for another molecular QTL, histone QTLs (hQTLs), from a study that measured levels of H3K27ac and H3K4me1 in LCLs and identified 6261 enhancer hQTLs [54]. We identified a modest number of overlapping lead caQTLs/hQTLs that were enriched ~ 5.96-fold over hQTL lead variant overlap with caQTL-matched background variants. Effect direction of overlapping caQTLs/hQTLs was largely concordant and caQTL summary statistics for lead hQTLs were enriched for low *p* values, suggesting that both studies are capturing common signals, although power differences may lead to differences in discoveries (Additional file 2: Fig. S11). Together, these analyses further demonstrate that on average, our catalog of caQTLs is high quality and provides insight into how genetic variation may affect gene regulation and complex traits.

### Colocalization suggests shared causality between chromatin accessibility, complex traits and expression QTLs

To gain further insight into the molecular mechanisms underlying GWAS signals, we sought to link GWAS association signals, expression QTLs (eQTLs), and caQTLs together via statistical colocalization (Methods). Colocalization analysis discerns if an association signal is likely shared between two traits, suggestive of a common underlying genetic mechanism. First, we examined which caQTL signals are shared with GWAS signals across a variety of complex human traits. We obtained GWAS summary statistics from a subset of the UK Biobank (UKBB) study, selecting 78 traits of interest with high confidence of significant heritability (Methods) [55]. We then performed colocalization analysis (Methods) for any caQTL peak that was located within 1 Mb of a genome-wide significant lead GWAS signal (Methods). We observed that 69 traits had a caQTL/GWAS colocalization event (PP3 + PP4 > 0.8 and PP4/(PP3 + PP4) > 0.9.) for a total of 13,735 colocalization events across all traits, involving 4735 (~ 20%) unique caQTL peaks and 5197 (~ 37%) unique tested GWAS signals (Additional file 1: Table S10).

Regulatory variants do not always affect the nearest gene and assigning a GWAS signal to a causal gene is not a trivial procedure [56, 57]. Furthermore, comparison of the overlap between lead variants of GWAS signals and the lead variant of eQTLs can suggest the incorrect causal gene [58]. Given the prominence of long-range gene expression regulation, colocalization of cis regulatory elements with eGenes can suggest a shared causal variant [59, 60]. We performed colocalization analyses between caQTLs and eQTLs from 49 tissues obtained from GTEx v8. Across all tissues, between 385 (kidney) and 5856 (thyroid) eGenes colocalized with our caQTLs and showed consistent directionality across shared lead variants that colocalized (Additional file 2: Fig. S12). Colocalized caQTLs/eQTLs were shared across a median of three tissues and a mean of eight tissues, while 18,826 unique eGenes colocalized with caQTLs in any GTEx tissue (Additional file 2: Fig. S13, Additional file 1: Table S11). We found that only 14% of eQTL/caQTL colocalizations involve the gene nearest to the lead caQTL and that there was a median of 5 genes closer to the lead caQTL than the colocalizing gene (Additional file 2: Fig. S13), highlighting the role of caQTLs in distal gene regulation. Additionally, the putative regulated gene transcription start site (TSS) was a median of 76,129 base pairs away from the colocalizing caQTL (Additional file 2: Fig. S13). These results suggest that caQTLs may often be found tagging and potentially modifying the behavior of distal gene regulatory elements and highlight the complexities of gene regulation.

### Multiple molecular QTL datasets provide insight into regulatory mechanisms underlying GWAS associations

**Fig. 4:**
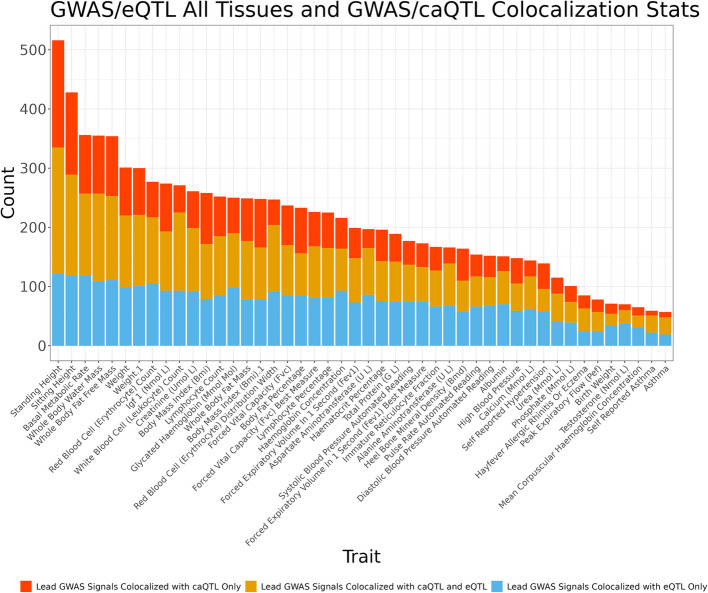
caQTLs map to regions tagged by GWAS and eQTL variation. For each GWAS trait, independent lead GWAS variant signals were checked for colocalization with caQTL and eQTL signals across all GTEx tissues. Plotted is the number of unique lead GWAS signals per colocalization group, as multiple caQTL peaks, eGenes, etc. can colocalize with the same lead GWAS signal. Traits with greater than 50 colocalizing lead variants shown

eQTLs have been shown to provide a regulatory mechanistic hypothesis for GWAS associated signals, yet only an estimated ~25–43% of GWAS signals colocalize with known eQTLs [6, 61], implying that more than half of GWAS loci may lack an obvious functional, mechanistic hypothesis [6, 62–64]. caQTL mapping could help close that gap if, for example, the effects of the eQTL are only apparent in certain cellular contexts, during specific developmental stages, or in the presence of external stimuli [65–67], whereas chromatin accessibility may be primed and reveal effects in a different context [68]. caQTL mapping could also provide mechanistic explanations for GWAS loci in situations where multiple independent eQTLs may complicate colocalization analyses [69]. Across all traits and GTEx tissues, we find that lead GWAS signals colocalize with a median of 6 eQTLs and 2 caQTLs (Additional file 2: Fig. S14). For each GWAS trait, we then considered whether independent GWAS lead signals colocalize only with eQTLs, colocalize with both caQTLs and eQTLs, or colocalize only with caQTLs. Across all GWAS, a median of 34 unique signals colocalized with a caQTL only, a median of 70 unique signals colocalized with an eQTL only, and a median of 56 unique signals colocalized with both a caQTL and an eQTL (Fig. 4, Additional file 1: Table S13). To gain a better understanding of what is gained by caQTLs compared to eQTLs when colocalizing with GWAS signals, we analyzed the colocalization posterior probabilities for loci where GWAS signals colocalized only with caQTLs. At many of these loci, we found that eQTL/GWAS colocalization posterior probabilities suggested that independent variants were causal for the two traits (COLOC PP3), or that an association was only found with one trait (COLOC PP1) (Additional file 2: Figs. S15–S16). These differences may reflect context-specific behavior of gene regulation that is not well captured by steady-state, adult gene expression data, but may still be reflected in chromatin accessibility. Another possibility is that eQTL studies, which in GTEx ranged from 73 to 706 samples, are underpowered to detect variants that are causal for GWAS signals. In this case, we expect that more GWAS signals would colocalize with both caQTLs and eQTLs, and fewer would colocalize only with caQTLs. Overall, these results demonstrate that incorporating both caQTLs and eQTLs nominates putative causal mechanisms for approximately 28% more GWAS signals than using eQTLs alone. Furthermore, 59% of GWAS signals we tested were linked with either a caQTL, eQTL, or both (Additional file 2: Fig. S17). Instances where GWAS signals colocalized with both caQTLs and eQTLs may also allow for a better delineation of the mechanism at these loci by nominating a candidate caQTL-associated gene regulatory element to a target eGene [70].

To gain insight into molecular mechanisms that may be unique to caQTLs as compared to eQTLs, we calculated the enrichment of colocalizing caQTLs and lead eQTLs for diverse genomic annotations. caQTLs and eQTLs involved in colocalizations with GWAS signals were both significantly enriched for all tested genomic annotation categories except for intergenic regions, where they were significantly depleted, compared to length, GC, repeat matched random controls (Additional file 2: Fig. S18, Methods). However, caQTLs from GWAS/caQTL and caQTL/GWAS/eQTL colocalization events were further enriched for enhancer regions and slightly less depleted in intergenic regions than eQTLs from GWAS/eQTL colocalizations alone (Additional file 2: Figs. S19–S20, Additional file 1: Tables S14–S16). In contrast, lead variants of eQTLs that colocalized with a GWAS were less enriched in enhancer regions and showed slightly greater depletion for intergenic regions consistent with previous reports (Additional file 2: Fig. S21) [6, 71]. In contrast, eQTL regions were further enriched for gene 3′ UTRs when compared to regions involved in colocalizations involving caQTL regions. These differences in enrichment may be due to systematic differences in GWAS signals that are explained by eQTLs compared to those explained by potentially distal regulatory mechanisms captured by caQTLs, whose regulatory effects may be less dependent on distance to gene TSS [72, 73].

While our caQTLs were called from heterogeneous cell/tissue samples, they are enriched for brain and whole blood samples (Fig. 1). To reflect this, we also performed an analysis of caQTL/GWAS colocalizations compared to eQTL/GWAS colocalizations from brain cortex and whole blood only. Across 70 GWAS, each trait has at least one GWAS signal that colocalized only with a caQTL, and one trait, standing height, had 371 lead GWAS signals that colocalized exclusively with caQTLs compared to brain eQTLs. In contrast, we identified a maximum of 43 lead GWAS variants that colocalize only with eQTLs for a given trait. Across all GWAS, a median of 78 unique signals colocalized with a caQTL only, a median of 11 unique signals colocalized with an eQTL only in whole blood, and a median of 14 unique signals colocalized with both a caQTL and a whole blood eQTL (Additional file 2: Fig. S22, Additional file 1: Table S17). Furthermore, across all GWAS, a median of 90 unique signals colocalized with a caQTL only, a median of 7 unique signals colocalized with an eQTL only in brain cortex, and a median of 8 unique signals colocalized with both a caQTL and a brain cortex eQTL (Additional file 2: Fig. S23, Additional file 1: Table S18). Compared to the analysis considering eQTLs across all tissues, we find that caQTL/GWAS only colocalizations occur with a larger proportion of GWAS signals in single tissue eQTL analysis colocalizations. This discrepancy provides further evidence that using caQTLs can provide molecular insight into GWAS association signals beyond eQTLs when restricting to a single eQTL tissue.

### Integration of caQTLs informs mechanistic interpretation at many GWAS loci

Colocalization analysis with QTL datasets across multiple modalities, such as expression and chromatin accessibility, has previously been shown to nominate putative target genes underlying more GWAS signals than a single modality alone [70, 74]. We identified signals that colocalized separately with both caQTLs and eQTLs and quantified how many of the GWAS-colocalizing caQTLs and eQTLs also colocalized with each other. We identified 53,223 unique colocalization events involving a GWAS trait, caQTL peak, and eGene identified in a GTEx tissue (Additional file 1: Table S19). These were comprised of 2564 unique eGenes and 1894 unique caQTL peaks.

In cases where caQTLs colocalize with both GWAS signals and eQTLs, they provide a more complete picture of the mechanisms likely driving the association signal. First, we provide an instructive example of a well-characterized GWAS locus strongly associated with plasma low-density lipoprotein cholesterol (LDL-C) at the 1p13 locus. eQTL colocalization analyses at this locus, followed by functional characterization in vitro and in vivo, suggest that the causal gene at this locus is *SORT1*, with expression differences observed in the liver [49]. We find a caQTL at this locus that colocalizes with both the *SORT1* eQTL in liver, and the GWAS trait self-reported high cholesterol (Additional file 2: Fig. S24). This caQTL peak contains a well-studied noncoding variant that creates a *C/EBP* (CCAAT/enhancer binding protein) TF binding site, altering hepatic expression of *SORT1* and plasma LDL-C levels [49]. This highlights the ability of our analyses to identify verified mechanisms underlying GWAS signals.

**Fig. 5:**
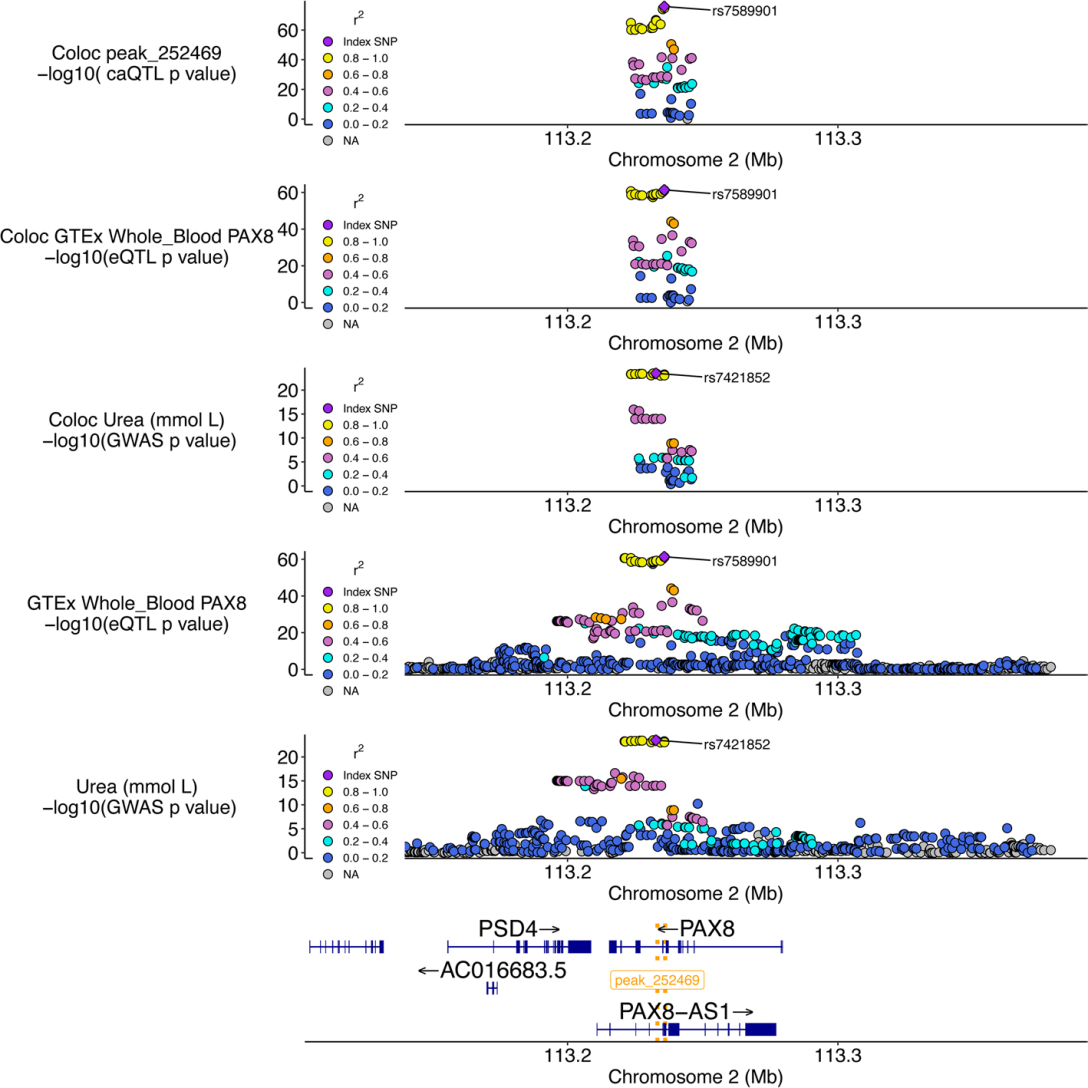
Change in chromatin accessibility and expression implicate *PAX8* in serum urea levels. The top three plots are the colocalization windows (10 kb + caQTL peak) for the caQTL, eQTL, and GWAS, respectively. The following two plots are showing a larger window to illustrate the eQTL and GWAS signals, respectively, at this locus at a different scale. The bottom gene track highlights the position of genes at this locus, as well as the location of the caQTL peak (gold dotted lines)

In a second example, we identified a compelling locus where a caQTL peak, a whole blood eQTL for *PAX8*, and a GWAS signal for blood urea levels colocalized (Fig. 5). The shared lead caQTL and eQTL variant, rs7589901, is an intronic variant within the *PAX8* gene. The reference allele of rs7589901-A is associated with increased chromatin accessibility in the associated peak when considering the aggregated signal across all samples (Additional file 2: Fig. S25). This relationship is also seen across many cell/tissue-types groups based on our sample annotations, suggesting that this caQTL effect is mostly shared across many of the well-powered cell type groups (Additional file 2: Fig. S26). Based on motif analysis, several TFs are predicted to bind to a motif at this locus. One such TF, *ZNF135*, is predicted to bind to a motif overlapping rs7589901, with the alternate C allele strongly favored for binding based on the position weight matrix (PWM), a model that reflects the relative frequency of the base occurring for that position in the motif [75] (PWM value: C allele = 0.81, Additional file 2: Fig. S27). In GTEx, the rs7589901 eQTL direction of effect is concordant with the caQTL direction of effect, suggesting that increased accessibility at this locus is associated with increased *PAX8* gene expression in whole blood. The lead GWAS variant at this locus, rs7421852, is associated with increased blood urea levels, is ~3000 bp from rs7589901, and is in strong LD (*r*^2^ = 0.85) with rs7589901 in our caQTL sample genotypes. These results suggest a potential mechanism where *ZNF135* is acting as a transcriptional repressor at this locus, a functional role that has been implicated in a different context [76]. The culmination of evidence suggests a mechanism where decreased *ZNF135* binding leads to increased chromatin accessibility, increased expression of the *PAX8* gene, which has been linked to urea regulation [77], and lower blood urea levels (Additional file 2: Fig. S28). Importantly, at any locus where colocalizations nominate eGenes and/or variants are predicted to affect TF binding, functional experiments are needed to validate proposed mechanisms. Such examples, however, demonstrate the power of integrating multiple molecular QTL datasets to nominate mechanistic hypotheses that may be further validated experimentally.

### Sample heterogeneity enables identification of context-specific clusters

**Fig. 6:**
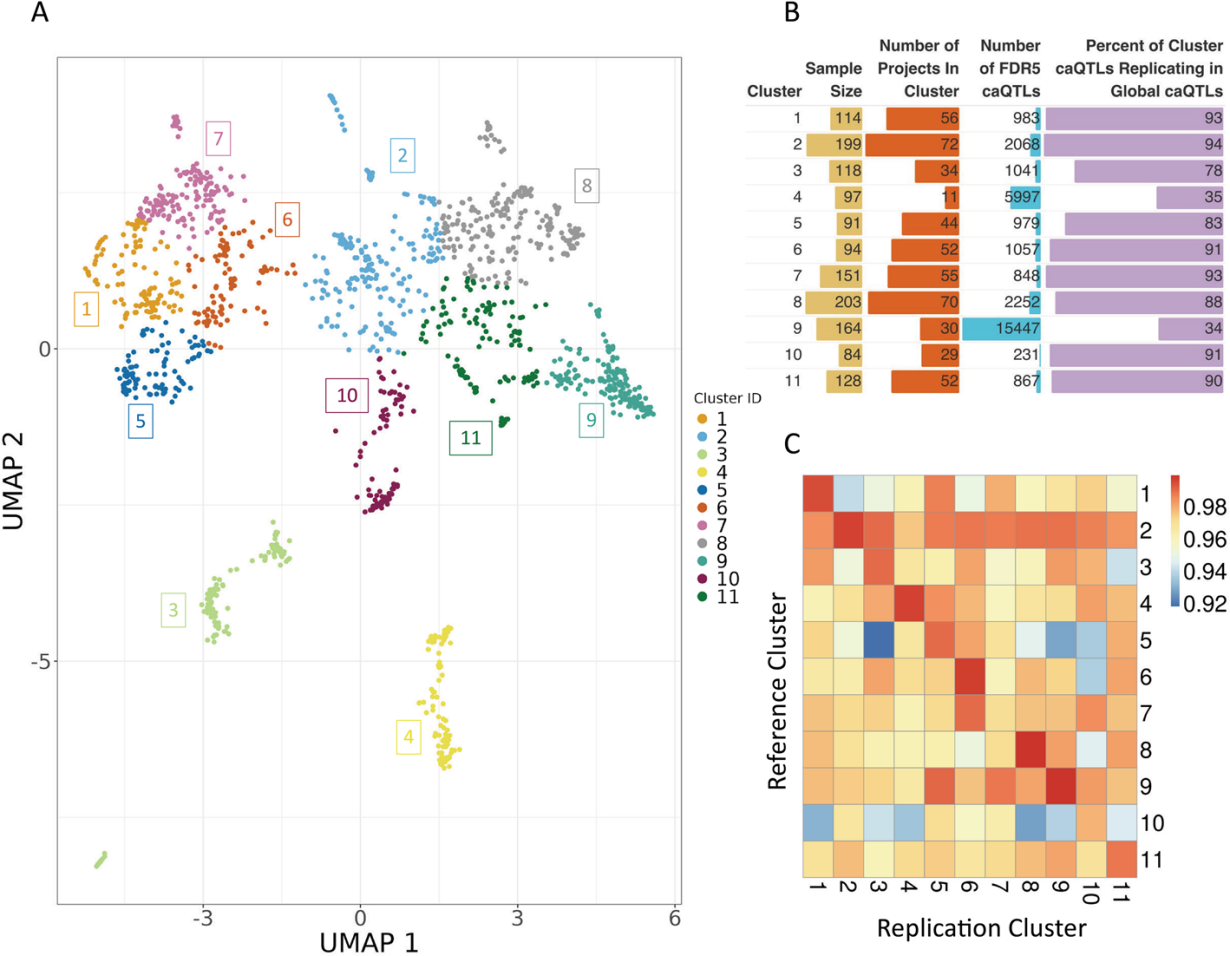
Clustering and discovery of cluster caQTLs across ATAC-seq samples. **A** UMAP followed by *k*-means clustering to identify groups of related samples based on chromatin accessibility profiles across all peaks. **B** Cluster characteristics, caQTLs identified, and replication with respect to global caQTL mapping. The size of each bar represents the magnitude of each category within each cluster. **C** Replication rate (π_1_ value) of caQTLs identified in each cluster compared to those found in all other clusters

Because profiles of chromatin accessibility often segregate context or cell-type specific information, we next grouped our samples by their profiles of chromatin [78]. We performed dimensionality reduction [79] and applied an unsupervised clustering method [80] to identify groups of similar samples, identifying 11 clusters (Fig. 6A, Methods). After clustering, we used sample metadata to assign a label to each cluster, denoting the presumed biological origin. Although many of the cell/tissue type labels were generated and harmonized by our review of each project and incorrect assignments are possible where metadata was lacking, overall, clustering appears to be mainly driven by the tissue or cell type from which the sample is derived (Additional file 2: Figs. S29–S30). For example, blood cell types appear to be grouped together or near each other in separate, but related clusters. In addition, we found other examples of clusters where nearly half of the samples are derived from a single tissue, such as pancreas. Annotating samples with other aspects of metadata, such as primary sample vs. cell line, or cancer vs. non-cancer samples, did not appear to explain clustering results (Additional file 2: Fig. S31).

### Clustering allows for identification of caQTLs in specific clusters

To determine whether clustering samples of similar biological origin enables the discovery of additional caQTL signals, we next performed caQTL mapping within each cluster. Each cluster is composed of a different number of samples, with varying contributions from cell types and projects, which is reflected in the number of caQTLs identified in each cluster. Cluster sample size ranged from 84 to 203 unique donor samples (Additional file 1: Table S20) and resulted in 231–15,447 (FDR < 5%) caQTLs identified in a single cluster. As in the global analysis, cluster caQTLs showed similar patterns of genomic region annotation enrichments (Additional file 2: Fig. S32) and lead caQTLs were centered within the open chromatin peak tested (Additional file 2: Fig. S33). Across all clusters, cluster caQTLs rediscovered 34–94% of caQTL peaks observed in the global analysis (Fig. 6B) with median global caQTL replication rate of 0.99 (π_1_ value) across all clusters (Additional file 2: Fig. S34). Analysis comparing cluster caQTL peak discoveries to other clusters resulted in a range of caQTL peak rediscovery (Additional file 2: Fig. S35) but high replication rate across clusters (π_1_ value 0.92–0.99) (Fig. 6C, Additional file 1: Table S21). This suggests that clusters are capturing common global signals, but some clusters are better powered at identifying caQTLs that might be cell/tissue-specific. For example, cluster 9, which identified the largest number of cluster caQTLs, is comprised of more than 50% LCL samples, many of which are from a single study. Approximately 2/3 of the caQTL peaks identified in cluster 9 are not identified as caQTL peaks in the global analysis performed across all tissues/cell types, suggesting that cluster 9 may be better powered to discover caQTLs more prevalent in LCLs and related blood cell samples. As a measure of reproducibility across experiments, we found that cluster 9 caQTL lead variants were enriched for evidence of caQTL peak causality in the original study [35] that the majority of cluster 9 samples originate from (Additional file 2: Fig. S36). These results suggest that, as with eQTLs, future work increasing the sample size to examine cell/tissue-specific caQTLs is likely to capture novel caQTLs that will be useful for elucidating molecular mechanisms underlying GWAS signals.

Mapping caQTLs in clusters highlights the increase in caQTL discovery power of aggregating all samples across experiments, particularly for caQTLs that might be found across cell types. In our global analysis, we identified 24,159 caQTL peaks, with a maximum of 5315 of those also identified in a single cluster caQTL mapping experiment. This suggests that by considering all samples, we achieve greater than a 4.5-fold increase in caQTL discovery power for global caQTLs. Across all clusters, we identify 8643 (36% of global) caQTL peaks that were also found in the global analysis and 15,029 caQTL peaks that were not found in the global analysis.

### Cluster-specific caQTLs can explain additional gene regulation and GWAS signal causality

We next performed colocalization analysis between GTEx eQTLs and the caQTLs identified within each cluster to determine if cluster-specific caQTLs appear to be involved in gene regulation as well. As in the cluster caQTL analysis, we find that the number of colocalizations found per cluster was commensurate with the number of caQTLs identified in each cluster. We find a maximum of 13,989 unique eGenes colocalizing in a single cluster, and a total of 17,187 unique eGenes colocalize when considering all clusters (Additional file 1: Tables S22–S23). Compared to the global analysis, which identified a total of 18,826 unique colocalizing eGenes, 14,459 were also colocalized in the cluster analyses, suggesting that the majority of colocalizing eGenes are identified across both analyses. As in the cluster caQTL analyses, we find that colocalizing eGenes are often shared across clusters (Additional file 2: Fig. S37). Considering all cluster colocalization events, 7940 total eGenes were found to uniquely colocalize in a single cluster, with 5653 (71%) of these in cluster 9. Overall, we find a variable number of cluster-specific caQTL/eQTL colocalizations per cluster, many of which are shared across clusters.

Our previous analyses assessed the benefit of utilizing global caQTLs in GWAS colocalizations compared to eQTLs. In this analysis, we considered eQTLs that were discovered in experiments performed in single tissues, experiments that are much more likely to identify variants with tissue-specific effects compared to our multi-tissue, global caQTL mapping strategy. Cluster-specific caQTLs might more closely mimic these single-tissue eQTL datasets, as these caQTLs were mapped in clusters of samples that likely shared a similar biological origin. To better compare the contribution of eQTLs and caQTLs to GWAS signals, we considered caQTLs identified in both global and cluster-specific analyses to assess colocalization improvement. Across all GWAS traits and eQTL tissues tested, we find that combining global and cluster-specific caQTLs results in an increase of the contribution of caQTLs to GWAS colocalizations. Specifically, we find a median of 44 GWAS signals colocalizing with caQTLs only and a median of 76 GWAS signals colocalizing with both caQTLs and eQTLs (Additional file 2: Fig. S38, Additional file 1: Table S24). Both measurements are increases compared to the global analysis only. In contrast, the median number of GWAS signals that colocalize with eQTLs only decreased to 48 (Additional file 2: Fig. S38, Additional file 1: Table S24). Leveraging both global and cluster caQTLs, together with eQTLs, we explained a median of 64% of GWAS signals tested (Additional file 2: Fig. S39). Overall, we find that both global and cluster-specific caQTLs can contribute to the causal mechanisms underlying GWAS signals not captured by eQTLs.

## Discussion

We developed a pipeline to discover caQTLs on a large scale by aggregating and genotyping large-scale ATAC-seq data across many studies. We collected 10,293 human ATAC-seq samples, representing 1454 unique donors, from public databases that come from a diversity of cell types and conditions, demonstrating that genotype data can be accurately called from ATAC-seq data, and identified unique sample donors, both within and across projects. Combining accessibility and genotype information, we performed caQTL analysis and were able to capture global and cluster-specific caQTLs. caQTL studies are often limited by sample size constraints. We show that amassing public-domain project data allows for identification of a greater number of caQTLs than smaller individual studies alone. We demonstrated that caQTLs are enriched for various regulatory elements and likely underlie gene expression differences and complex human traits. We provide our large catalog of global and cluster caQTLs as a resource.

Our study does have limitations and opportunities for further development. Naturally, as more ATAC-seq data are generated, a similar study could be repeated on a larger scale. Additionally, the clustering performed in our study was coarse and may have grouped multiple cell types or contexts together. With a larger sample size from new studies or more extensive exploration of clustering methods or cell type prediction approaches, these grouping could be further refined and made more homogeneous, which would be expected to boost statistical power for discovery. Although we analyzed a large and diverse set of samples and experiments, many GWAS signals were not tagged by one of our caQTLs (and/or by eQTLs). One explanation for this is that we are missing many cluster/context-specific caQTLs that may underlie the remaining GWAS signals. Another limitation of this study is that while the sample contexts were diverse, we still do not have sufficient sample size across some disease-relevant contexts to fully examine context-specific caQTLs. Further work, perhaps using single cell ATAC-seq data, is necessary to gain insight into tissue/cell context specific caQTLs. Other types of molecular QTLs may underlie some unexplained GWAS signals [63]. Incorporating additional data modalities, such as those reflecting chromosome conformation changes, may identify additional QTLs underlying GWAS loci. A recent study has shown that genetic variants in enhancer regions affect gene expression changes via enhancer-promoter touching and looping processes [81]. Integrating HiC or HiChIP datasets with ATAC-seq data can provide insight into this process. These datasets may also help identify target genes or resolve situations where multiple eGenes are implicated as causal genes at a locus [82]. Furthermore, other mechanisms, such as DNA methylation (meQTLs) [83, 84] or post-transcriptional processes such as splicing (sQTLs) [71] or protein concentrations (pQTLs) [85], could underlie GWAS signals that have yet to be explained.

Although we observed colocalization analysis between our caQTLs and GWAS signals on par with previous studies [73], experimental validation is necessary to determine whether putative causal variants underlying these QTLs directly mediate disease risk [86, 87]. Previous studies have shown that this type of analysis has led to the correct identification of molecular mechanisms underlying disease. For example, regulatory mapping has successfully identified gene targets that can be experimentally modulated to produce a phenotypic effect both in vitro and in vivo [88]. Furthermore, caQTL analyses have been used to predict mechanisms underlying GWAS signals with follow-up functional experiment results supporting these predictions [15]. Ultimately, regulatory elements and gene targets that we identify as implicated at GWAS loci will need additional support from low-throughput experimental techniques to confirm our findings, such as using base editing to dissect variant function [89]. Toward the goal of understanding molecular mechanisms underlying GWAS signals, molecular QTLs generate hypotheses and our work has demonstrated that including caQTLs in these experiments increases the number of GWAS signals for which a putative molecular mechanisms may be identified.

## Conclusions

In summary, we have deployed a pipeline to call a set of consensus peaks from thousands of publicly available ATAC-seq samples and genotype these samples directly from the experimental sequencing reads. We leveraged these data to identify caQTLs that likely share causal variants with eQTLs and GWAS signals. We show that caQTLs can improve our understanding of the mechanisms underlying GWAS signals and we provide this dataset as a resource for use in further fine-mapping experiments.

## Methods

### Sample collection

ATAC-seq samples were identified through the Gene Expression Omnibus (GEO) database and downloaded. Collected sample metadata is found in Additional file 1: Table S1.

### Benchmarking on HapMap samples

We downloaded ATAC-seq for 71 HapMap samples from ENA project PRJEB28318 [35]. Cram files were converted to bam files and reads that map to the mitochondrial genome were removed. We aligned the sequencing reads to GRCh38 using bowtie2 and retained only autosomal chromosomes. Duplicated reads tagged by Picard were removed and Base Quality Score Recalibration (BQSR) was performed using GATK tools. Variant calling was done using GATK HaplotypeCaller [34, 90, 91], HaplotypeCaller with imputation, and with Gencove’s low-pass sequencing pipeline. Loci with less than 2 reads were filtered out and variants were mapped to GRCh37 using Picard LiftoverVcf. Minimac4 was utilized to run imputation with reference panel derived 1000G Phase 3 (https://csg.sph.umich.edu/abecasis/mach/download/1000G.Phase3.v5.html). We kept only the genotype for common variants derived from 1000G with MAF > 0.05. The gold standard variants were obtained from https://www.internationalgenome.org/data-portal/data-collection/grch38 [92]. At loci with discordant genotype calls between GATK genotype caller and imputation, we used custom machine learning methods for combining the GATK and imputation results. With these models, 80% of the samples were utilized for training and the remaining 20% were utilized for testing. During training, each model is trained to use genotypes from the GATK genotype caller and imputation to predict the gold standard genotypes obtained through WGS. The training script is found in train_genotype_predictor.py. We then compared the called genotype dosage to the gold standard genotype by computing the Spearman correlation and mean squared error (MSE).

### Benchmarking for caQTLs in HapMap samples

We first obtained caQTLs using ATAC-seq reads with Benjamani-Hochberg (BH) corrected *p* value < 0.05, then ran QTL analysis using gold standard genotype and obtained caQTLs with BH corrected *p* value < 0.05. The precision is computed as the percentage of replicated caQTLs at FDR < 0.05 using the gold standard genotype. Similarly, we first obtained caQTLs using gold standard genotypes with BH corrected *p* value < 0.05, then ran QTL analysis using ATAC-seq reads and obtained caQTLs with BH corrected *p* value < 0.05. The recall is computed as the percentage of replicated caQTLs at FDR < 0.05 using the ATAC-seq reads.

### Peak calling

Genrich [28] (v0.6.1) was used to call peaks. A slightly modified version of Genrich was applied to allow peak calling across a large number of samples (https://github.com/maxdudek/Genrich). Genrich assigns *p* values to genomic positions within each sample followed by combining *p* values across samples using Fisher’s method to call peaks. Bam files were filtered using “samtools view -S -b -q 10.” Bam files were name sorted using “samtools sort -n /path/to/q10_filtered_bams/sample.bam | samtools view -h -o /path/to/nameSortedBams/sample.bam.” Peak calling parameters were “Genrich -t /path/to/nameSortedBams/sample1.bam, path/to/nameSortedBams/sample2.bam, path/to/nameSortedBams/sampleN.bam, -j -o /path/to/outputFile -v -E /path/to/blacklistRegions.bed -r -q 0.05-y.”

### Genomic annotation enrichment

Genomic annotation enrichment analyses were performed using the R package annotatr (v.1.28.0) (https://bioconductor.org/packages/release/bioc/html/annotatr.html). One hundred iterations of random, matched background data using bedtools shuffle with flags “-chrom -excl /path/to/blacklistRegions.bed -g /path/to/chrSizes.txt.” caQTL peak background regions matched on length, GC content, and repeats were generated using R package gkmSVM v.0.83.0 function genNullSeqs with parameters “repeat_match_tol = 0.1,GC_match_tol = 0.1,batchsize = 7000,length_match_tol = 0.05.” Annotations were made with annotatr function annotate_regions with parameters “ignore.strand = TRUE, quiet = FALSE,minoverlap = 200.” *p* values were calculated by quantifying the number of random data iterations that were more extreme than the true data values for each category.

### NIH roadmap enrichment

Histone ChIP-seq data derived from adult human samples were downloaded from https://www.encodeproject.org/search/?type=Experiment&status=released&award.project=Roadmap. ATAC-seq peaks that overlapped histone mark data were identified using bedtools intersect -wo -a /path/to/encodeData.bed -b /path/to/peakCoords.txt. One hundred iterations of random, matched background data using bedtools shuffle with flags “-chrom -excl /path/to/blacklistRegions.bed -g /path/to/chrSizes.txt.” *p* values were calculated by quantifying the number of random data iterations that were more extreme than the true data values for each histone mark.

### caQTL mapping

Sample peak counts were generated for all samples used for peak calling. To remove potential outlier peak regions, peaks with mean count < 1 and max count > 100,000 were removed. Peaks were also removed if > 5000 peak-calling samples had a read count of zero in that peak. Given that a single individual might contribute multiple samples to the 10,293 sample pool, we identified each sample that can be attributed to each individual and averaged sample peak CPM values to calculate a single CPM value per peak for each individual donor. This workflow results in 1454 individual donor samples for caQTL mapping. Code available in file “Post_peakCalling_CountMatrixGeneration_Pipeline.txt.” tensorQTL (v.1.0.9) [93] was used to identify caQTLs using a linear model with 3 genotype PCs, explaining 88% of the variance, and 200 principal components, explaining 51% of the variance, as covariates. The results of ADMIXTURE’s (v.1.3.0) [94] cross-validation procedure suggested that three ancestry populations were represented in our data (Additional file 2: Fig. S40). We compared the results of caQTL mapping with 5 genotype PCs rather than 3 genotype PCs and found 23,699 concordant caQTL peaks (Additional file 2: Fig. S41). PCs generated from the chromatin accessibility peak read count data sample matrix was used to map caQTLs on chromosome 1 over a large range of included PCs. PC correlation with sample metadata was performed using Spearman and Pearson correlation, as well as with Multivariate Analysis Of Variance (MANOVA) (Additional file 2: Fig. S42). The optimized PC covariate number was chosen based on the elbow of the PCs included vs. caQTL discovery plot on chromosome 1 (Additional file 1: Table S25). We tested all genotyped biallelic genetic variants with MAF > 0.05 within 10 kilobases of all open chromatin peak boundaries detected by Genrich from the ATAC-seq data. Empirical *p* values were estimated by tensorQTL to get peak-level *p* values and *q* values [95]. caQTL mapping code available in file “caQTL_mapping_code_pipeline.txt.” All caQTL mapping analyses performed on sample subsets followed the same pipeline described for the global analysis. To assess whether reference allele mapping bias contributes significantly to caQTLs, we ran RASQUAL [10] on FDR5 caQTL peaks identified with tensorQTL and a randomly selected subset of samples (*n* = 48). RASQUAL produces a reference allele mapping bias (Phi) score for each variant tested (Additional file 2: Fig. S43). Considering caQTL lead variants tested with any caQTL peak, 38,660/38,938 (~ 99%) of the Phi estimates were between 0.25 and 0.75 and 33,849/38,938 (~ 87%) of the Phi estimates were between 0.4 and 0.6.

### Lead caQTL/eQTL enrichment

Significant lead eQTL variants were downloaded for 49 tissues from GTEx v8 publicly available data. Unique global sample analysis lead caQTLs (*n* = 21,647) were intersected with lead eQTL variants to assess overlap within each GTEx tissue. The unique intersection of overlaps across all tissues was considered to determine the total number of caQTL lead variants that were found to be a lead eQTL variant in at least one tissue. Background variants were selected to perform enrichment analyses. Background variants were chosen by randomly sampling non-lead caQTL genetic variants that were matched, ± 10%, to the allele frequency and distance to nearest gene transcription start site of true lead caQTL variants. Enrichment of caQTLs/eQTLs in each tissue was calculated as the ratio of the overlap of true lead caQTL/eQTL compared to the overlap of background variants/eQTL across 100 iterations.

### Replication analysis

An external dataset was identified that was not included in our peak calling or caQTL mapping workflow [37]. Global FDR5 caQTL peaks with any overlap with the external study and variants tested in both analyses against these shared peaks were identified. External study *p* values were used for π_1_ replication rate calculation and plotted.

### GWAS trait/signal selection

GWAS summary stats for traits were downloaded February 2021 from the UKBB Neale Lab repository and selected for relevant traits based on the following filters: h2 > 0.05, z > 7, confidence = = high. Independent significant GWAS signals from 78 traits were chosen to prevent counting a single GWAS signal multiple times. This was done by selecting GWAS signals with a minimum *p* value of 5e − 08, considering a window of 50 kb on either side of these variants, clumping all variants with R2 > 0.01, and selecting the variant with the most significant *p* value as the lead GWAS signal for this locus.

### Colocalization analyses

Colocalization was performed using coloc [62] (v.5.2.3). All reported colocalizations utilized a previously published approach to define significance [68]. This approach consists of considering whether the colocalization is sufficiently powered, PP3 + PP4 > 0.8. For those events that surpass this threshold, we assessed whether the colocalization is significant, PP4/(PP3 + PP4) > 0.9. GTEx v8 data were downloaded from https://www.gtexportal.org/home/downloads/adult-gtex/bulk_tissue_expression.

### Colocalization genome annotations

Genomic annotation enrichment analyses were performed using the R package annotatr (v.1.28.0) (https://bioconductor.org/packages/release/bioc/html/annotatr.html). For each type of colocalization, caQTL peaks involved in the colocalization were labeled with genomic annotations they overlap. To perform an enrichment analysis, true data results were compared with the median of 1000 iterations of random genomic regions matched to the true data using bedtools shuffle with flags “-chrom -excl /path/to/blacklistRegions.bed -g /path/to/chrSizes.txt.” Summaries were produced by identifying significant enrichments (annotation category enriched/depleted *p* value ≤ 0.05) across all traits or trait/tissue pairs and calculating the mean and median enrichment/depletion values.

### Clustering analyses

To reduce the dimensions of the data, Uniform Manifold Approximation and Projection (UMAP) was performed on the normalized sample CPM count matrix across all peaks. K-means clustering was performed on UMAP coordinates 1 and 2. Eleven outlier samples were removed from analysis. The number of clusters was optimized using several clustering metrics (Additional file 1: Table S26) and samples were assigned to a cluster based on the results of the clustering algorithm.

### Cluster-specific caQTL mapping

caQTL mapping was performed as in the global analysis. In this analysis, peaks identified in the global analysis were included if at least 50% of cluster samples had non-zero CPMs in that feature, resulting in the removal of 5–5920 (0.0003–0.35% of total peaks). All steps of the caQTL mapping pipeline were performed within each cluster. caQTL mapping was performed including 3 genotype PCs and an optimized number of principal components based on each cluster. For each cluster, a range of PCs generated from each cluster’s chromatin accessibility peak read count data sample matrix was used to map caQTLs on chromosome 1. The optimized PC covariate number was chosen based on the elbow of the PCs included vs. caQTL discovery plot. We tested all genotyped biallelic genetic variants with MAF > 0.05 within 10 kilobases of all open chromatin peak boundaries detected by Genrich from the ATAC-seq data. Empirical *p* values were estimated by tensorQTL to get peak-level *p* values and *q* values [95]. All colocalizations were performed as described for the global analyses.

### Cluster caQTL replication analyses

Cluster caQTL replication of global caQTLs was assessed by extracting global caQTL peak test statistics from each cluster and calculating π_1_ replication rate. The reported replication rate for each cluster was calculated by calculating the median π_1_ replication rate after calculating π_1_ replication rate with a range of values for the lambda parameter (from = 0.1, to = 0.9, by = 0.05). Cluster caQTL replication rate across all other clusters was calculated in a similar fashion. For each cluster, cluster caQTL peak test statistics were extracted from all other clusters and π_1_ replication rate was calculated. The reported replication rate for each cluster was calculated by calculating the median π_1_ replication rate after calculating π_1_ replication rate with a range of values for the lambda parameter (from = 0.1, to = 0.9, by = 0.05).

## Supplementary Information

Additional file 1. Supplementary tables containing results of analyses performed in manuscript.

Additional file 2. Supplementary figures

## Data Availability

The code used to generate the results and figures (under an MIT license), as well as generated data/results are deposited in a Zenodo repository (10.5281/zenodo.12706263) [96]. Publicly available samples used are listed in Additional file 1:Table S1.
