# Supplementary material for: Genotype inference from aggregated chromatin accessibility data reveals genetic regulatory mechanisms
Source: Genome Biol. 2025 Mar 30;26:81. doi: 10.1186/s13059-025-03538-1 (PMC11956263; doi:10.1186/s13059-025-03538-1)
Supplement: Supplementary file 2 — Additional file 2. Supplementary figures [file 13059_2025_3538_MOESM2_ESM.pdf]

Additional file 2

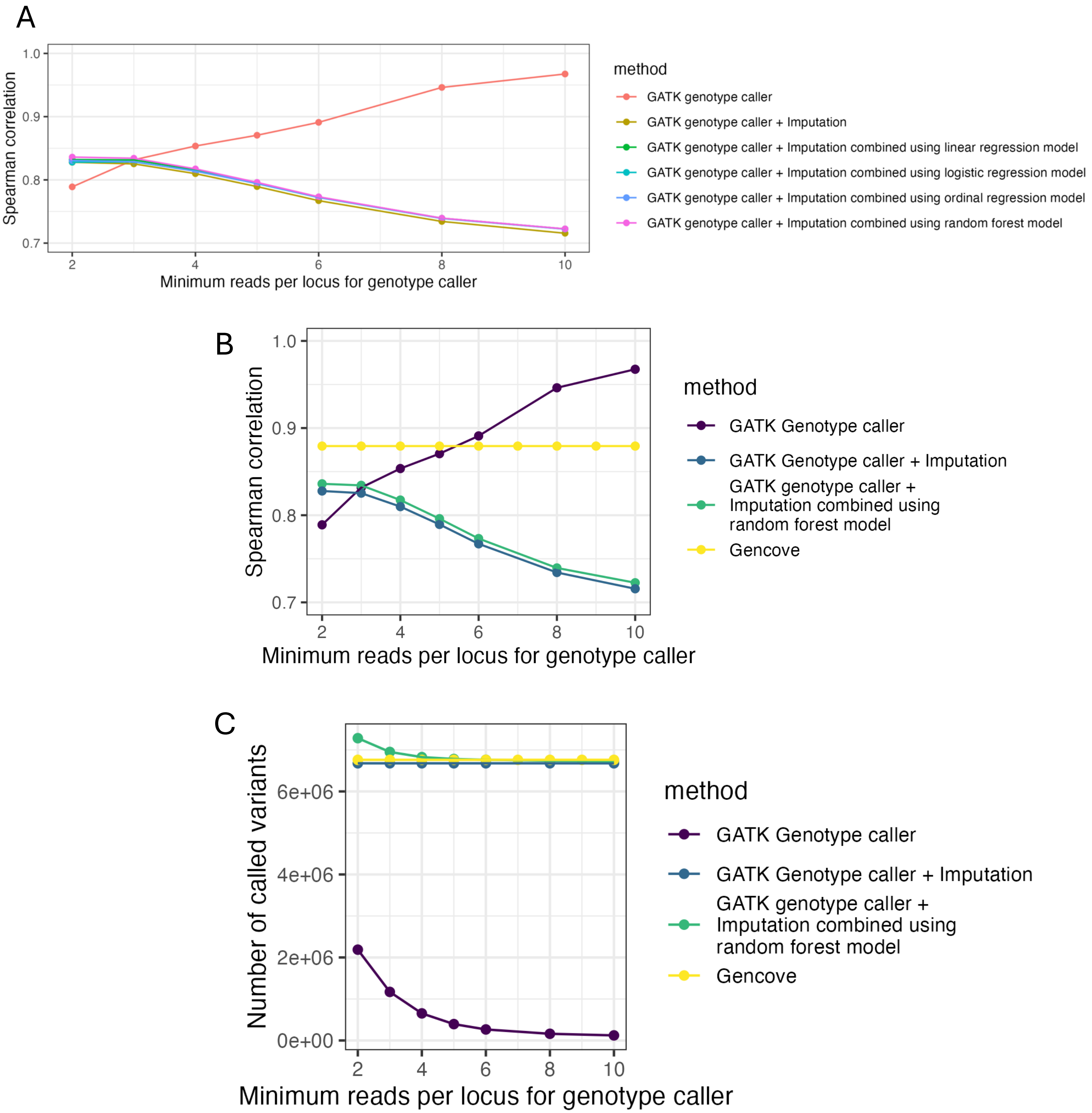

Fig S1: Results of benchmarking genotyping methods. A) Spearman correlation between known and called genotype across methods, including machine learning methods tested to combine loci where GATK genotype caller and imputation genotypes were discordant. B,C) Comparing the accuracy, represented by Spearman correlation between known and called genotype, and number of called variants between the four tested genotyping methods.

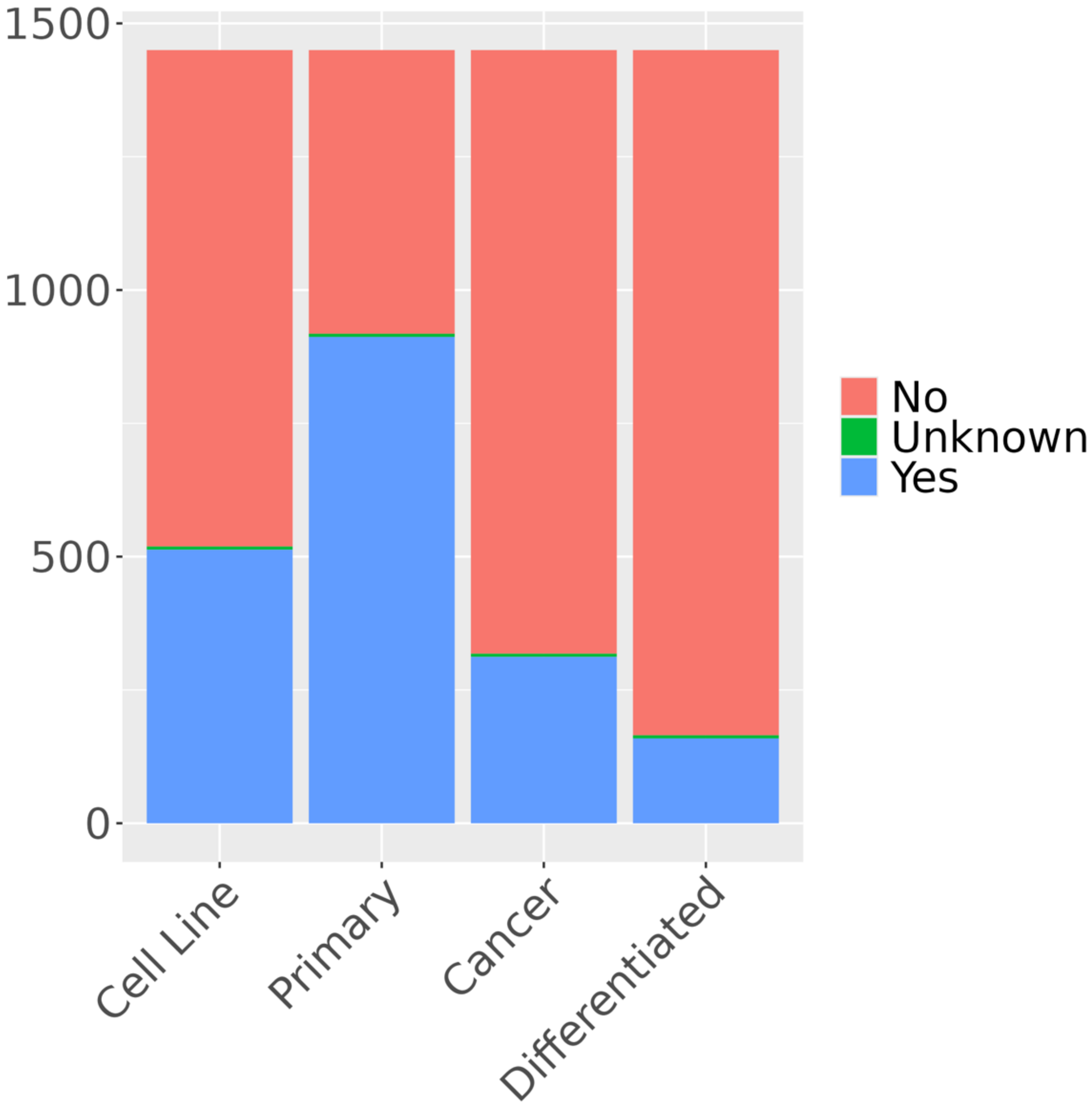

Fig S2: Proportion of samples used for caQTL mapping, based on metadata review, from cell lines, primary tissue, cancer, and experimentally differentiated cell types.

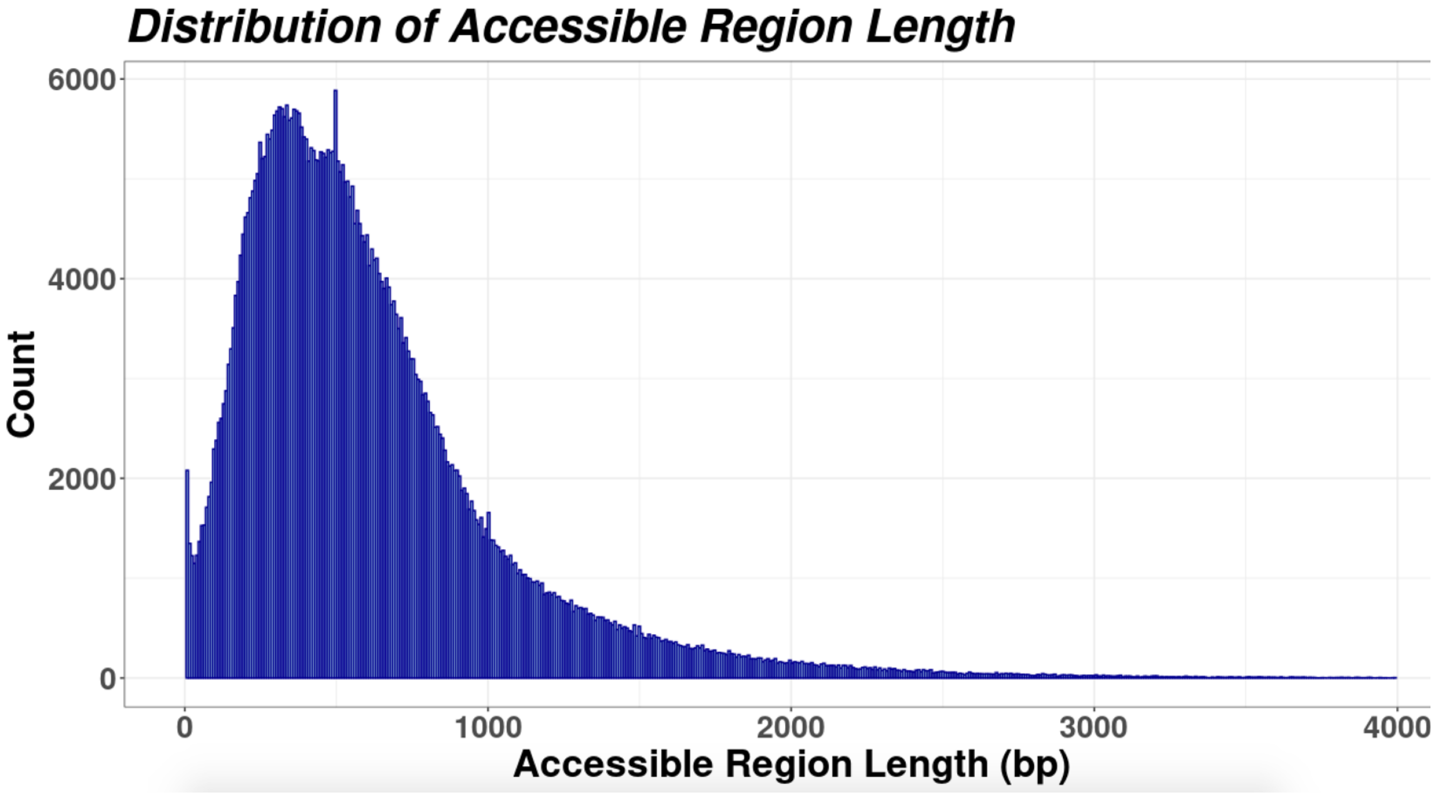

Fig S3: Distribution of peaks, across 10,246 samples, called using call-merge strategy. Peaks were individually called in each sample using macs2 callpeak with parameters "-f BED -g hs --nomodel --shift -100 --extsize 200 --max-gap 100 -q 0.05". Peaks were retained if found in >50 samples, and peaks were merged if within 100bp of another peak.

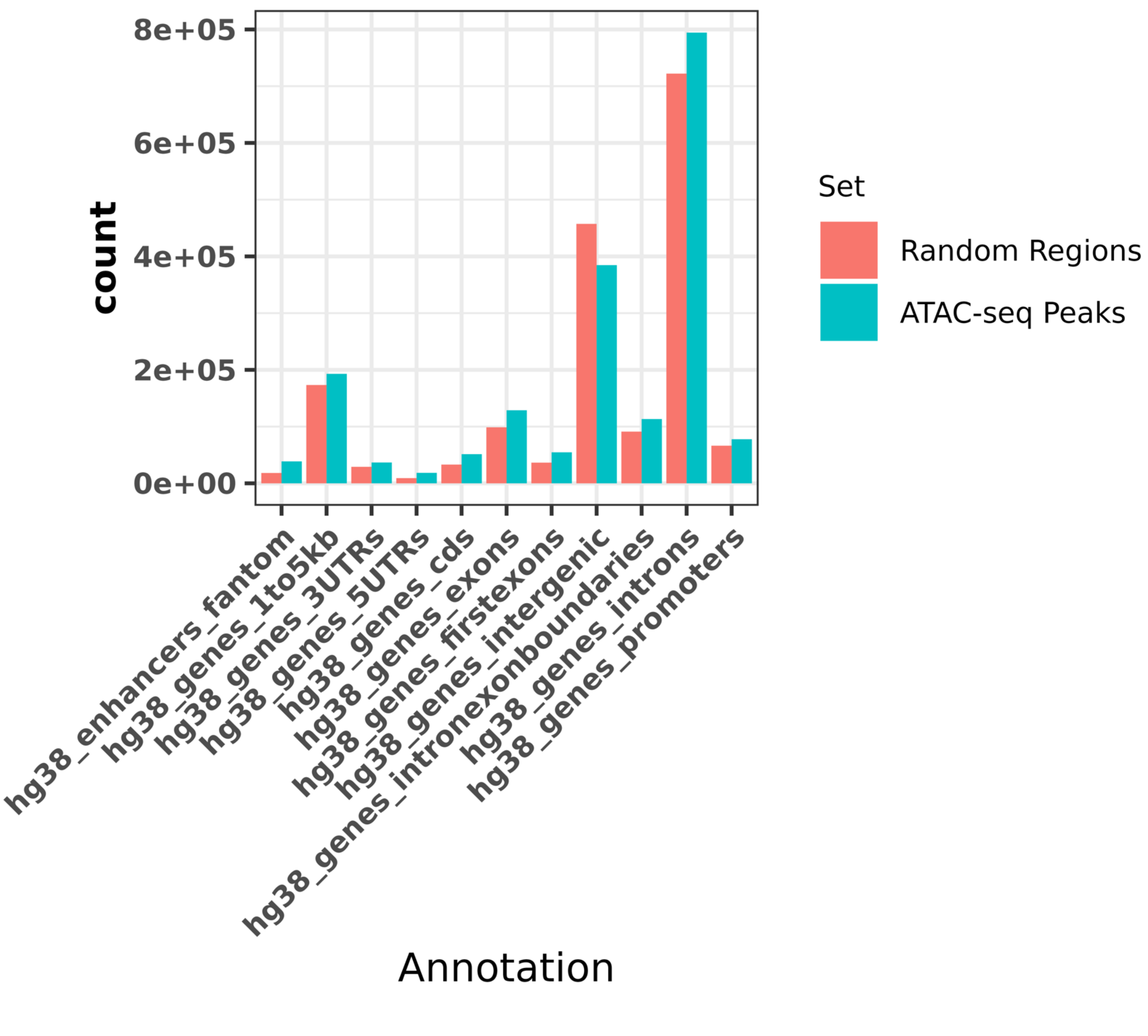

Fig S4: Enrichment of all called peaks, compared to matched random control regions, in various genomic annotation categories.

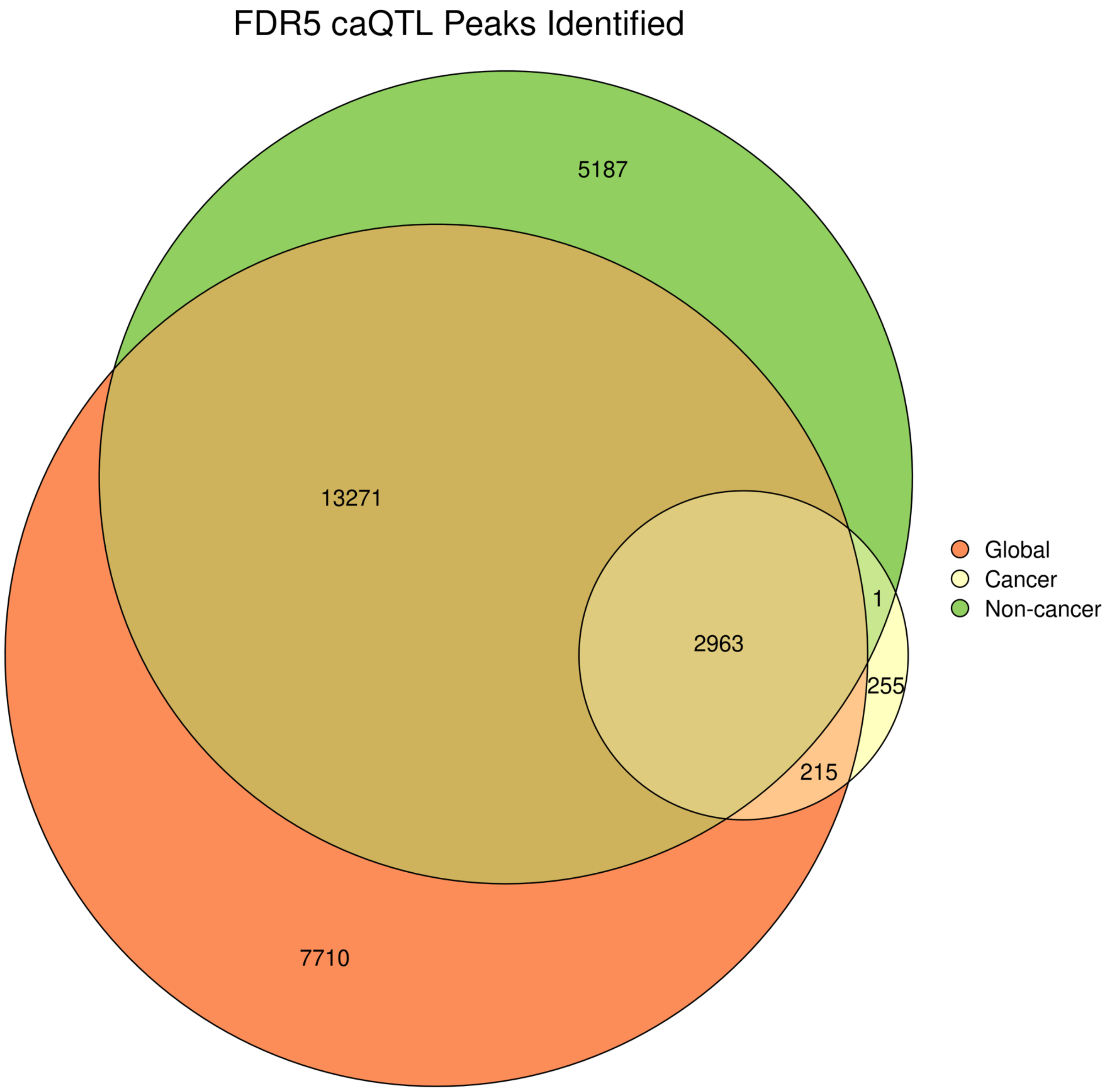

Fig S5: caQTLs were mapped separately in samples annotated as cancer-derived versus non-cancer. We then compared the results to the global analysis.

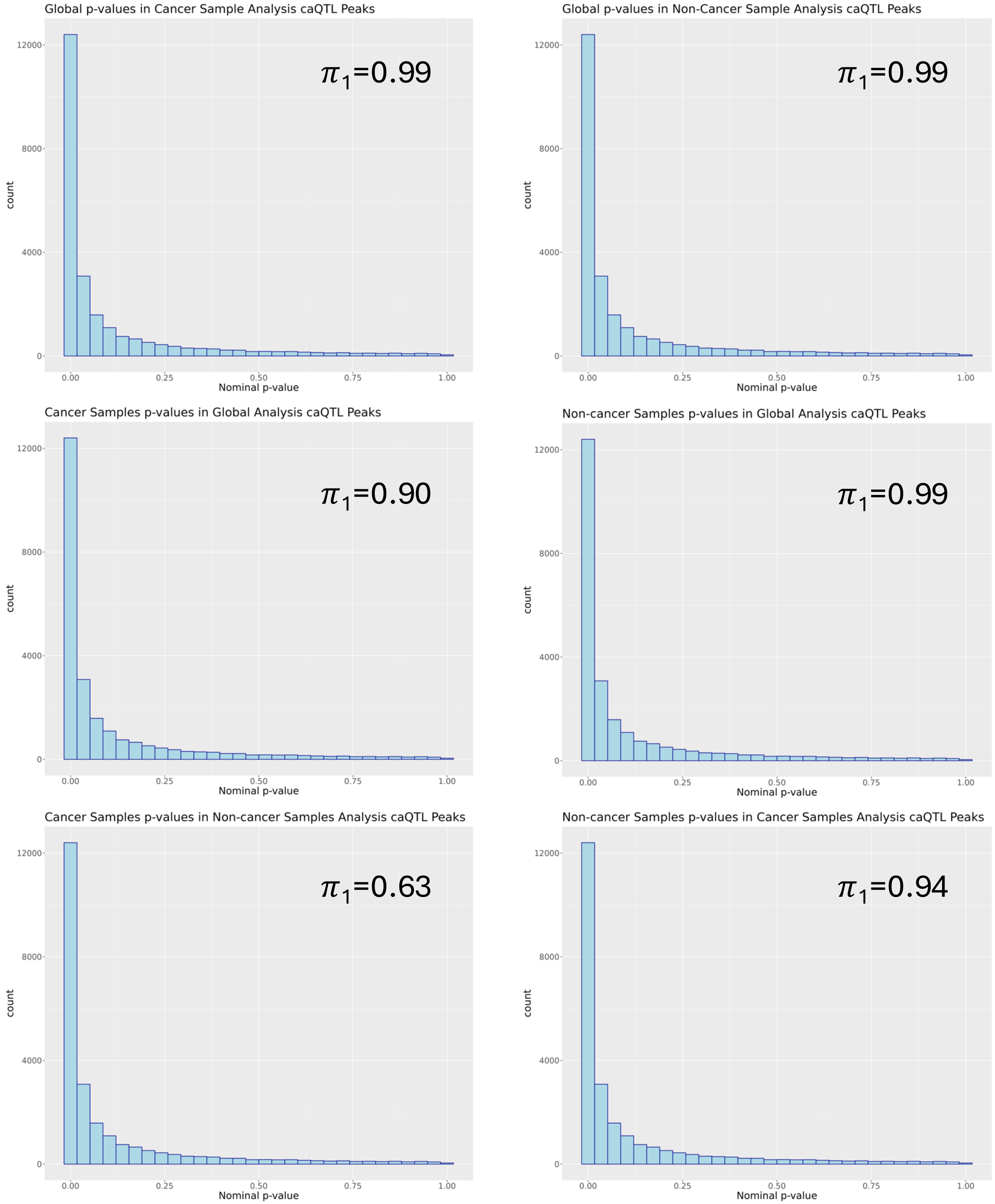

Fig S6: π_1 replication values and nominal p-values plotted for comparisons between global, cancer, and non-cancer sample analyses.

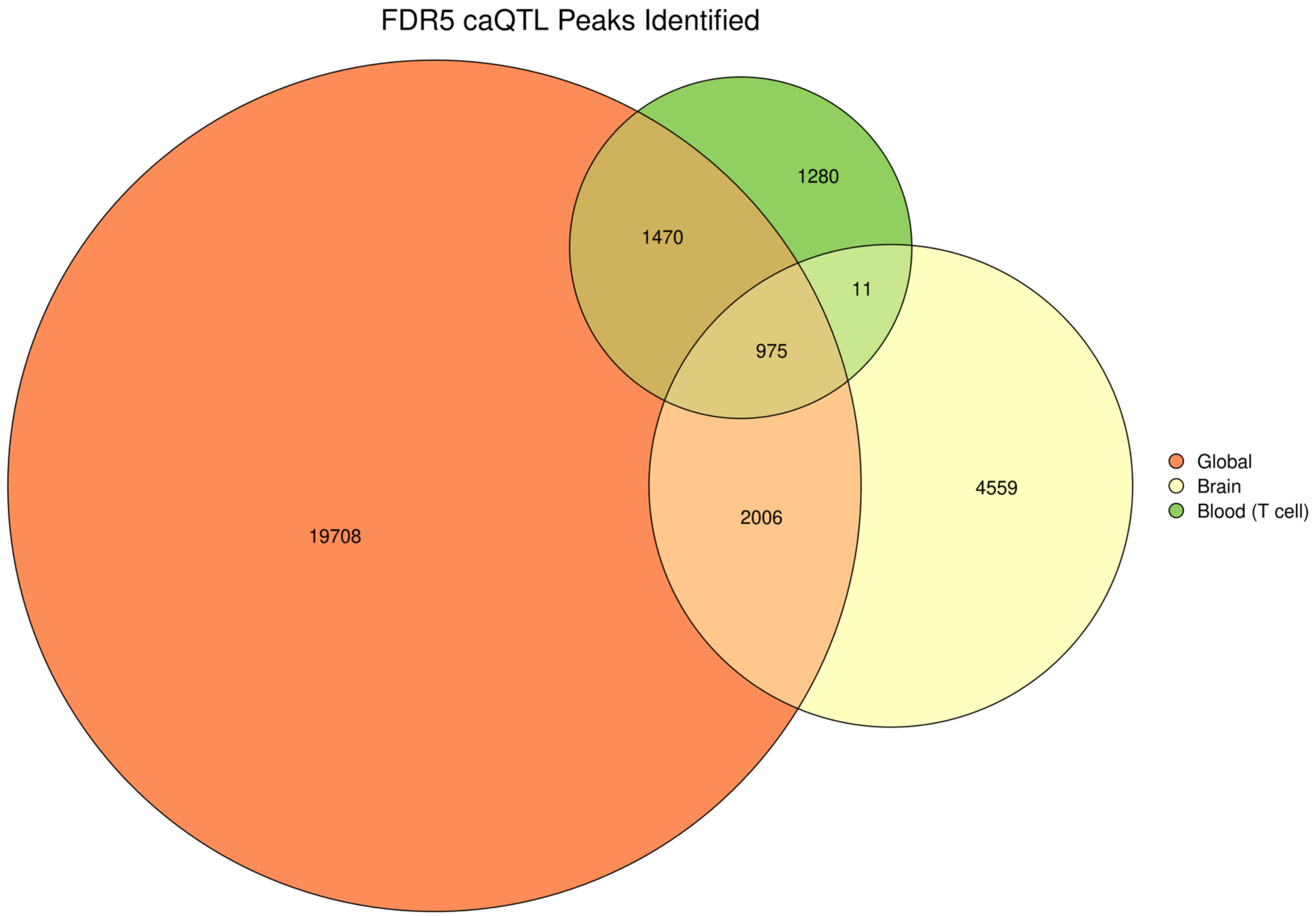

Fig S7: caQTLs were mapped separately in samples annotated as blood (T cell)-derived and brain-derived. We then compared the results to the global analysis.

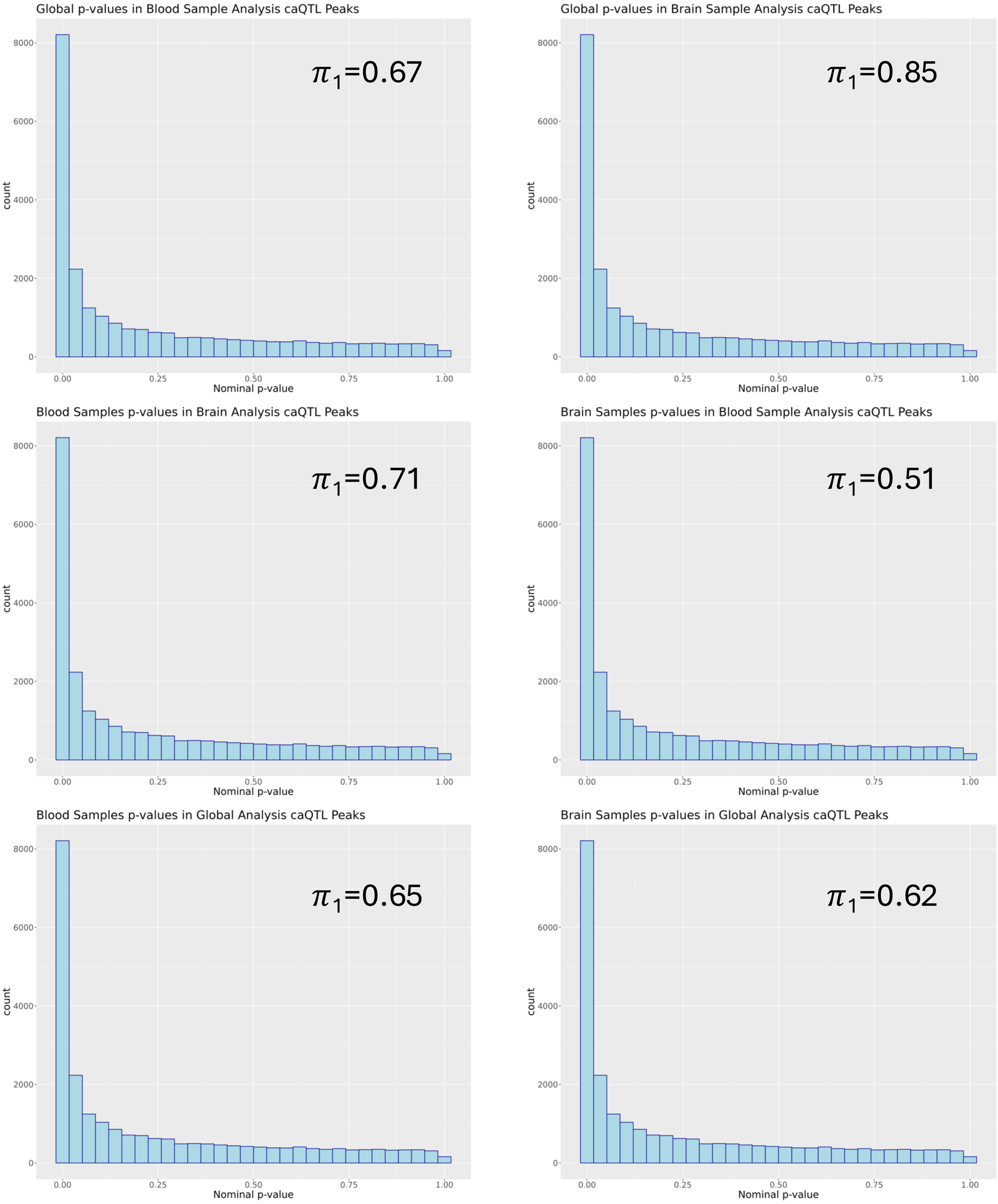

Fig S8: π_1 replication values and nominal p-values plotted for comparisons between global, brain, and blood (T cell) sample analyses.

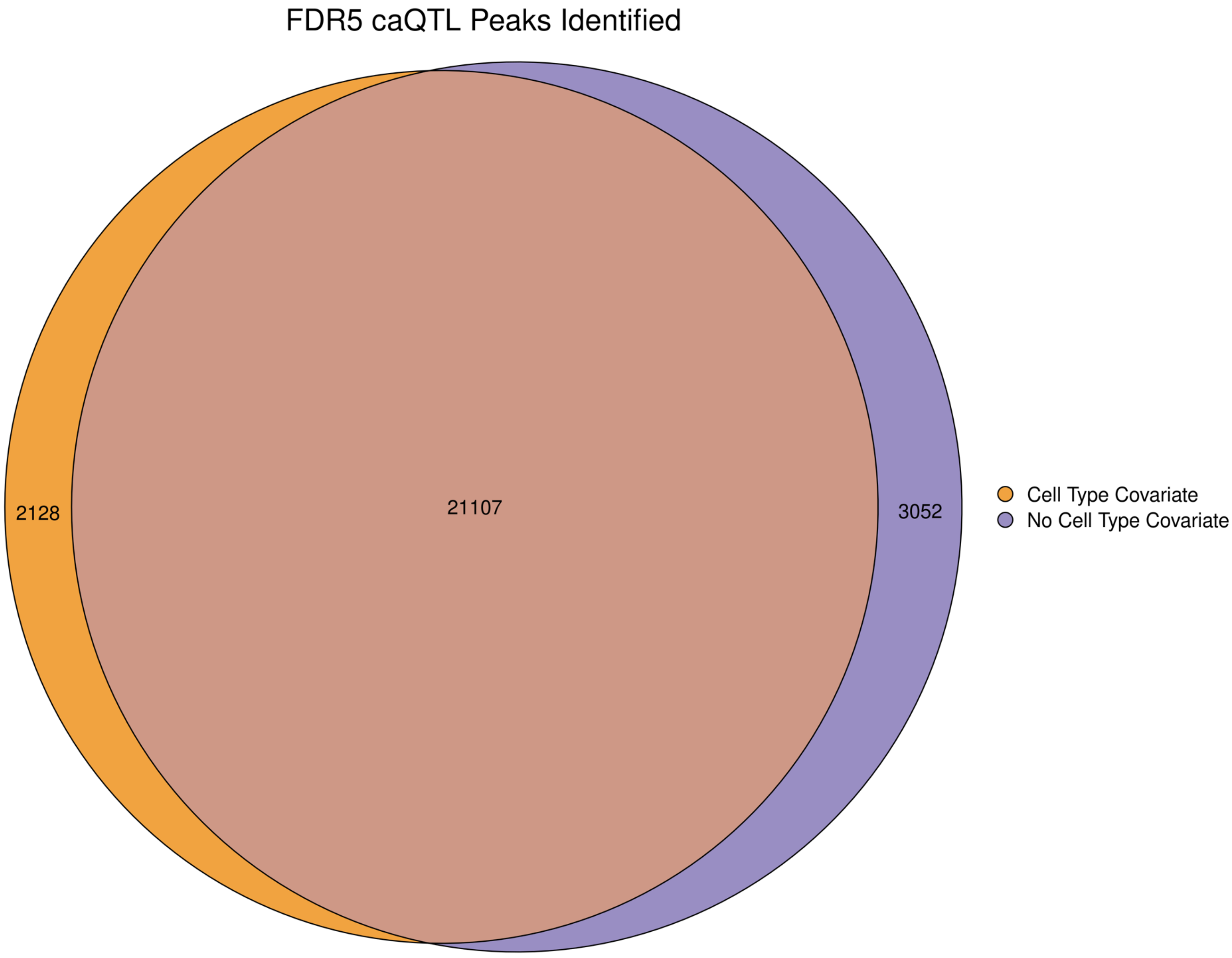

Fig S9: caQTLs were mapped including a covariate of annotated cell/tissue type and compared to the global analysis that did not include cell type as a covariate (π_1 replication = 0.99).

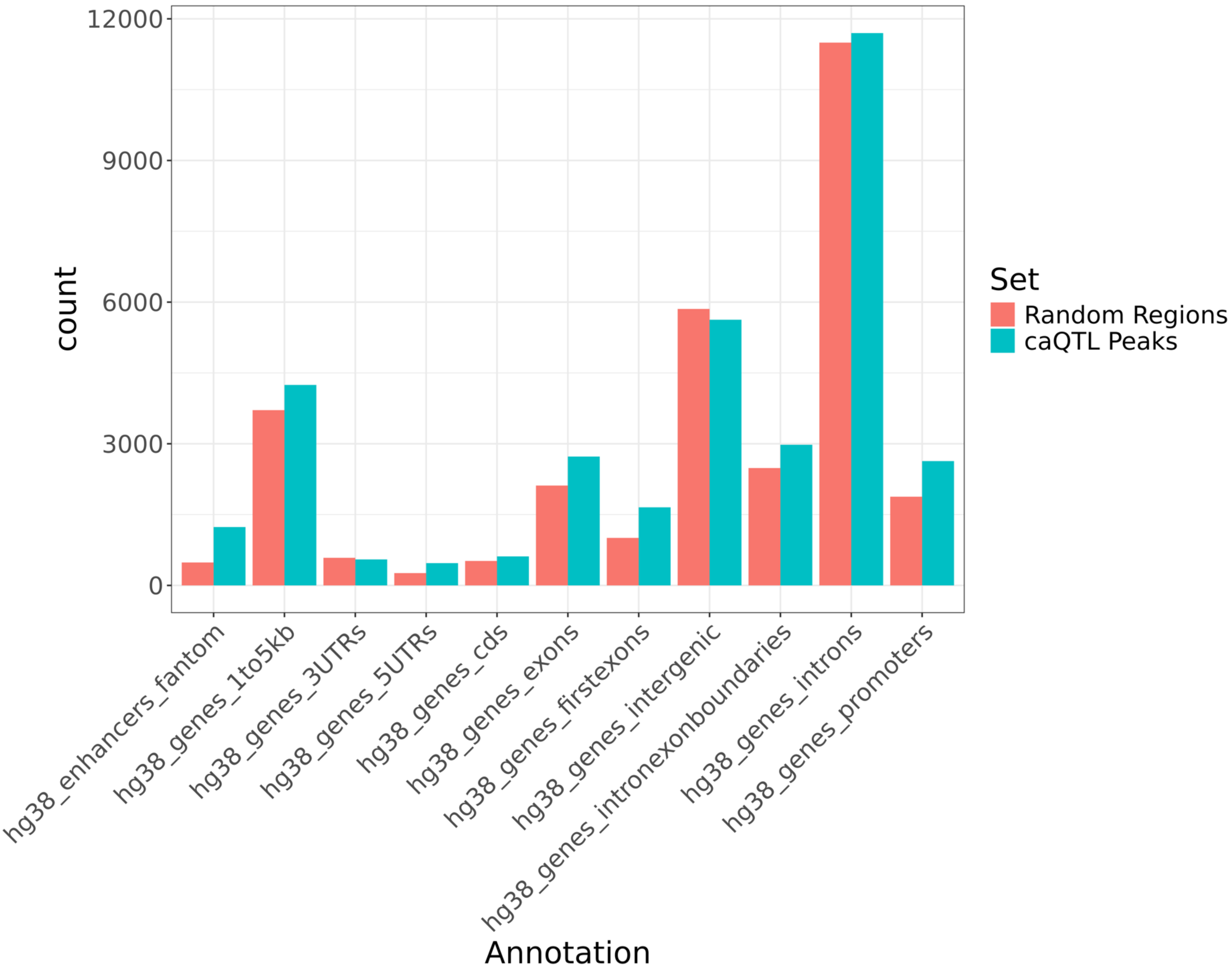

Fig S10: Enrichment of caQTL peaks, compared to matched random control regions, in various genomic annotation categories.

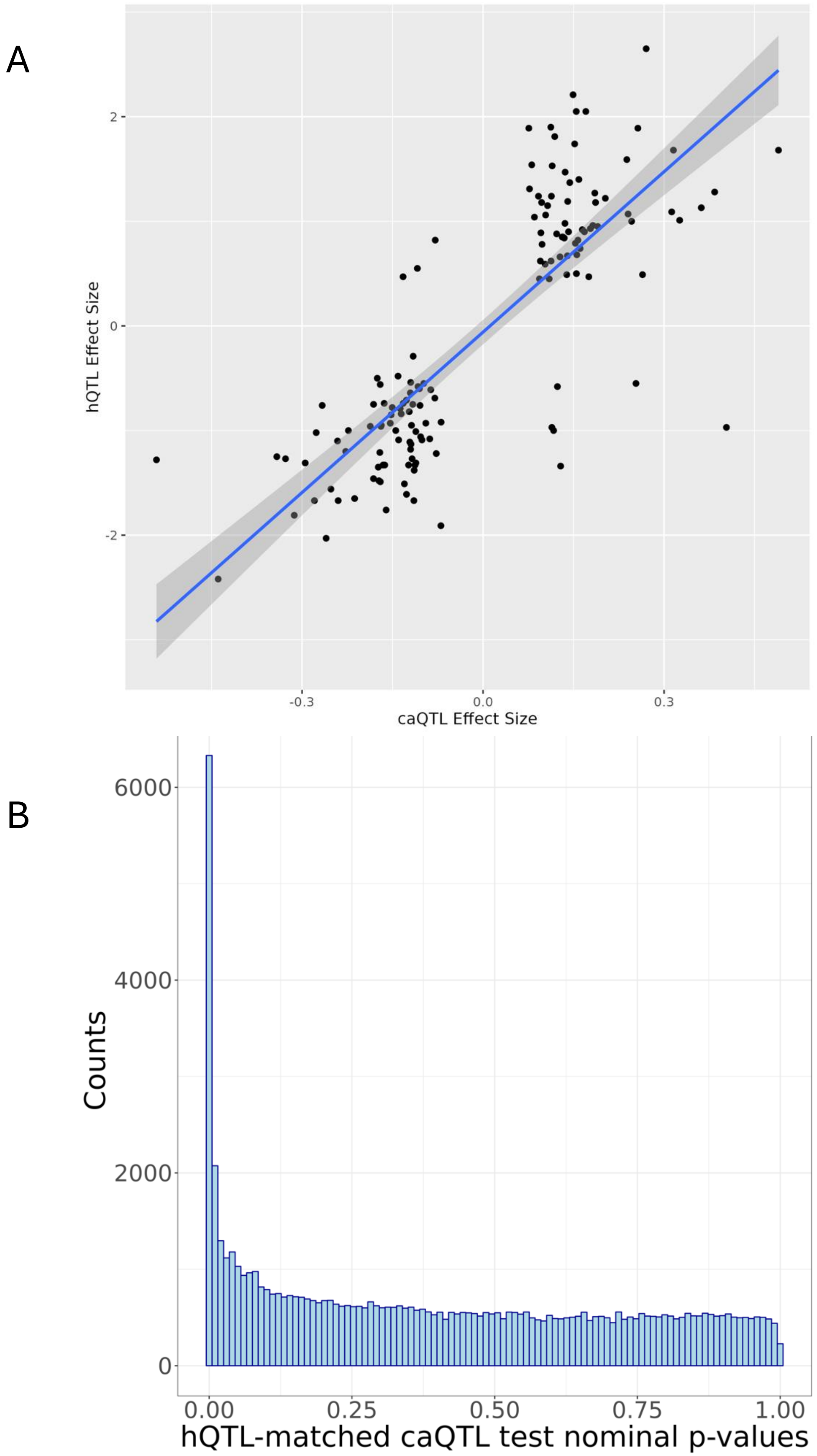

Fig S11: Histone QTLs (hQTLs) were downloaded from an external LCL hQTL dataset. 137 overlaps of 6,261 lead hQTLs were found with lead caQTLs compared to a median of 23 across 100 sets of background variants matched to lead caqtls on allele frequency and gene TSS distance. A) Directionality of matched lead caQTLs/hQTLs was consistent. B) 5300/6261 lead hQTL variants were tested in our global caQTL study. caQTL nominal p-values for hQTL variants were enriched for low p-values, suggesting enrichment of causal variant sharing across datasets.

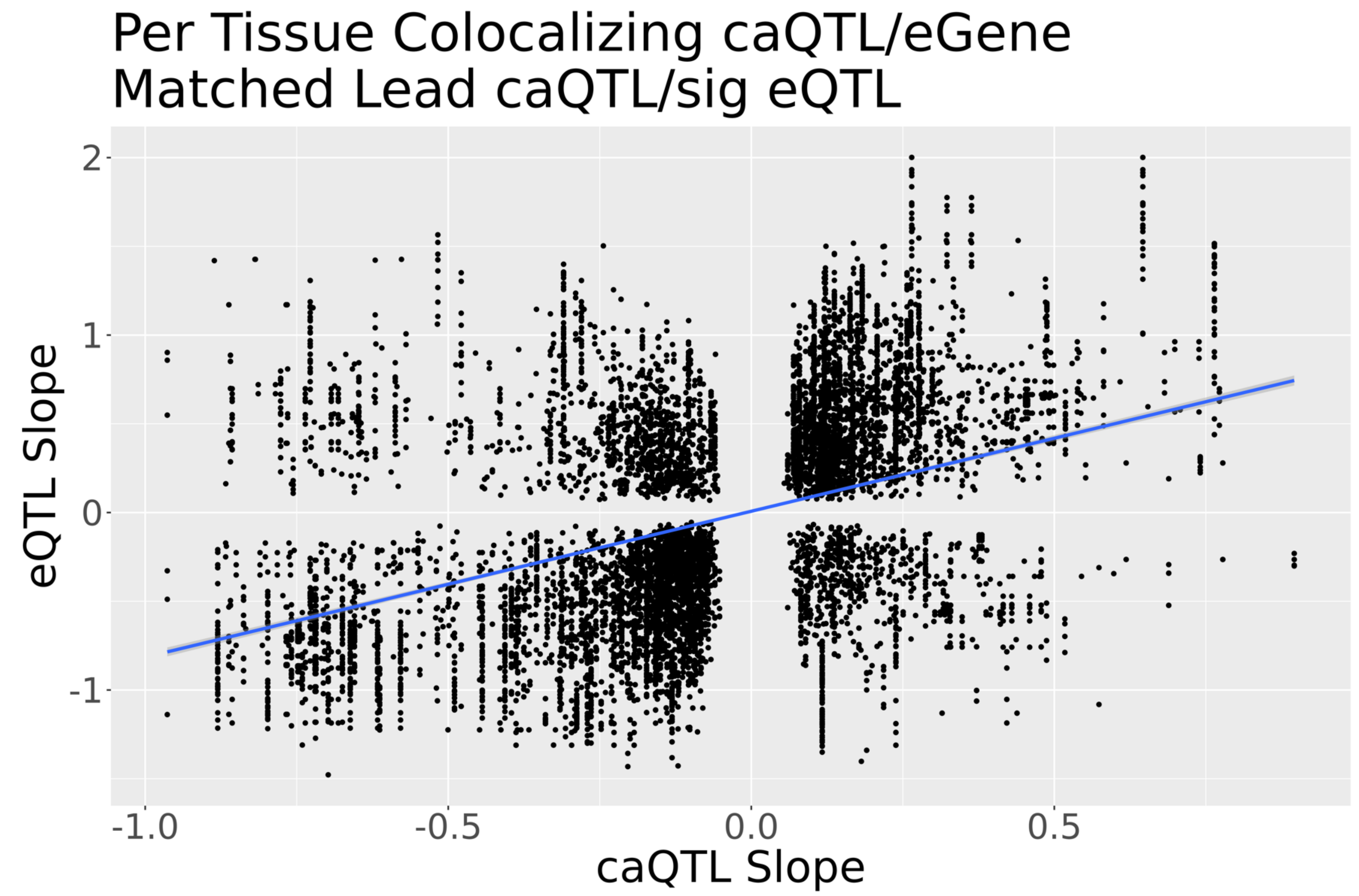

Fig S12: Across all tissues, lead caQTL variants that were also identified as significant lead eQTLs were retained from caQTL/eQTL colocalizations. We then plotted the slopes of the eQTL and caQTL for these variants to highlight the directionality of these variants.

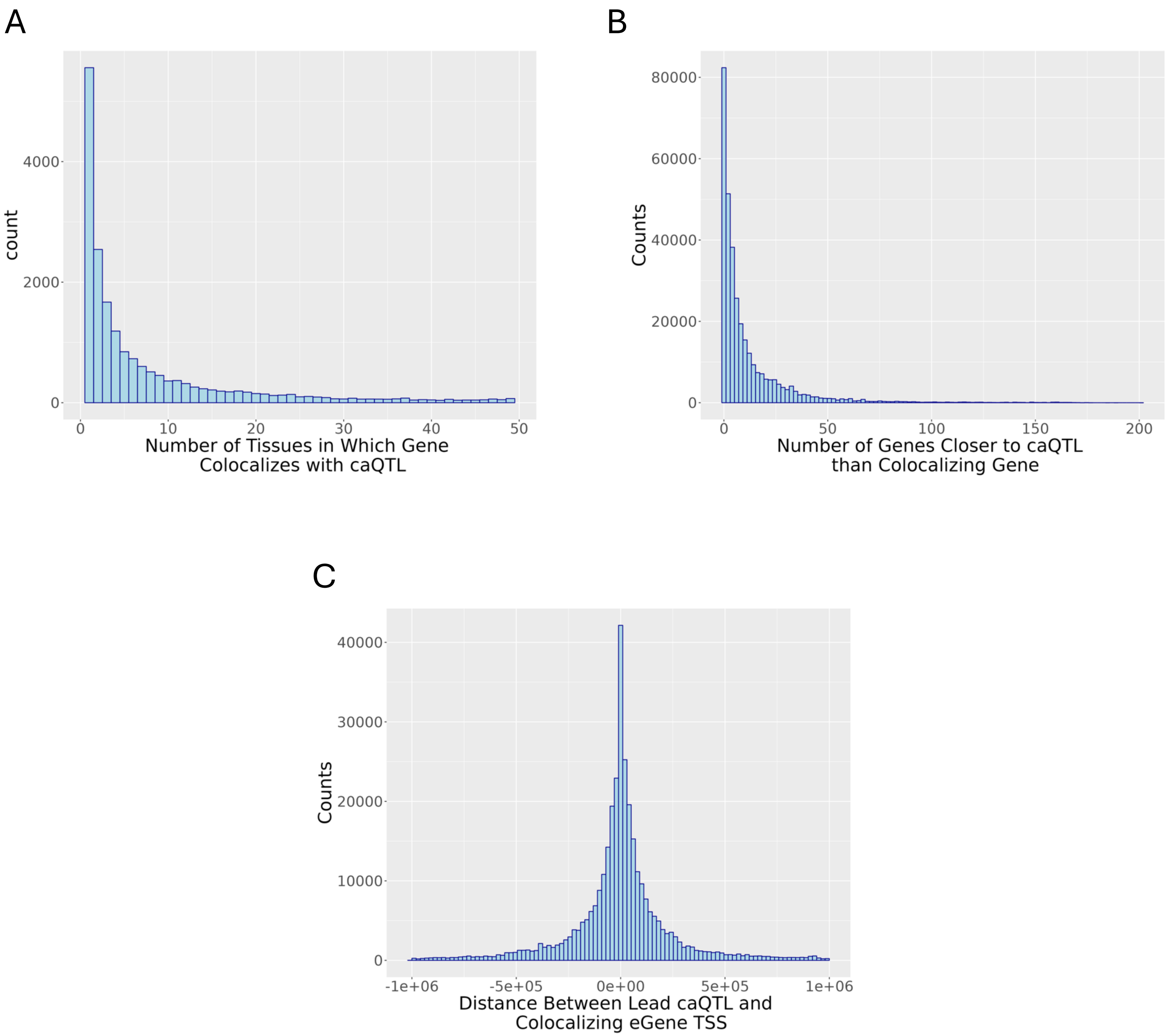

Fig S13: A. Number of GTEx tissues in which gene colocalized with caQTL. B. Number of gene TSSs closer to lead caQTL than gene caQTL colocalizes with. C. Distance between the lead caQTL position and the TSS of the colocalizing gene.

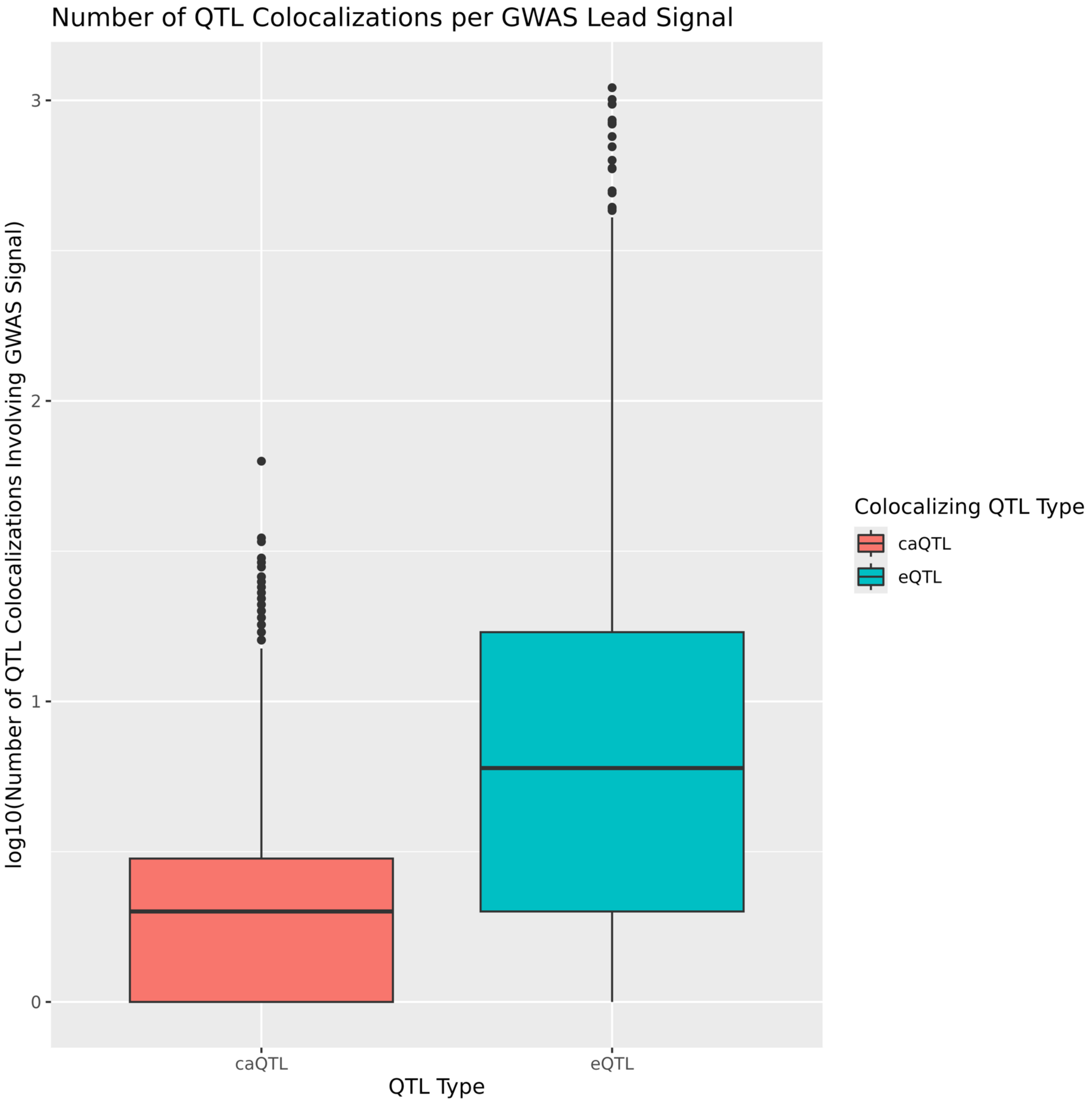

Fig S14: Colocalizations were performed between caQTL/GWAS and eQTL/GWAS and number of filtered colocalizations for each GWAS signal plotted.

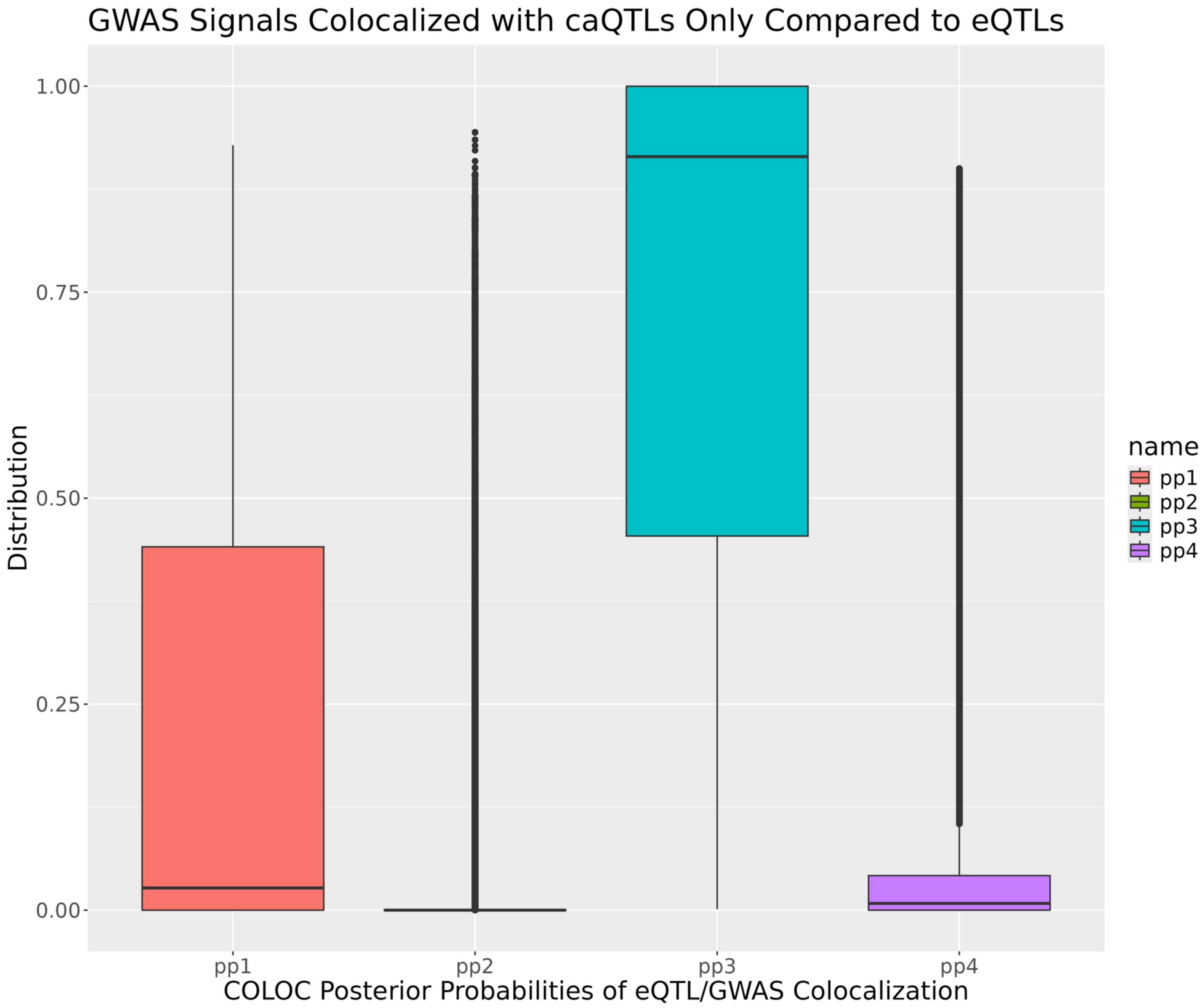

Fig S15: We identified GWAS signals that colocalized only with caQTLs and assessed the colocalization posterior probabilities for eQTLs/GWAS signals at these loci.

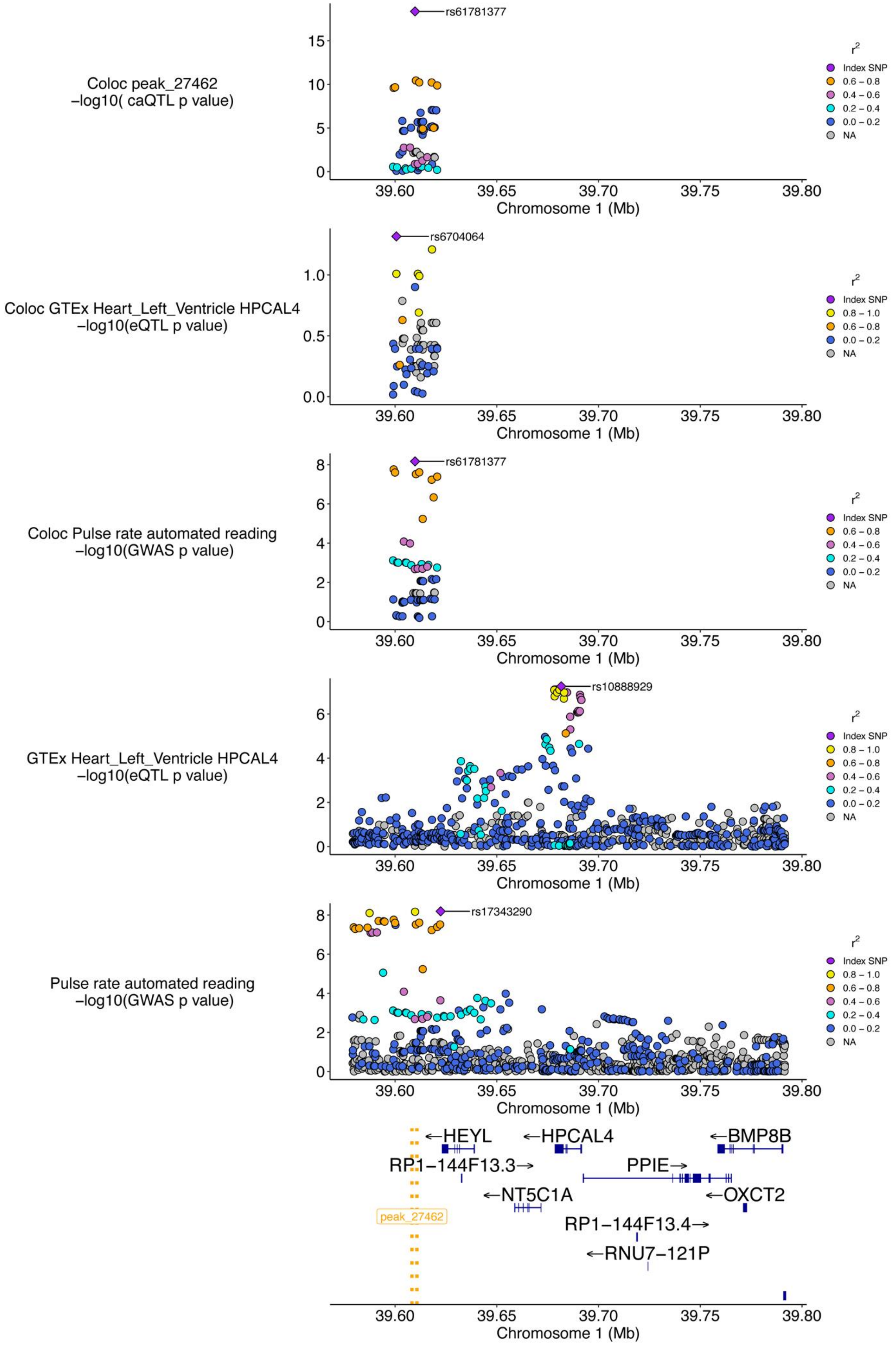

Fig S16: Shown is an example locus where a GWAS signal for pulse rate colocalized with a caQTL but not an eQTL. The results of the eQTL/GWAS colocalization suggested independent causal variants for the GWAS/eQTL (high COLOC PP3), which is evident in the stacked figure.

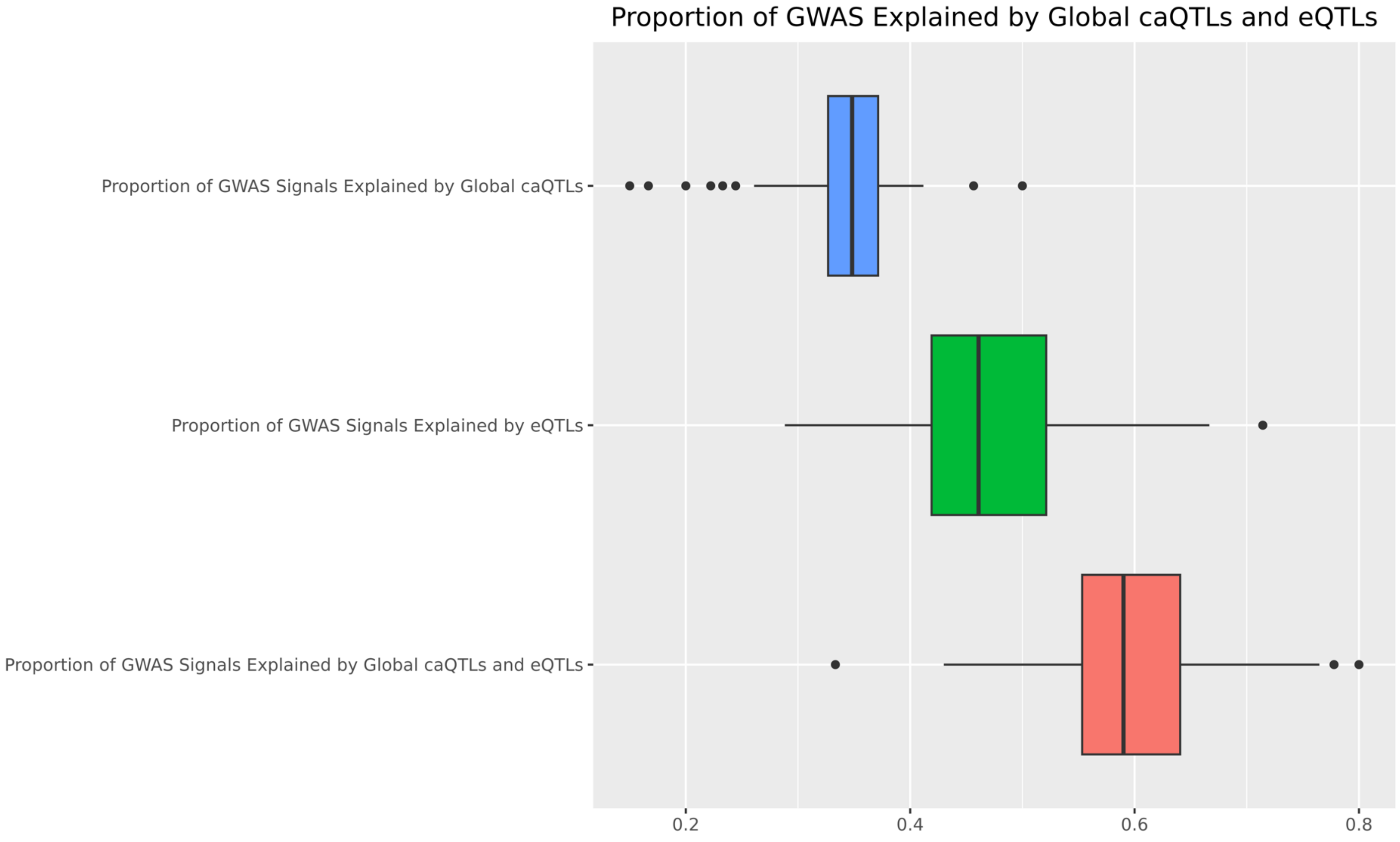

Fig S17: Colocalizations were performed between caQTL/GWAS and eQTL/GWAS. Each GWAS signal was checked to see if it colocalized exclusively with caQTLs, eQTLs, or colocalized with both. Proportion of tested GWAS signals that colocalized in each category are plotted.

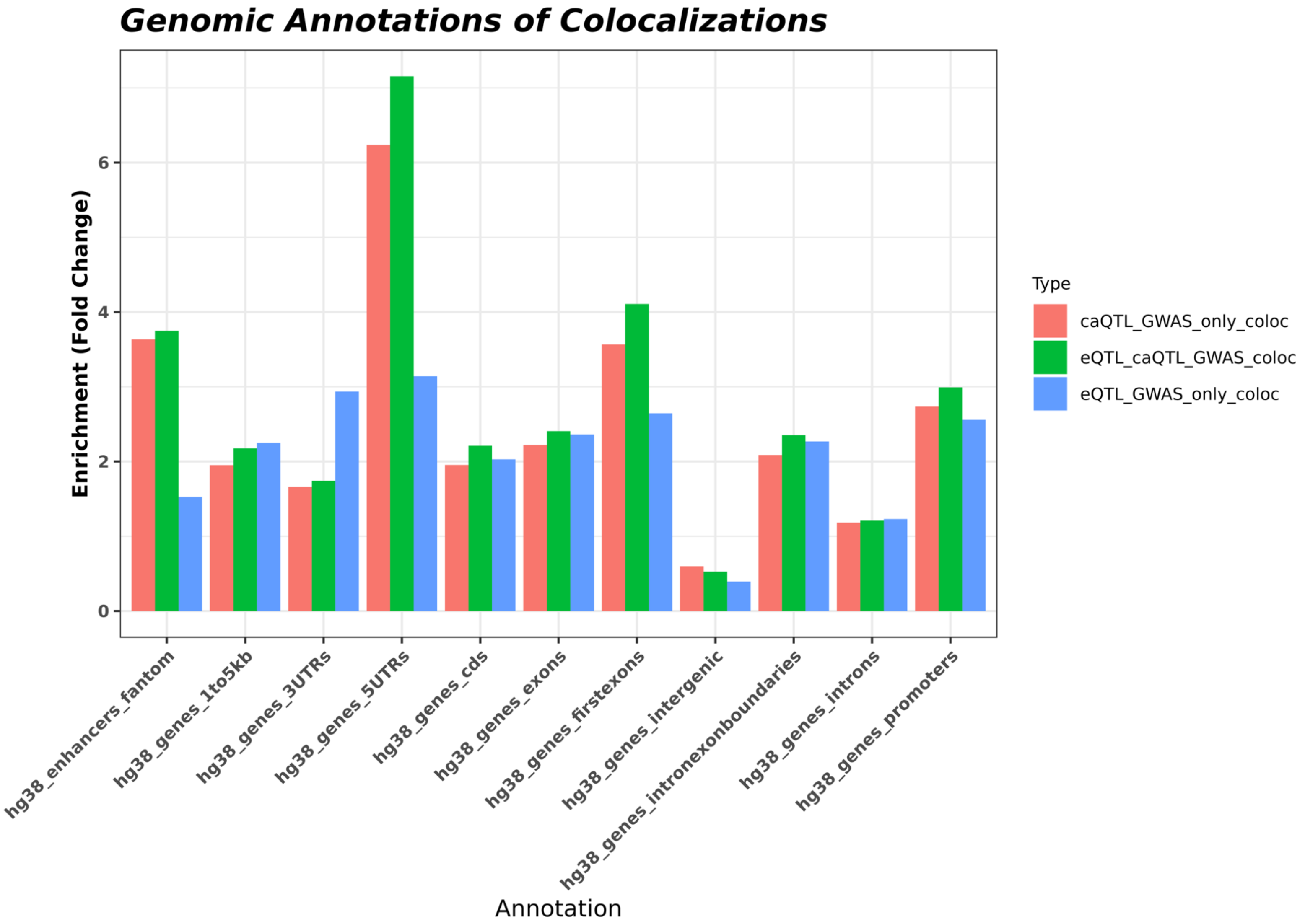

Fig S18: Enrichment of all colocalization categories compared to matched random control regions, in various genomic annotation categories. Lead caQTL (+/- 250 bp) used for enrichments for colocalizations involving caQTLs and lead eQTL (+/- 250 bp) used for enrichments for colocalizations involving eQTLs.

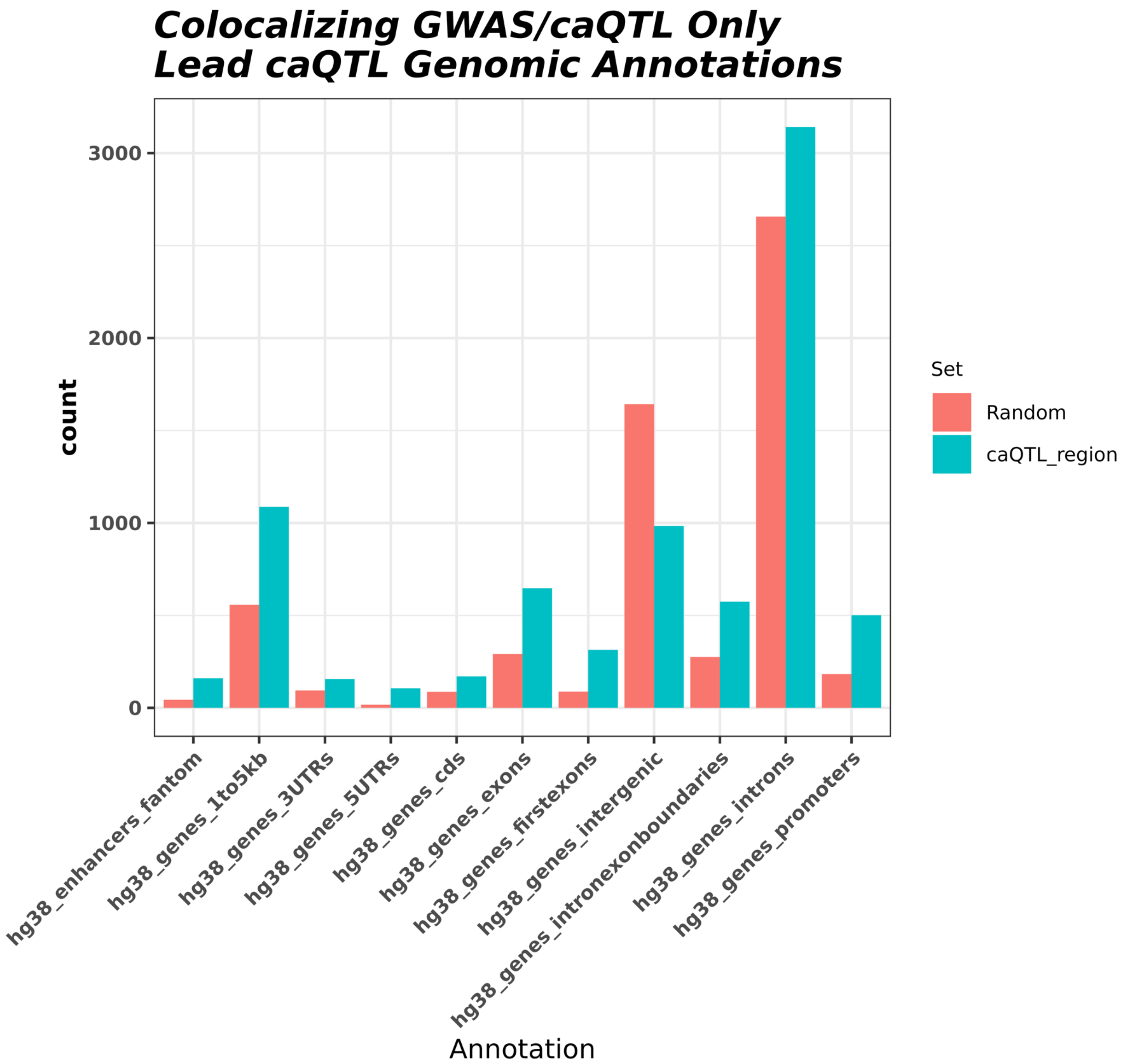

Fig S19: Enrichment of lead caQTL (+/- 250bp) from caQTL/GWAS colocalizations only, compared to matched random control regions, in various genomic annotation categories.

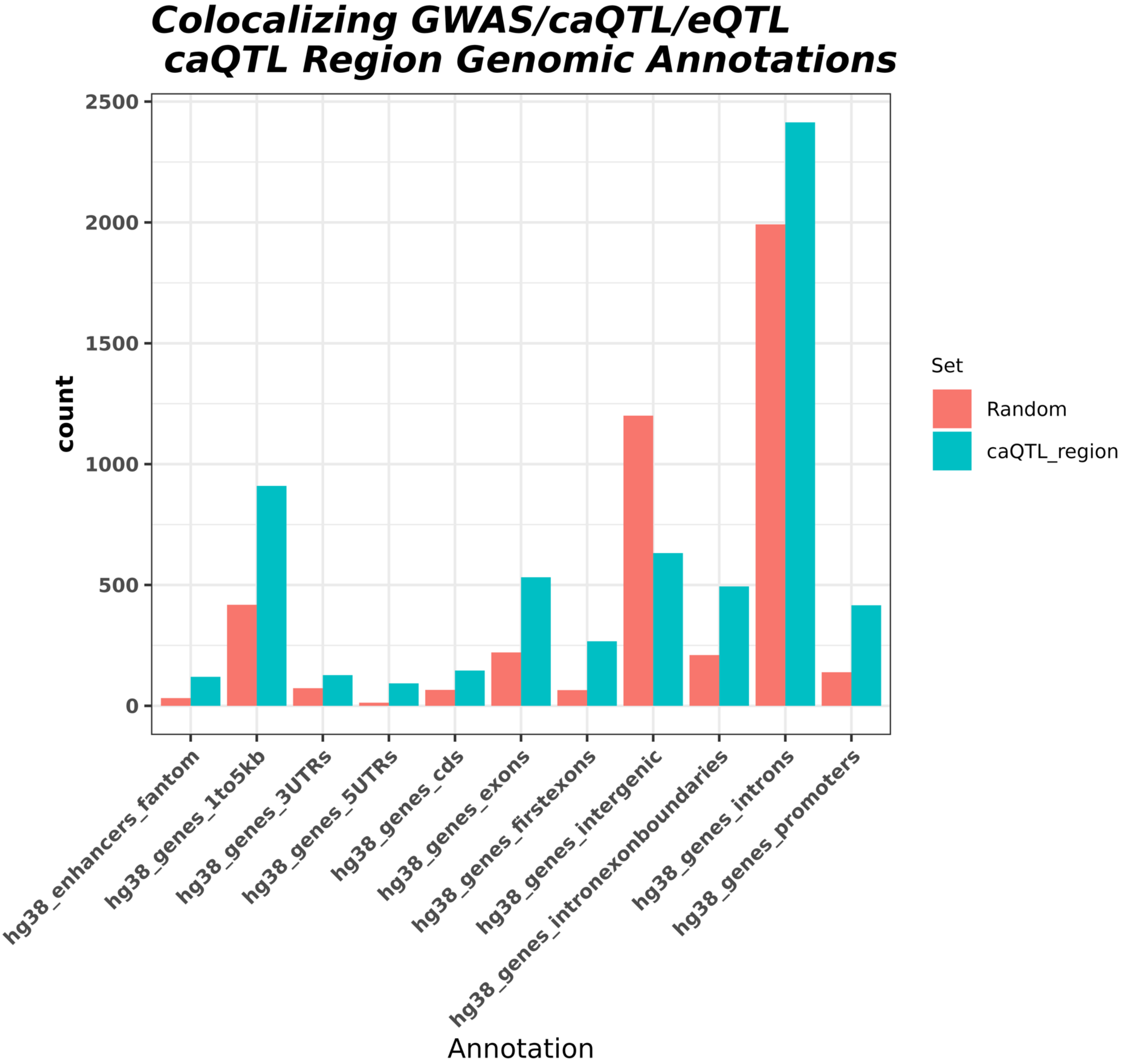

Fig S20: Enrichment of lead caQTL (+/- 250 bp) from caQTL/eQTL/GWAS colocalizations only, compared to matched random control regions, in various genomic annotation categories.

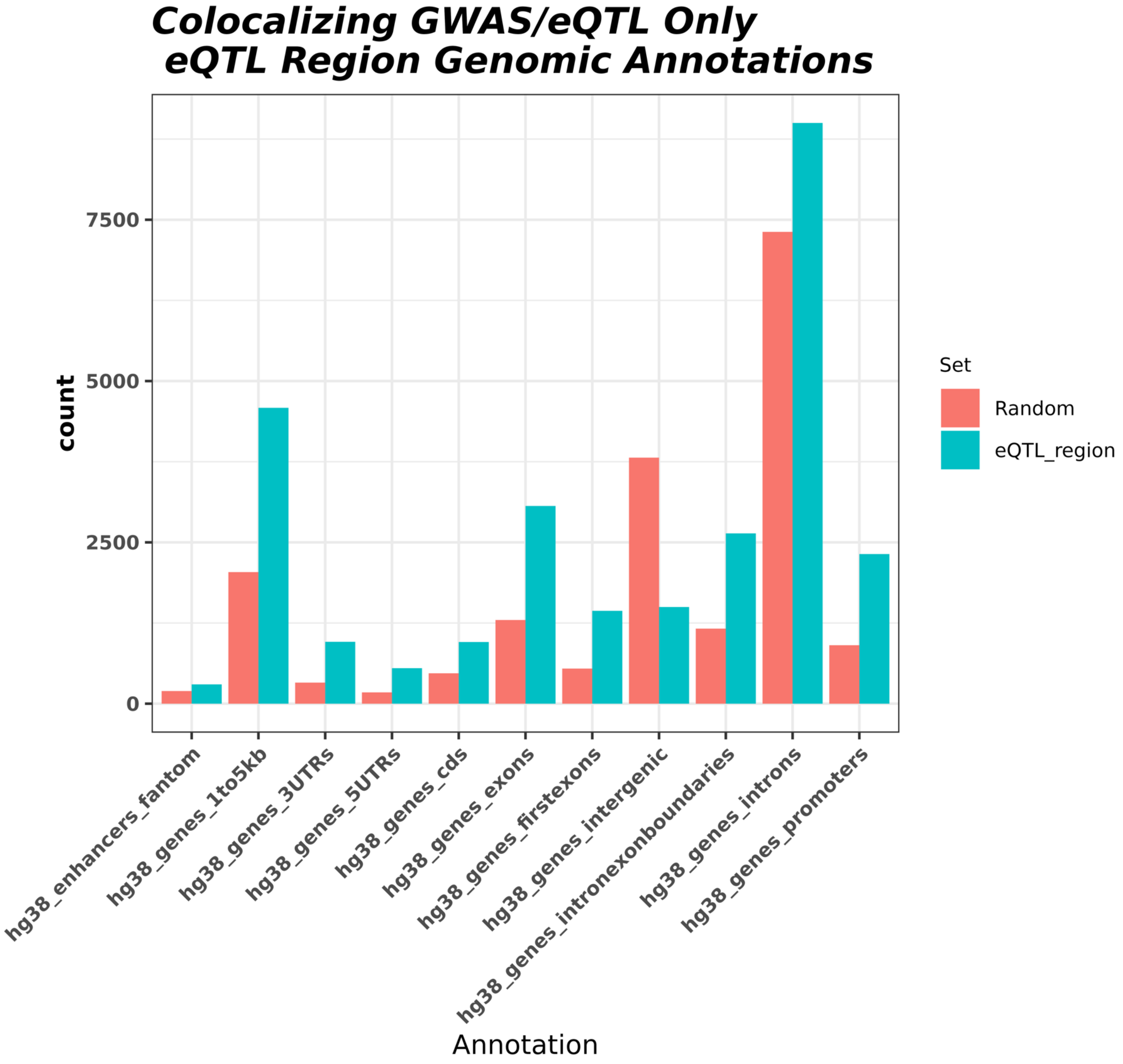

Fig S21: Enrichment of eQTL (lead variant +/- 250bp) from eQTL/GWAS colocalizations only, compared to matched random control regions, in various genomic annotation categories.

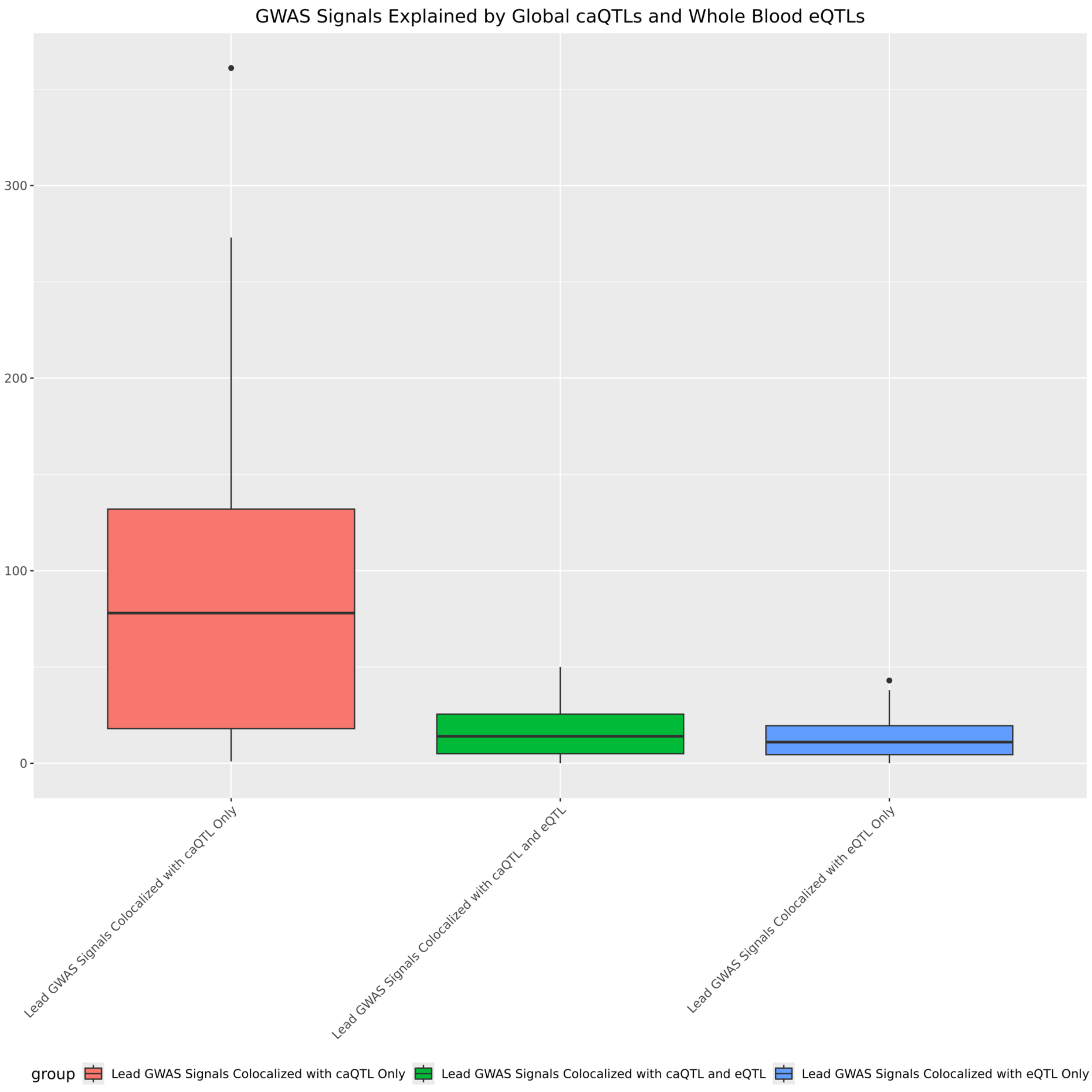

Fig S22: For each GWAS trait, independent lead GWAS variant signals were checked for colocalization with caQTL and eQTL signals in GTEx Whole Blood only. Plotted is the number of unique lead GWAS variants per colocalization group, as multiple caQTL peaks, eGenes, etc. can colocalize with the same lead GWAS signal.

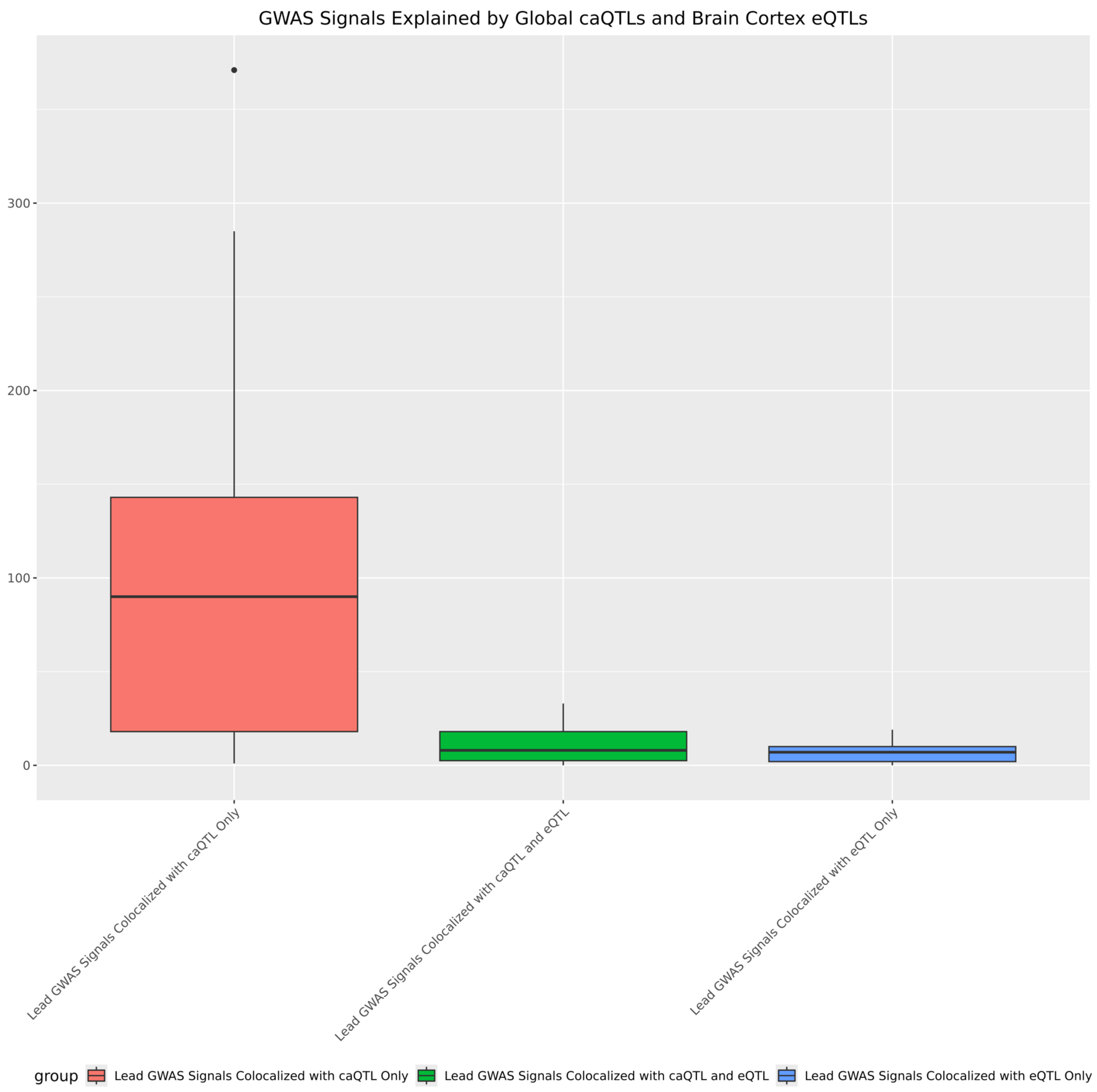

Fig S23: For each GWAS trait, independent lead GWAS variant signals were checked for colocalization with caQTL and eQTL signals in GTEx Brain Cortex only. Plotted is the number of unique lead GWAS variants per colocalization group, as multiple caQTL peaks, eGenes, etc. can colocalize with the same lead GWAS signal.

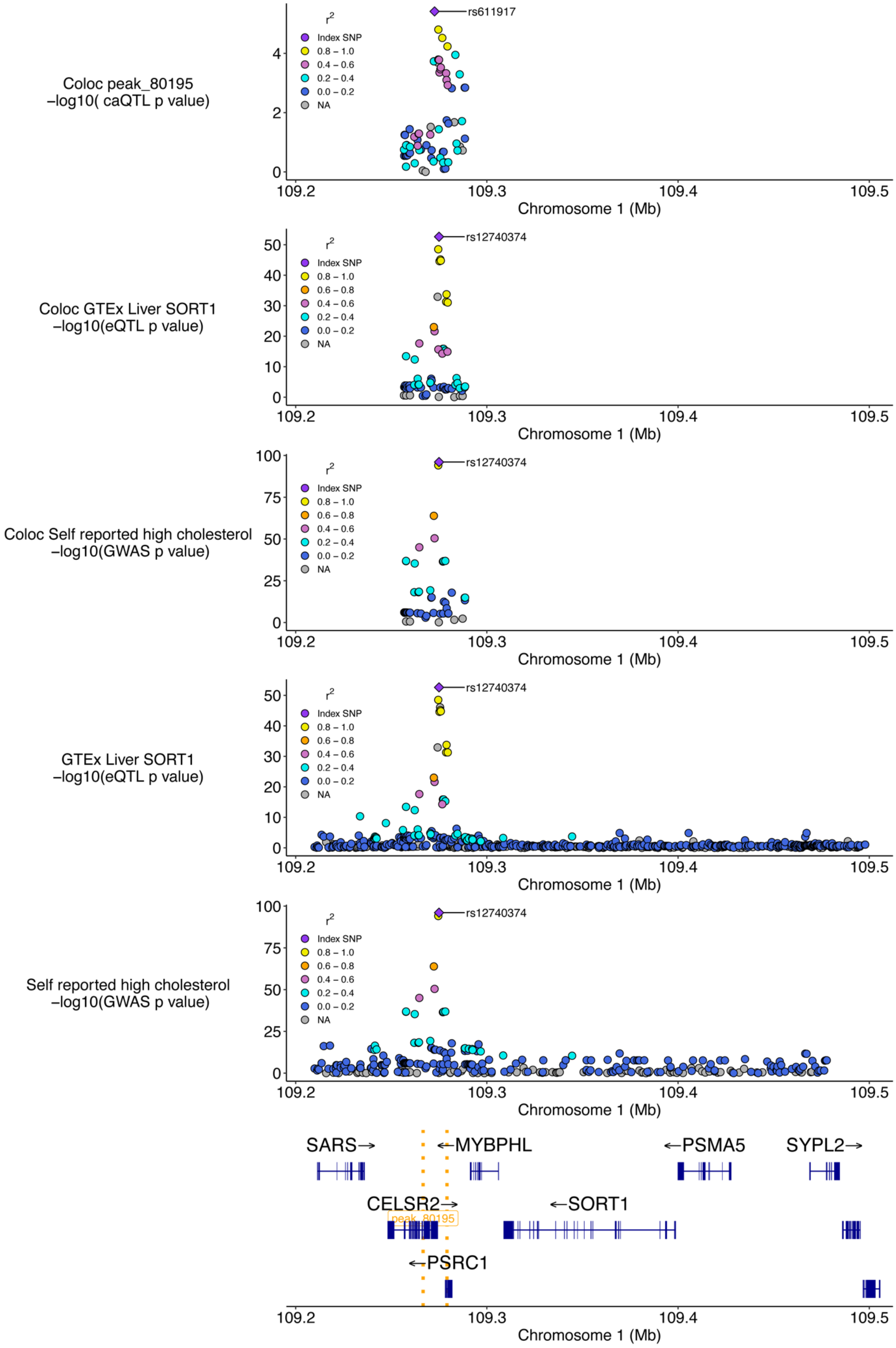

Fig S24: caQTL peak at well-studied LDL GWAS locus colocalizes with *SORT1* in liver and GWAS signal for high cholesterol. The top three plots are the colocalization windows (10kb + caQTL peak) for the caQTL, eQTL, and GWAS, respectively. The following two plots are showing a larger window to illustrate the eQTL and GWAS signals, respectively, at this locus at a different scale. The bottom gene track highlights the position of genes at this locus, as well as the location of the caQTL peak (gold dotted lines).

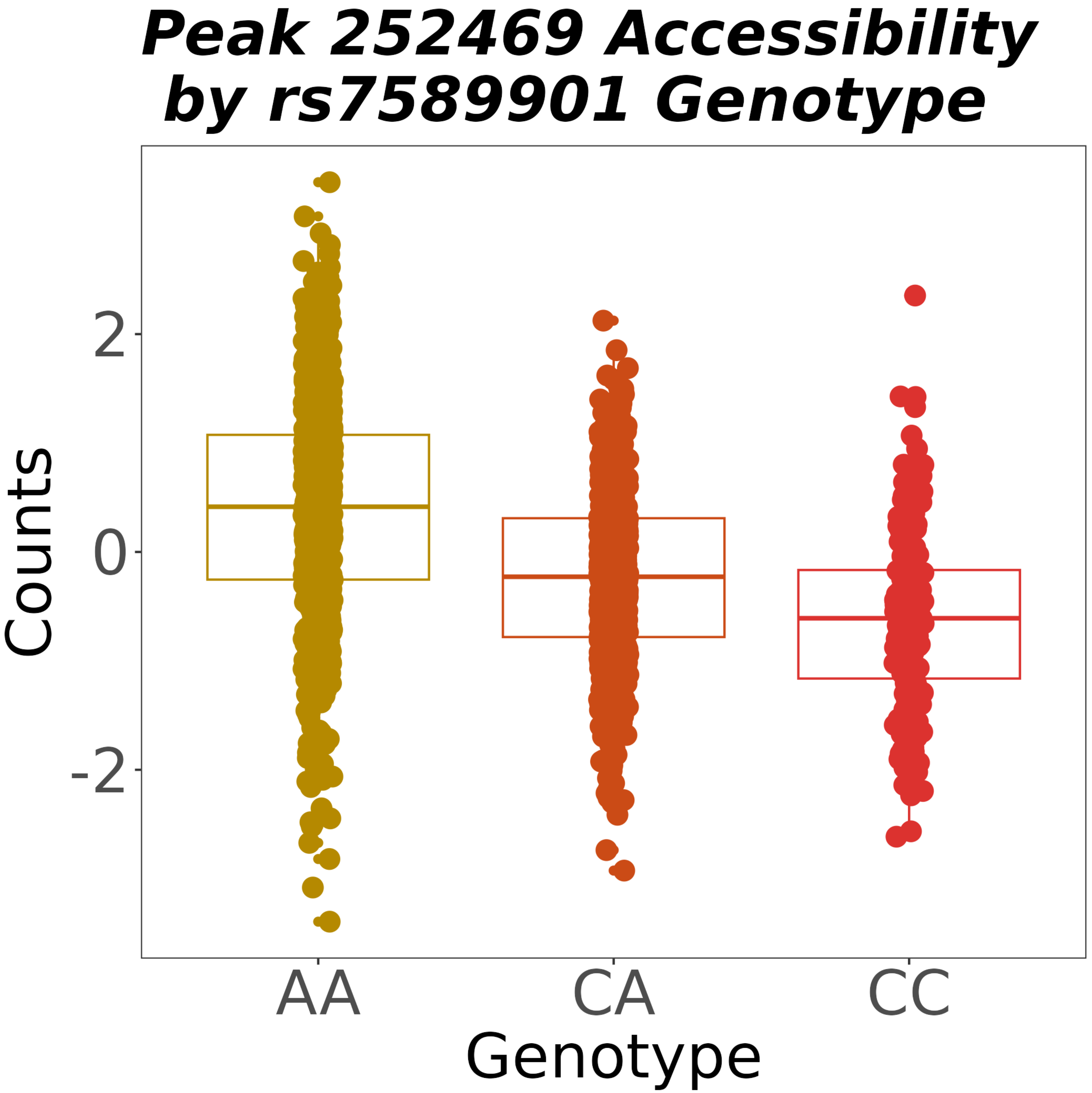

Fig S25: Sample normalized read counts, grouped by genotype, at peak 252469, which colocalized with *PAX8* eQTL in Whole Blood and Urea serum levels. AA individuals have increased chromatin accessibility compared to CA and CC individuals.

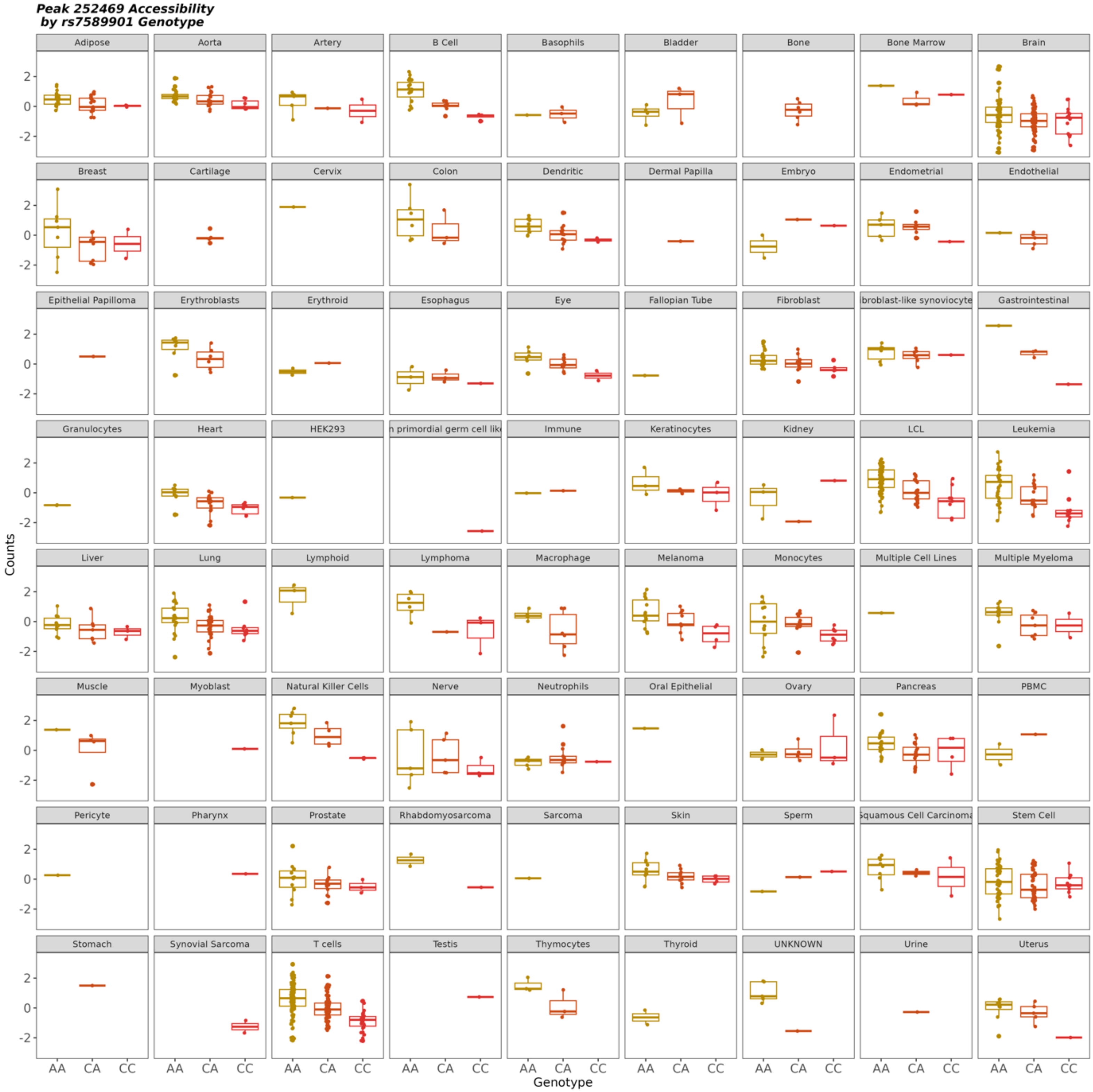

Fig S26: Sample normalized read counts, grouped by genotype, at peak 252469, which colocalized with *PAX8* eQTL in Whole Blood and Urea serum levels. Shown is genotype-accessibility relationship across all assigned groups of samples.

3 models available for ZNF135 at chr2:113235767A>C.1

Database	Model ID	WT score	MT score	WT start	WT end	MT start	MT end	WT strand	MT strand	Prediction	Score
jaspar2022	MA1587.1	0.8607	0.9376	-2	11	-2	11	plus	plus	gain	0.3169
jaspar2022DetailedTFFMs	TFFM0632.1	0.0309	0.8987	-2	11	-2	11	plus	plus	gain	0.9949
jaspar2022FirstOrderTFFMs	TFFM0632.1	0.4564	0.9944	-2	11	-2	11	plus	plus	gain	0.9983

(The combined score reflects only the TFFMs.) **Combined** gain 0.9966

Sequences

	-14	-13	-12	-11	-10	-9	-8	-7	-6	-5	-4	-3	-2	-1	0	+1	2	3	4	5	6	7	8	9	10	11	12	13	14	15	16
WT	G	G	G	G	A	G	A	G	A	G	A	A	G	C	T	A	G	A	C	C	T	C	C	C	T	G	C	T	G	C	G
MT	G	G	G	G	A	G	A	G	A	G	A	A	G	C	T	C	G	A	C	C	T	C	C	C	T	G	C	T	G	C	G

Known TFBSs at position

ENCODE: ATF7, BHLHE40, CBFB, CTCF, E4F1, ELF1, FOXK2, GMEB1, IKZF1, IKZF2, MAX, MGA, MNT, MTA3, MYC, NFIC, PML, RBFOX2, RELB, SIN3A, SMAD5, TAF1, YY1, ZFX

Ensembl: ATF7, E2F6, ELF1, ELF4, FOXK2, GMEB1, IKZF1, IKZF2, KLF5, MGA, MNT, MYC, NR2F1, POU2F2, RELB, SIN3A, SP1, TAF1, TBP, ZBTB7A

FANTOM5: -

Sequence logos

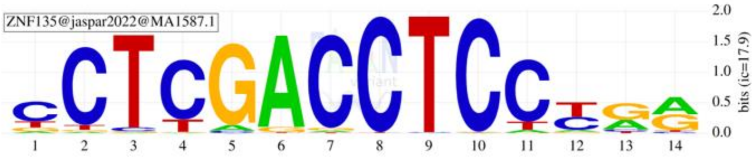

Motif display help

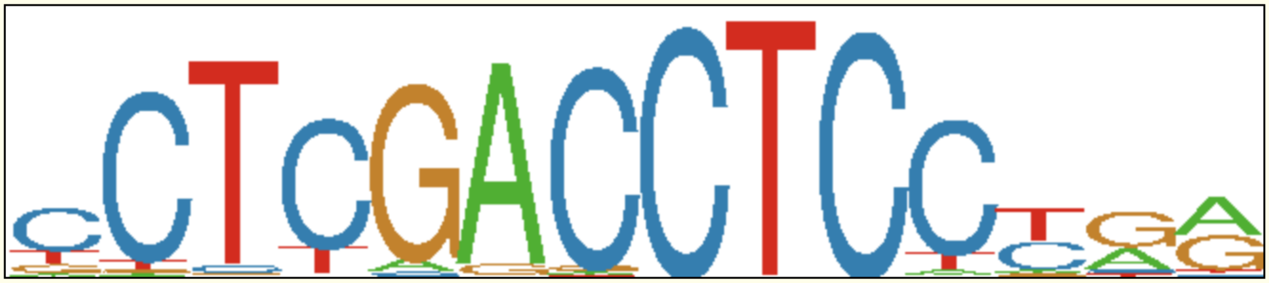

	G	C	T	A	G	A	C	C	T	C	C	C	T	G	this occurrence
	c	**C**	**T**	C	**G**	**A**	**C**	**C**	**T**	**C**	C	t	g	a	motif consensus
A	0.05	0.02	0.00	0.01	0.06	0.93	0.02	0.00	0.00	0.00	0.04	0.06	0.32	0.47	
C	0.63	0.91	0.04	0.81	0.03	0.00	0.95	0.99	0.01	0.99	0.84	0.42	0.07	0.04	
G	0.13	0.02	0.02	0.01	0.91	0.05	0.03	0.00	0.00	0.01	0.01	0.05	0.55	0.44	
T	0.19	0.05	0.94	0.17	0.00	0.01	0.01	0.01	0.99	0.00	0.11	0.47	0.06	0.05	

Fig S27: Lead caQTL variant at caQTL/eQTL/GWAS colocalizing locus is rs7589901. Motif analysis predicts that *ZNF135* will bind to motif overlapping rs7589901. The alternate 'C' allele at position 4 is predicted to increase binding affinity.

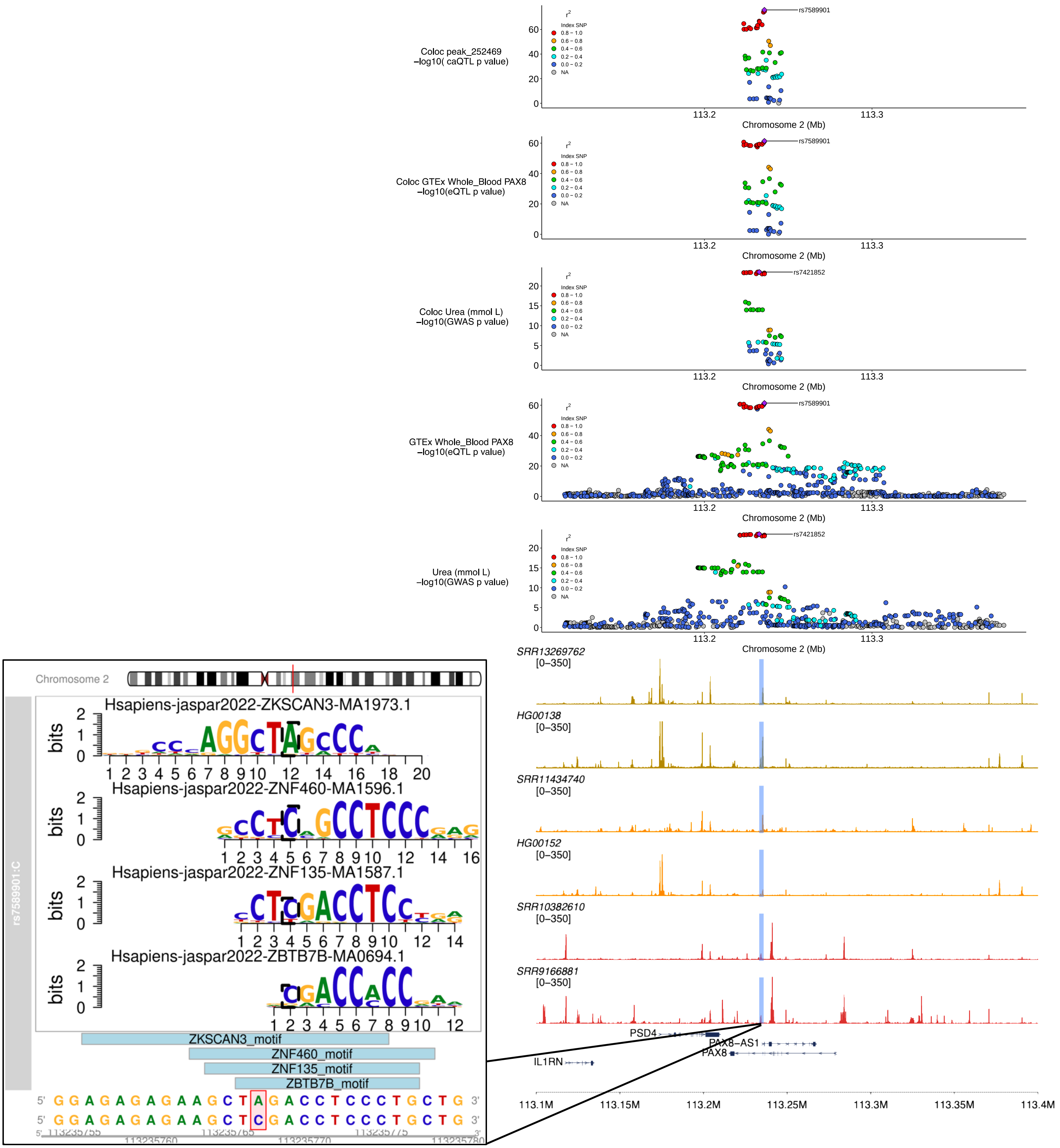

Fig S28: Locus where caQTL/eQTL/GWAS colocalization nominates *PAX8*, measured in whole blood, as the causal gene underlying blood urea levels. The lead caQTL variant is predicted to disrupt the binding site of the transcription factor *ZNF135* and concordant effects on chromatin accessibility are seen in the ATAC-seq tracks for a subset of samples by genotypes (as seen in Fig S21).

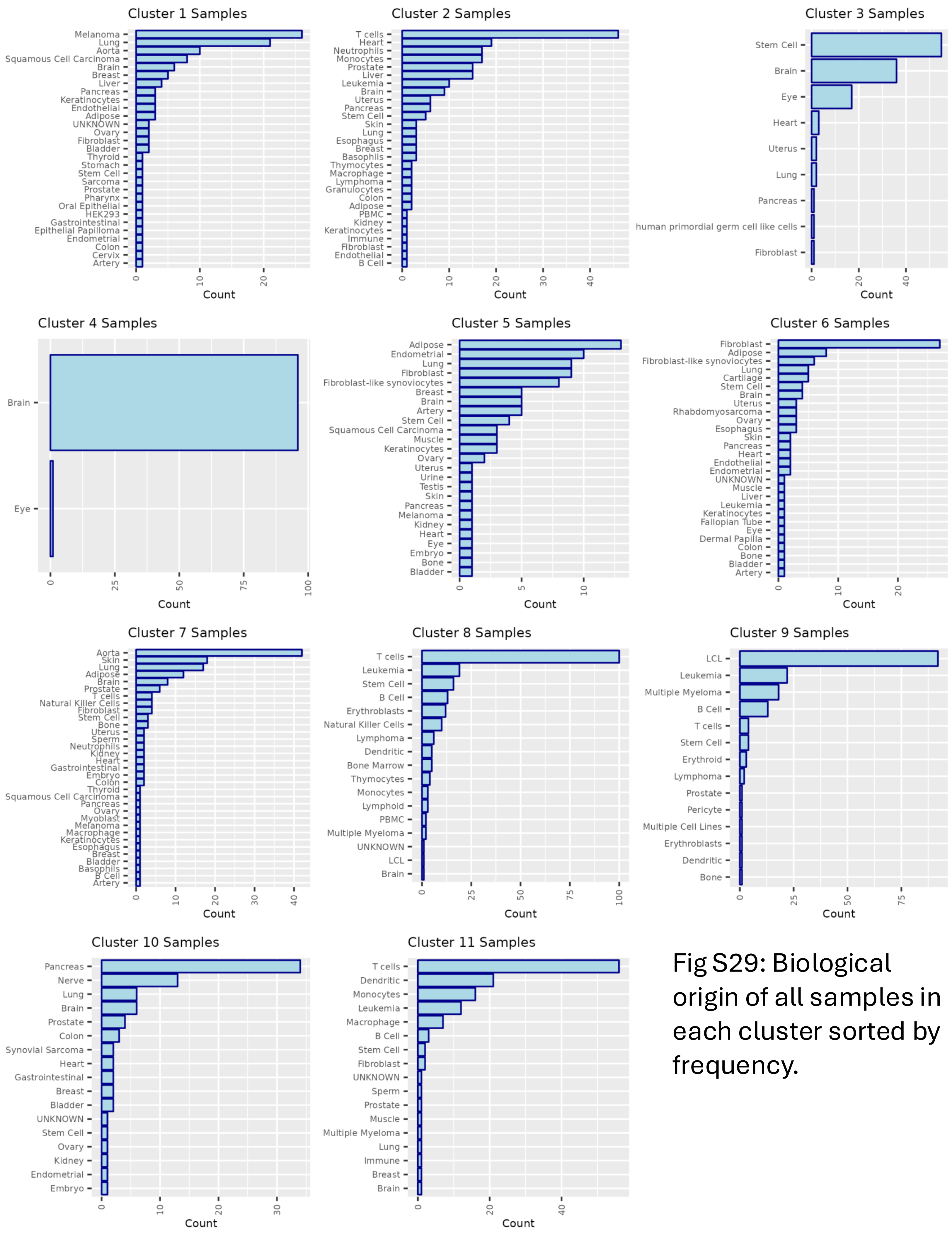

Fig S29: Biological origin of all samples in each cluster sorted by frequency.

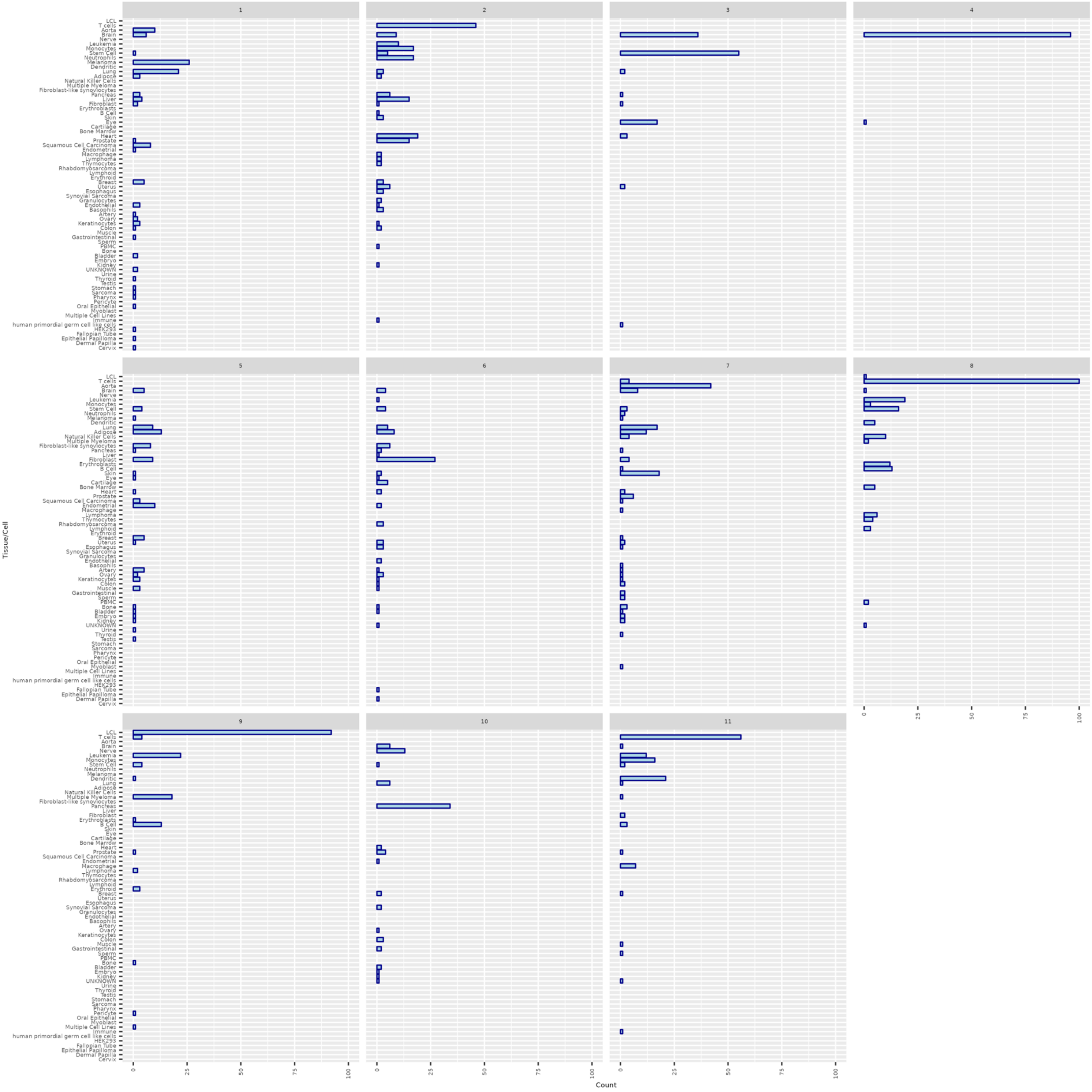

Fig S30: Proportion of each cluster comprised of each biological sample type.

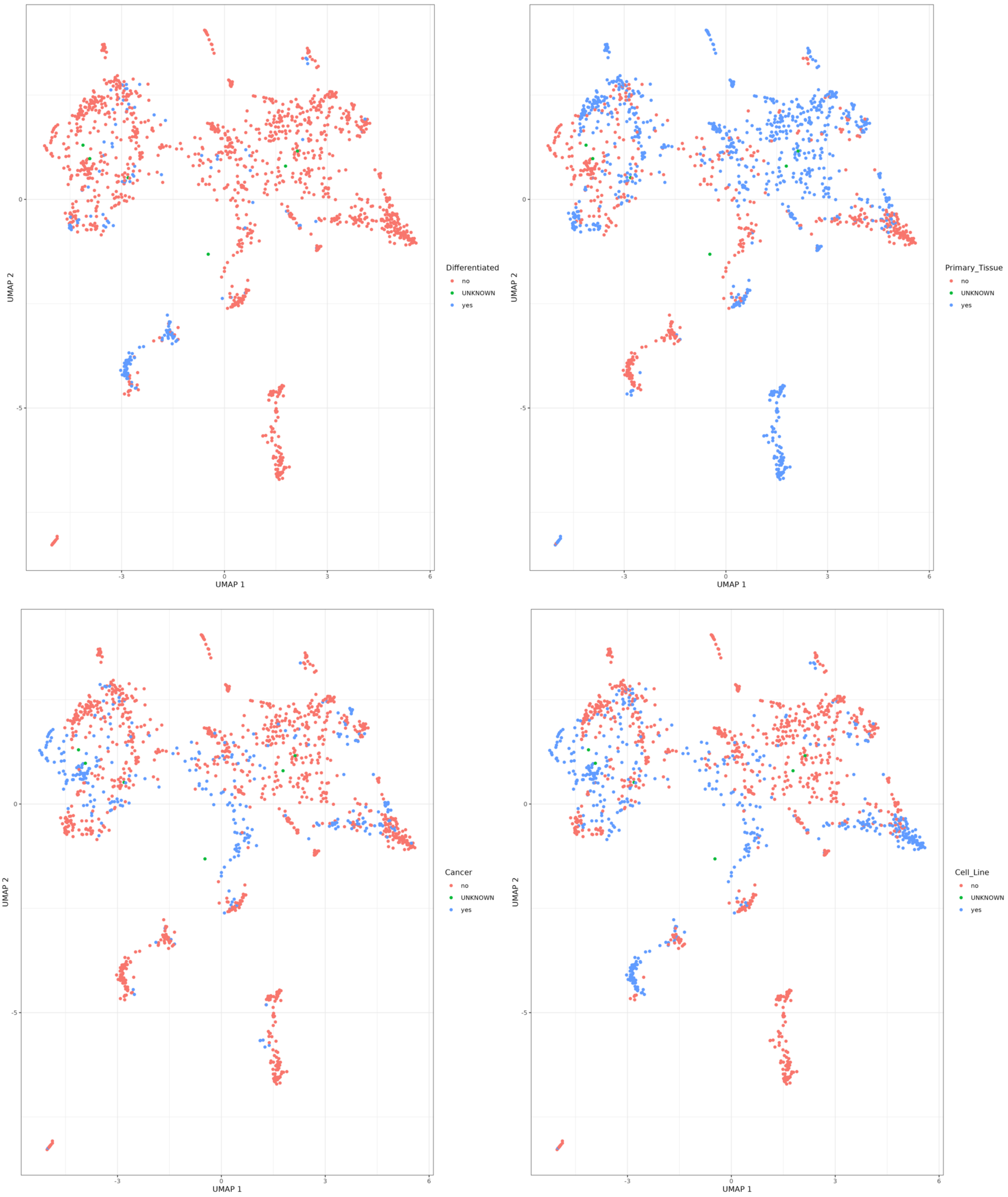

Fig S31: Chromatin accessibility profile clustering results colored by sample metadata other than biological origin.

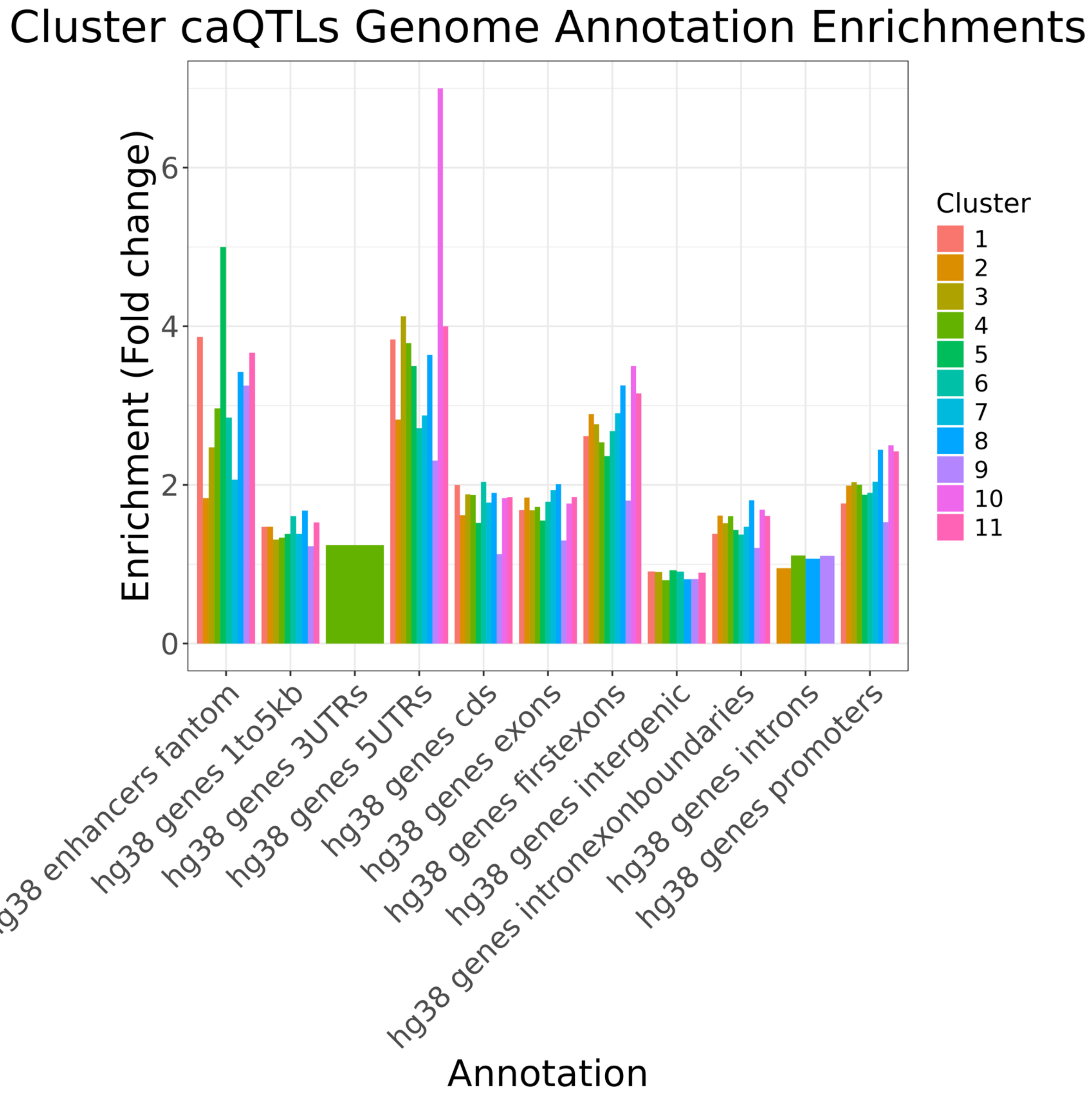

Fig S32: Enrichment of caQTL peaks from each cluster, compared to matched random control regions, in various genomic annotation categories.

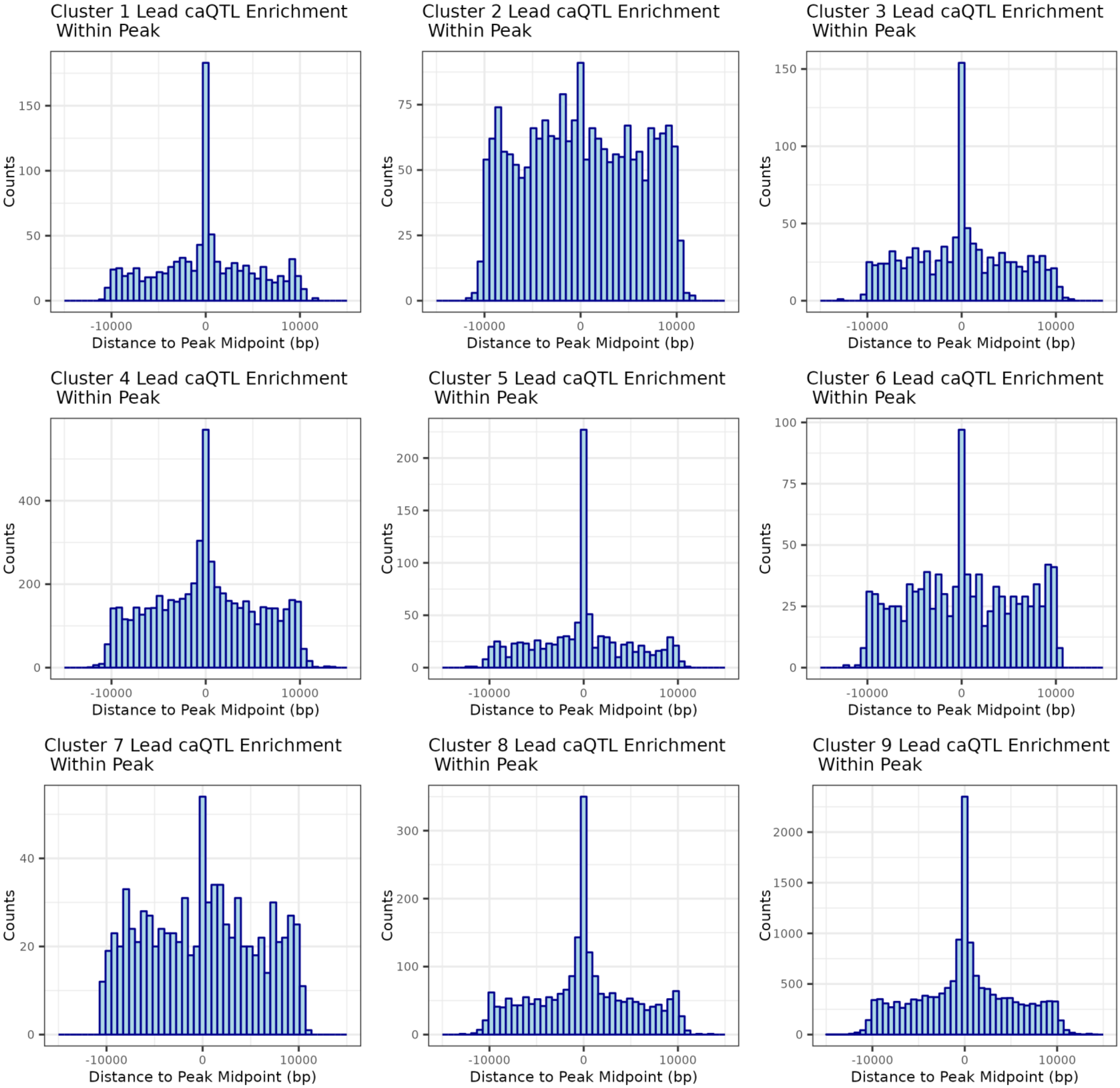

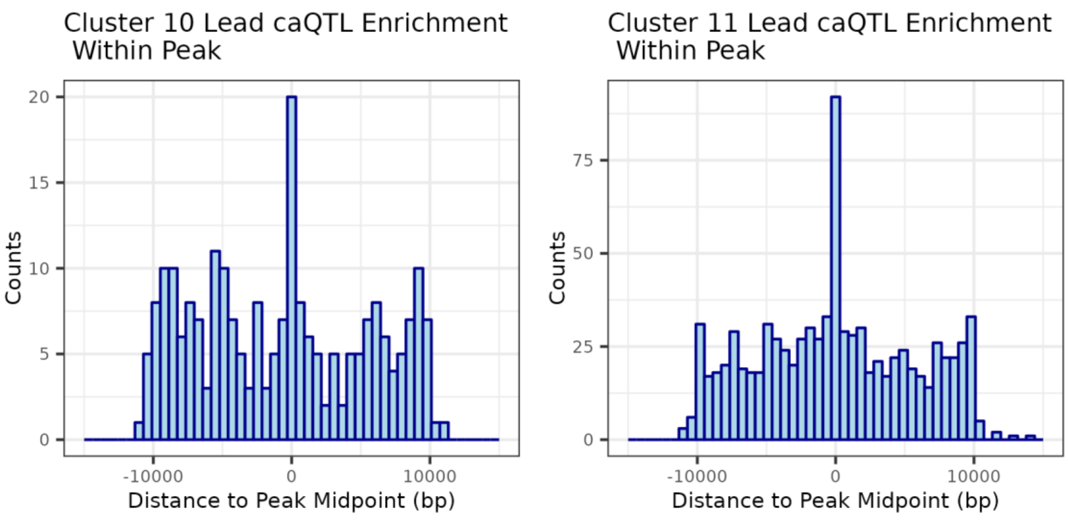

Fig S33: Distance from cluster lead caQTL variant to midpoint of caQTL peak showing elevation of caQTL variant within the identified chromatin accessibility peak.

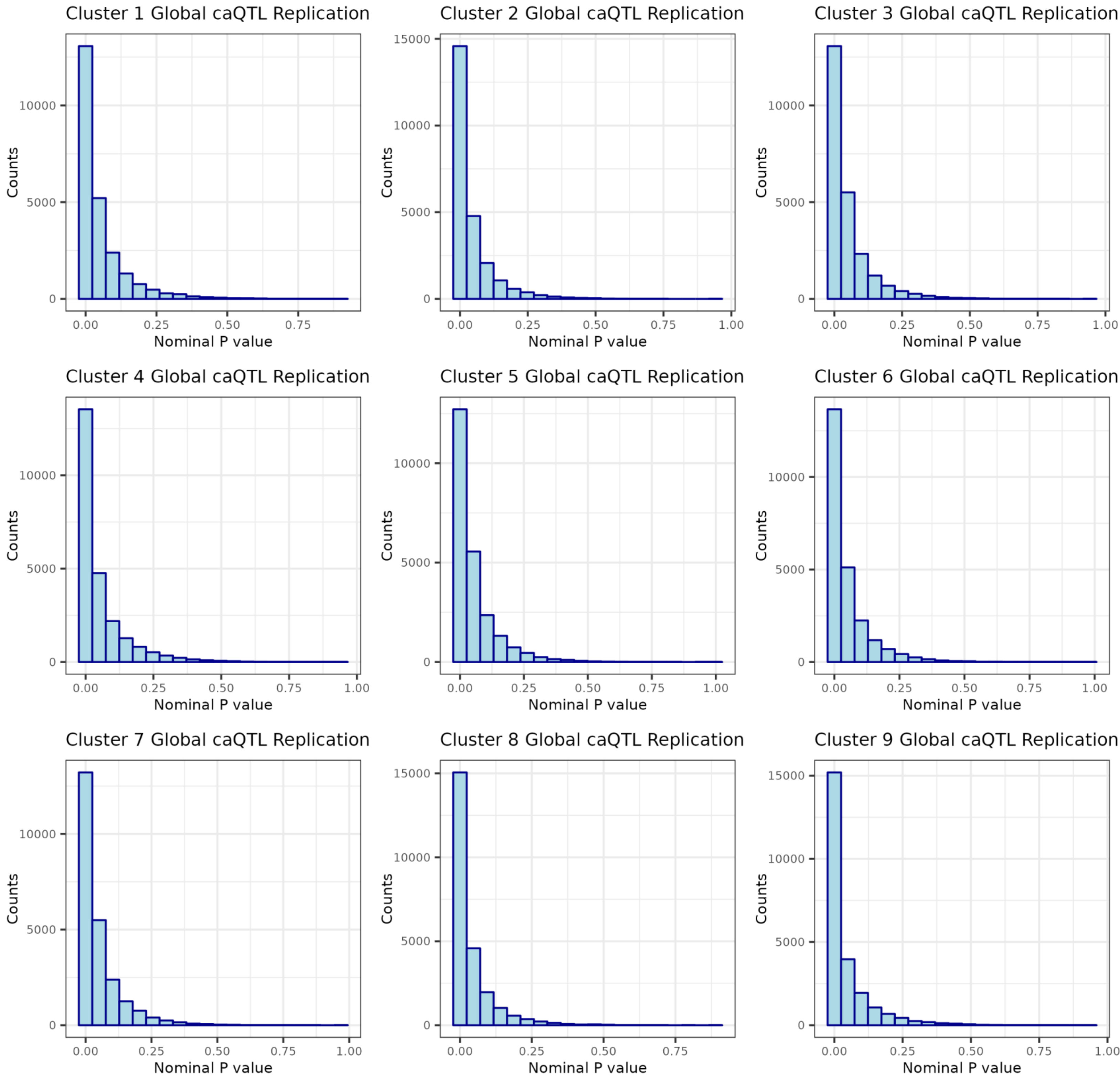

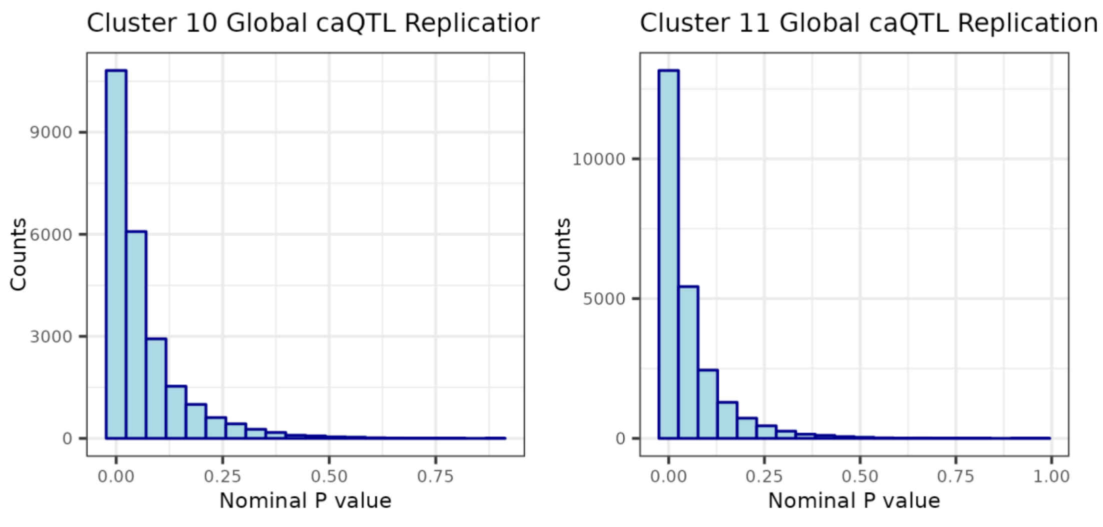

Fig S34: Cluster lead caQTL p values for global caQTL peaks plotted to show replication.

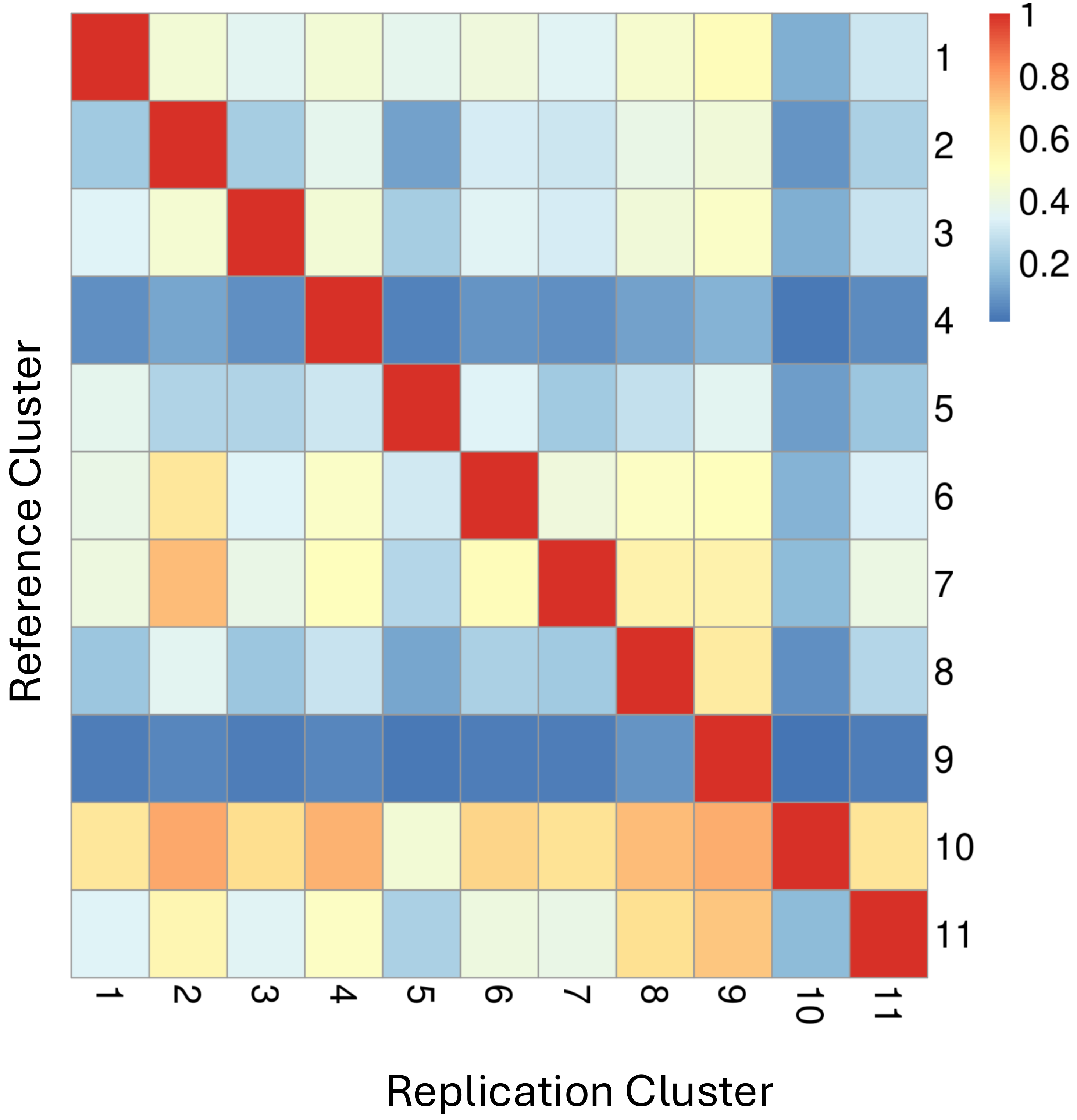

Fig S35: Sharing of caQTL peaks across each cluster. Plotted across each row is the proportion of caQTL peaks, relative to the total number of reference cluster caQTL peaks, that are also identified as a caQTL peak in the replication cluster.

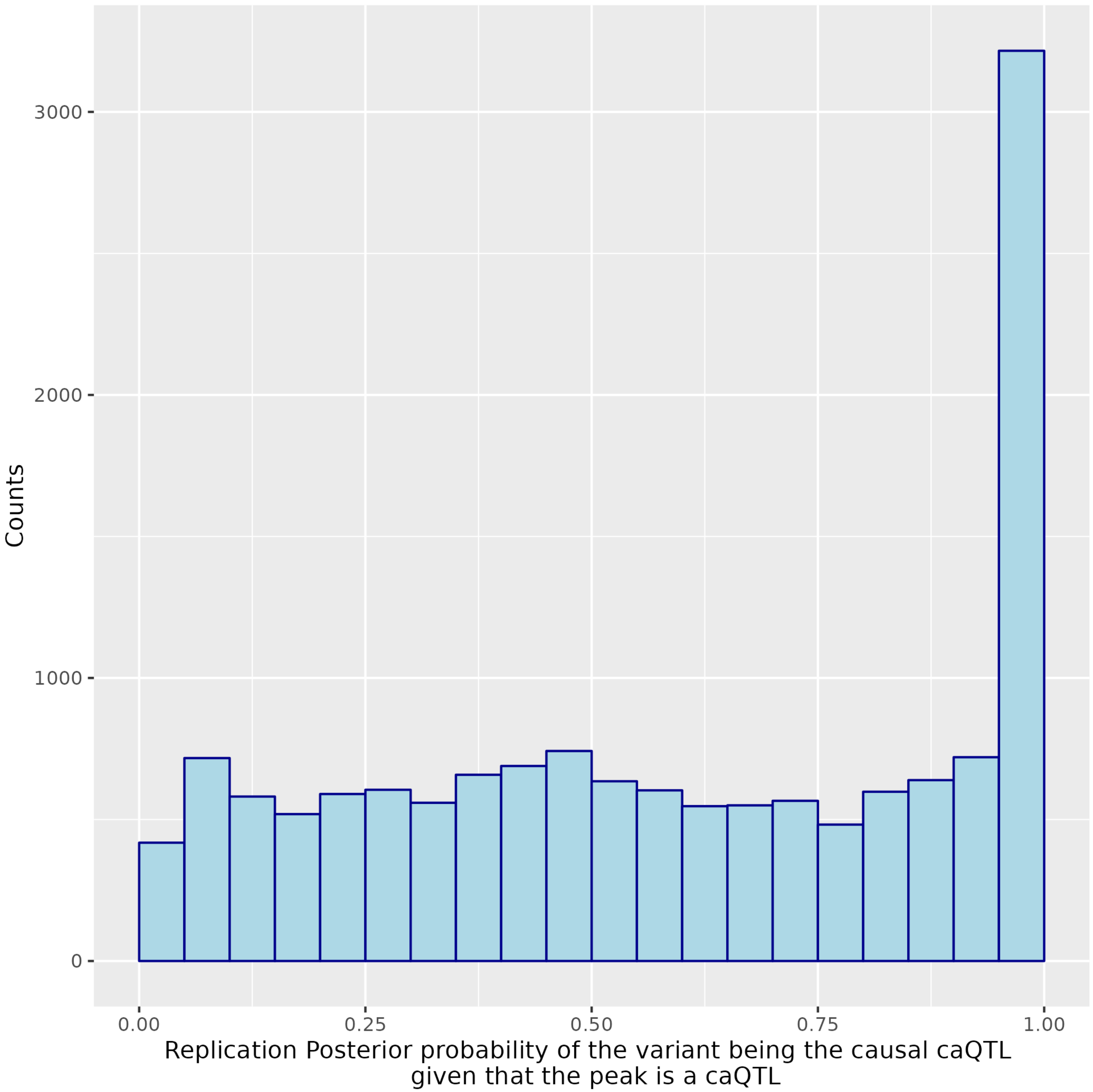

Fig S36: Cluster 9 lead caQTL peaks matched to peaks from external dataset, from which 72 cluster 9 samples originated. Posterior probability of cluster 9 lead caQTL variant being causal for caQTL peak in exterior study plotted.

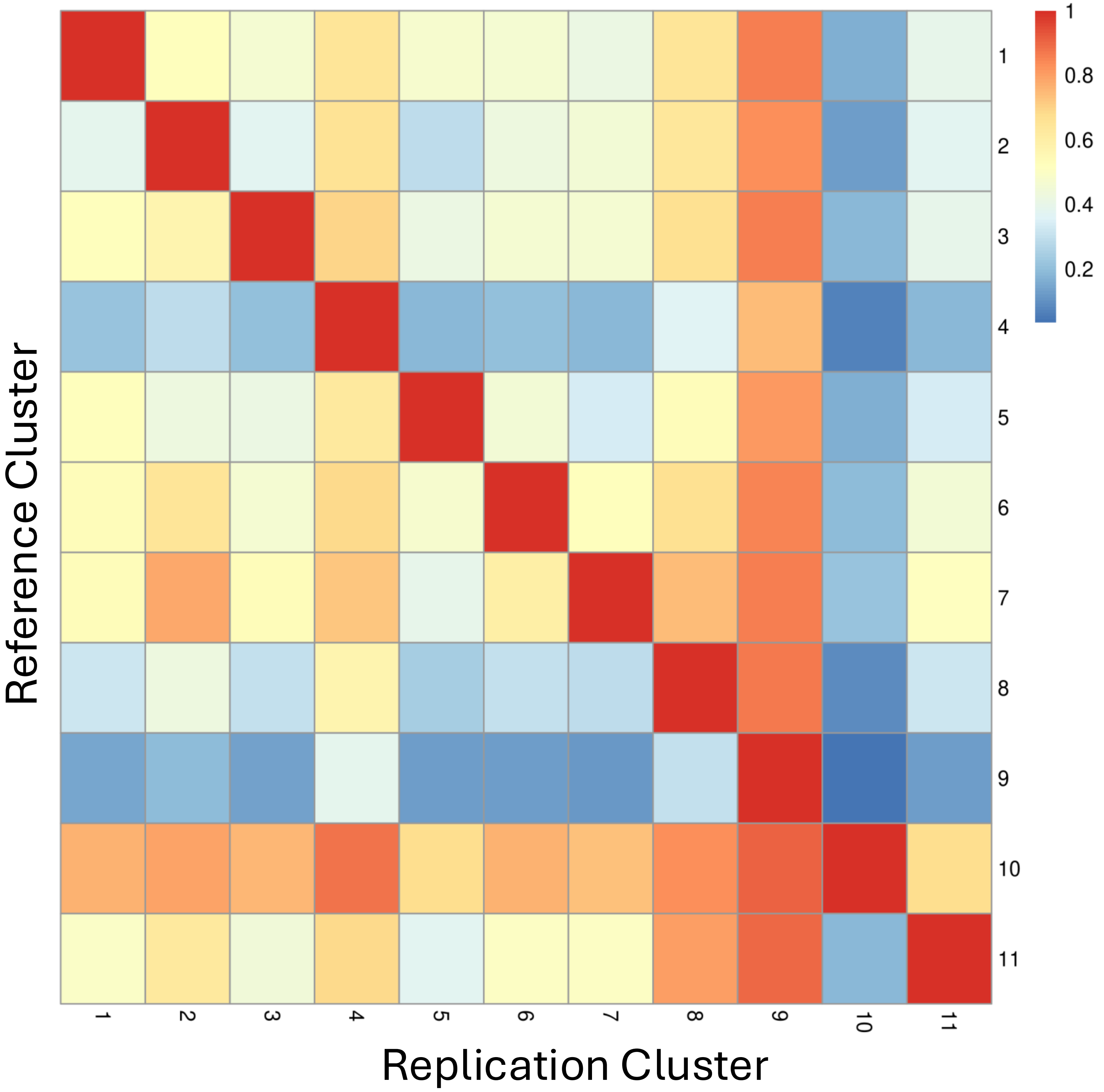

Fig S37: Cluster caQTL and eQTL colocalization eGene sharing across clusters. Plotted is the proportion of total colocalizing eGenes in reference cluster, relative to the total number of reference cluster colocalizing eGenes, that also colocalized in the replication cluster.

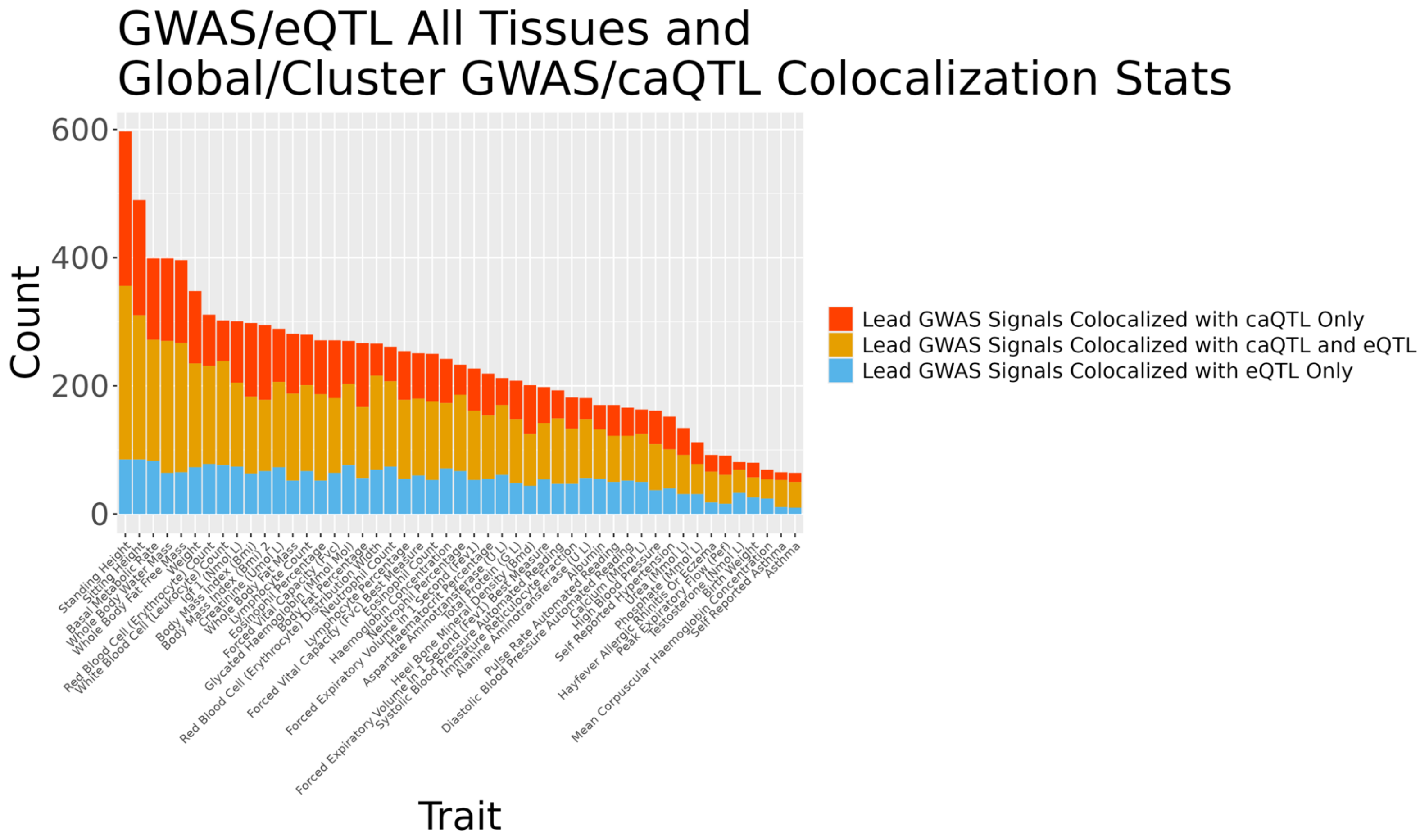

Fig S38: For each GWAS trait, independent lead GWAS variant signals were checked for colocalization with global/cluster caQTL and eQTL signals across all GTEx tissues. Plotted is the number of unique lead GWAS signals per colocalization group, as multiple caQTL peaks, eGenes, etc. can colocalize with the same lead GWAS signal. Traits with greater than 50 colocalizing lead variants shown.

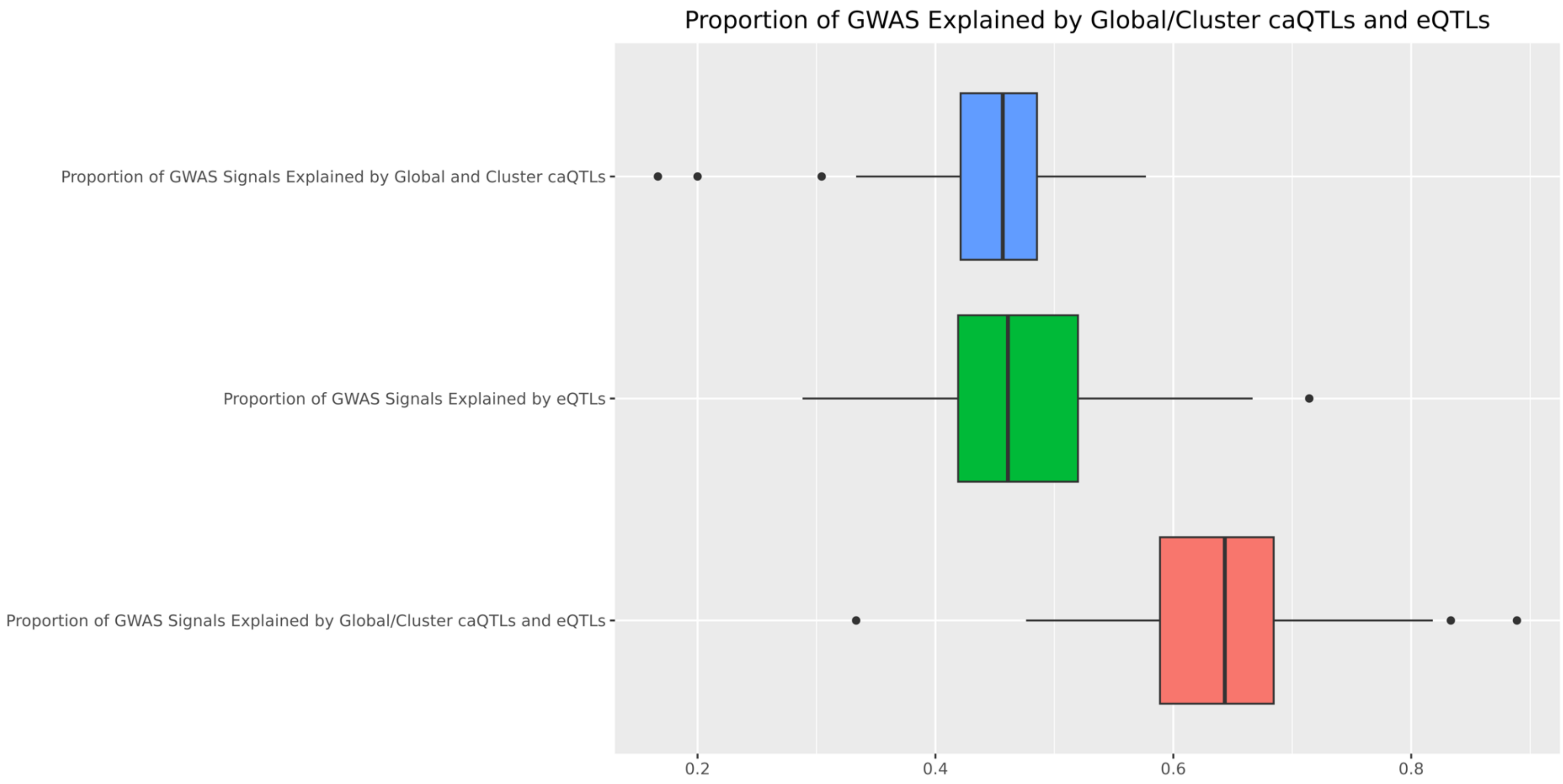

Fig S39: Colocalizations were performed between global/cluster caQTL/GWAS and eQTL/GWAS. Each GWAS signal was checked to see if it colocalized exclusively with caQTLs, eQTLs, or colocalized with both. Proportion of tested GWAS signals that colocalized in each category are plotted.

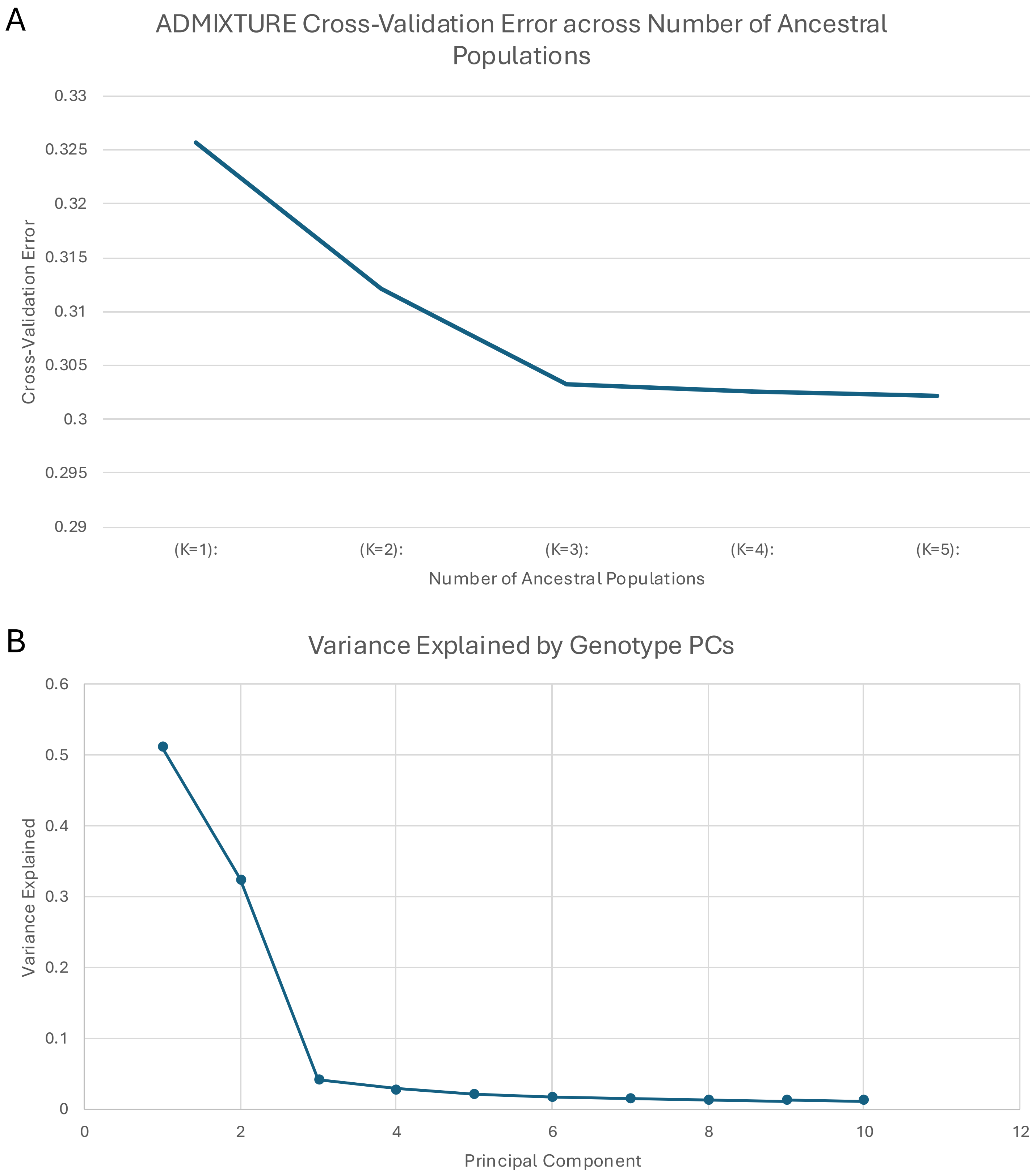

Fig S40: A) ADMIXTURE was used to infer the number of ancestral populations included in our samples using the cross-validation procedure to minimize cross-validation error. B) The first three genotype PCs explain ~88% of the genotype variance and form an elbow in the plot of PCs versus variance explained.

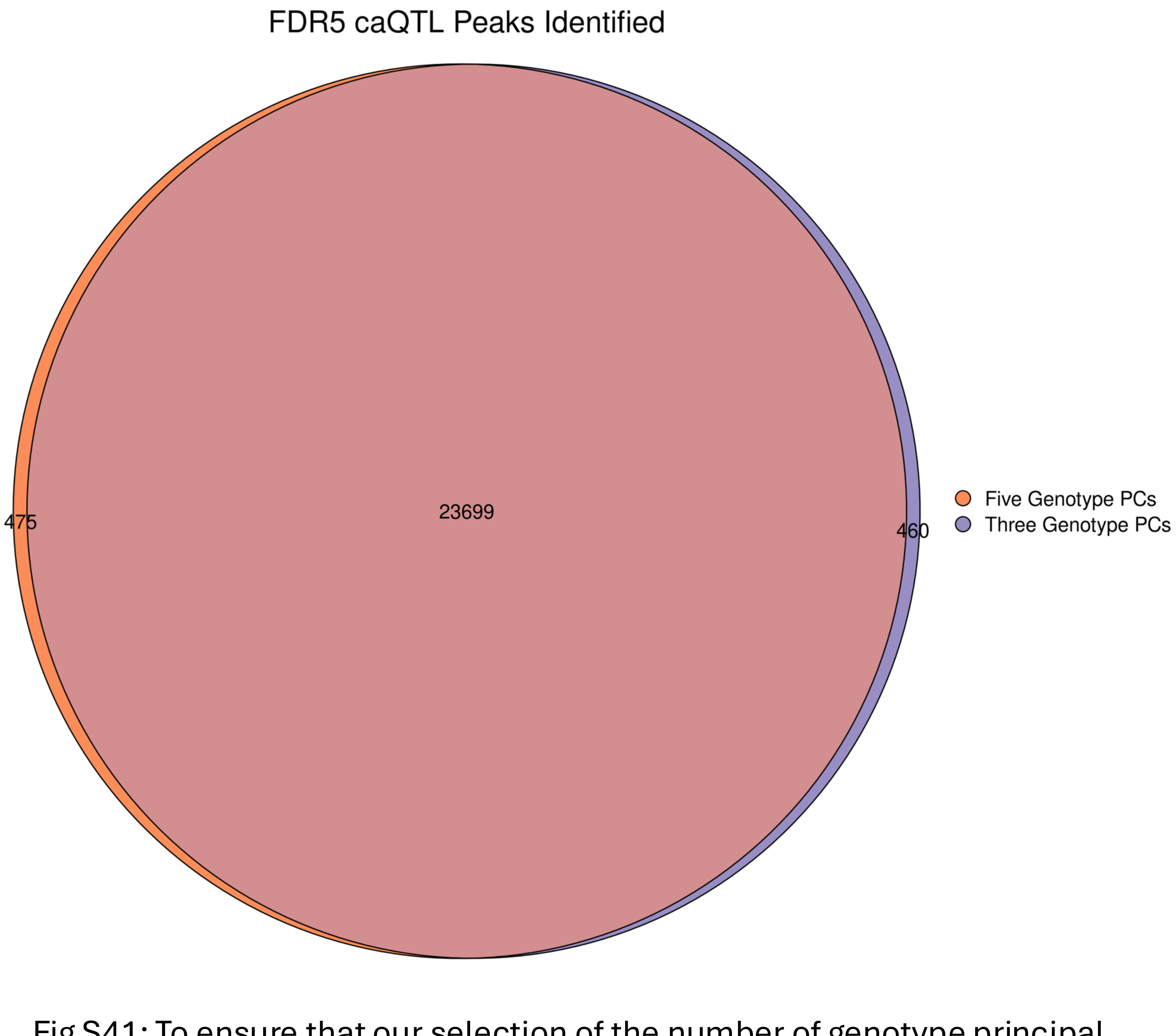

Fig S41: To ensure that our selection of the number of genotype principal components (PCs) included as covariates in caQTL mapping was robust, we mapped caQTLs with either the first three or five genotype PCs. We found a high concordance of caQTL peaks identified across these analyses.

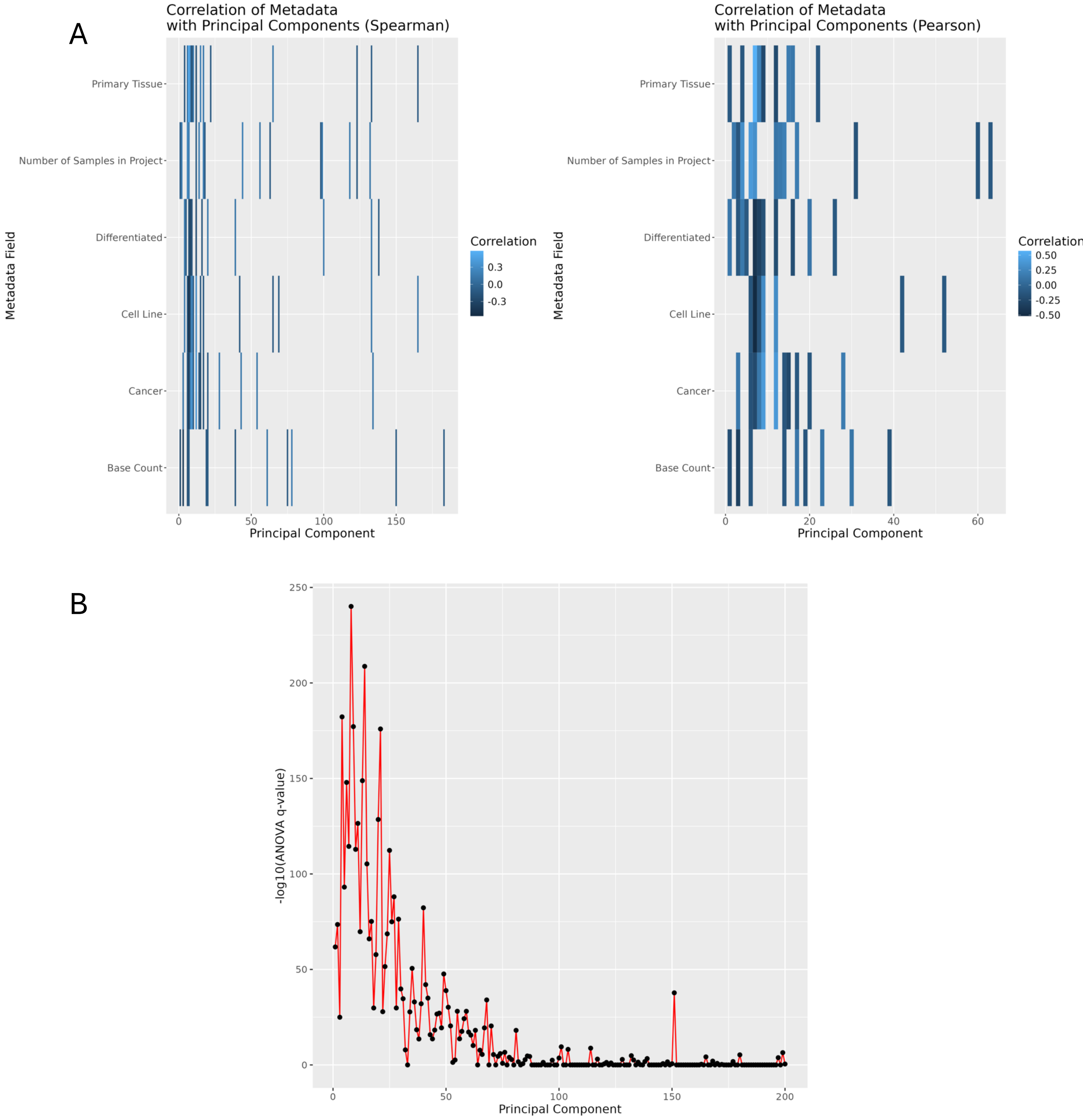

Fig S42: Correlation of PCs with metadata. A) Correlation of count matrix PCs with sample metadata. Significant correlations (q<0.05) after Bonferroni correction are shown. B) Multivariate Analysis Of Variance (MANOVA) was performed between PCs and sample cell type. P-values were Bonferroni corrected and –log10(q-values) are plotted.

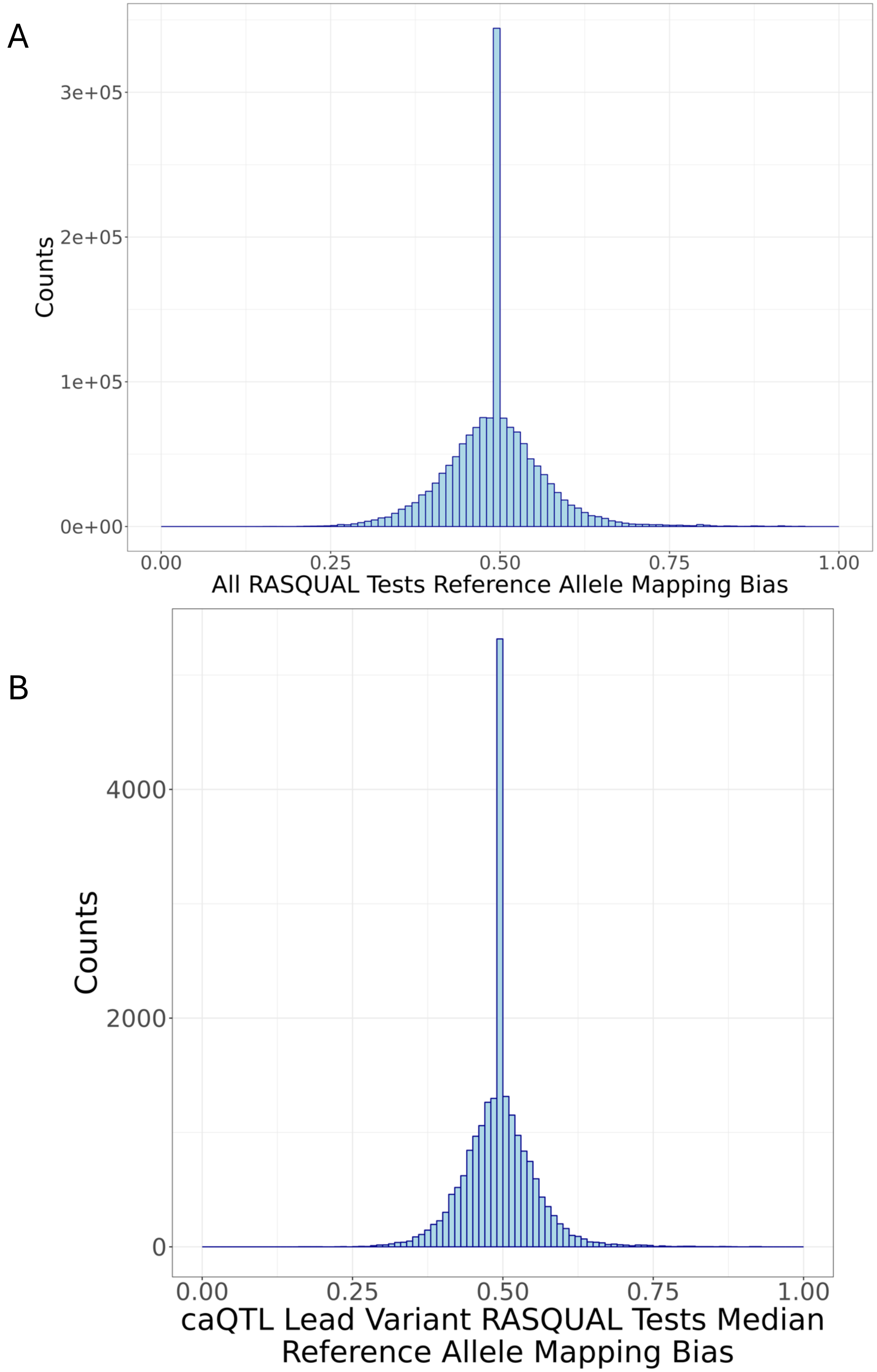

Fig S43: RASQUAL was used to map caQTLs in a subset of samples at the FDR5 caQTL peaks identified with tensorQTL. RASQUAL reports a reference allele mapping bias (Phi) score for each variant tested. A) Reference allele mapping bias for all tested variant-peak pairs. B) Reference allele mapping bias for all lead caQTL variants from tensorQTL analysis.
